# Distinctive epigenomes characterize glioma stem cells and their response to differentiation cues

**DOI:** 10.1186/s13059-018-1420-6

**Published:** 2018-03-27

**Authors:** Dan Zhou, Bonnie M. Alver, Shuang Li, Ryan A. Hlady, Joyce J. Thompson, Mark A. Schroeder, Jeong-Heon Lee, Jingxin Qiu, Philip H. Schwartz, Jann N. Sarkaria, Keith D. Robertson

**Affiliations:** 1 Department of Molecular Pharmacology and Experimental Therapeutics, Mayo Clinic, Rochester, MN USA; 2 Department of Radiation Oncology, Mayo Clinic, Rochester, MN USA; 3 Department of Biochemistry and Molecular Biology, Mayo Clinic, Rochester, MN USA; 4 Epigenomics Translational Program, Mayo Clinic, Rochester, MN USA; 5 Department of Pathology and Laboratory Medicine, Roswell Park Cancer Institute, Buffalo, NY USA; 6 National Human Neural Stem Cell Resource, Children’s Hospital of Orange County Research Institute, Orange, CA USA; 7 Center for Individualized Medicine, Mayo Clinic, Rochester, MN USA; 8 Mayo Clinic Cancer Center, Mayo Clinic, Rochester, MN USA

**Keywords:** Epigenome, 5mC, 5hmC, 5fC, 5caC, Glioma stem cell, Neural stem cell, Enhancer, Differentiation

## Abstract

**Background:**

Glioma stem cells (GSCs) are a subpopulation of stem-like cells that contribute to glioblastoma (GBM) aggressiveness, recurrence, and resistance to radiation and chemotherapy. Therapeutically targeting the GSC population may improve patient survival, but unique vulnerabilities need to be identified.

**Results:**

We isolate GSCs from well-characterized GBM patient-derived xenografts (PDX), characterize their stemness properties using immunofluorescence staining, profile their epigenome including 5mC, 5hmC, 5fC/5caC, and two enhancer marks, and define their transcriptome. Fetal brain-derived neural stem/progenitor cells are used as a comparison to define potential unique and common molecular features between these different brain-derived cells with stem properties. Our integrative study reveals that abnormal expression of ten-eleven-translocation (TET) family members correlates with global levels of 5mC and 5fC/5caC and may be responsible for the distinct levels of these marks between glioma and neural stem cells. Heterogenous transcriptome and epigenome signatures among GSCs converge on several genes and pathways, including DNA damage response and cell proliferation, which are highly correlated with TET expression. Distinct enhancer landscapes are also strongly associated with differential gene regulation between glioma and neural stem cells; they exhibit unique co-localization patterns with DNA epigenetic mark switching events. Upon differentiation, glioma and neural stem cells exhibit distinct responses with regard to TET expression and DNA mark changes in the genome and GSCs fail to properly remodel their epigenome.

**Conclusions:**

Our integrative epigenomic and transcriptomic characterization reveals fundamentally distinct yet potentially targetable biologic features of GSCs that result from their distinct epigenomic landscapes.

**Electronic supplementary material:**

The online version of this article (10.1186/s13059-018-1420-6) contains supplementary material, which is available to authorized users.

## Background

Glioblastoma (GBM) is the most common and malignant glioma subtype in adults [1]. Average survival is only about 15 months, making GBM one of the most aggressive cancers. It has been hypothesized that distinct subpopulations with a survival advantage and enhanced resistance to therapy are contained within the bulk tumor, yet this property is not readily unveiled by gross histopathologic examination. Genetic and transcriptomic studies have subtyped GBM based on expression profiling into classical, mesenchymal, proneural, and neural, with each subtype characterized by distinct amplification/loss/mutation of genes such as *EGFR*, *NF1*, *CDKN2A*, and *PTEN* [2]. Single-cell RNA sequencing (RNA-seq) revealed that an individual tumor contains a spectrum of GBM subtypes, suggesting that intratumoral heterogeneity is extensive [3, 4].

Theories underlying tumor evolution support the marked heterogeneity observed within individual gliomas. The cancer stem cell (CSC) theory postulates existence of a subpopulation of tumor cells residing at the apex of the hierarchy, propagating tumor formation in a hierarchical manner. CSCs are characterized by an ability to self-renew and differentiate, contributing to the heterogeneity and complexity of tumors. CSCs resemble normal stem cells in a number of properties, including the ability to form spheres on non-adherent culture surfaces in serum-free media [5]. Relative quiescence coupled with low levels of apoptosis and slow cell cycling contribute to CSC resistance to chemotherapy, while their asymmetric division gives rise to poorly differentiated daughter cells that facilitate tumor recurrence [6, 7]. Oncogenic mutations occurring in normal stem cells could contribute to their malignant transformation into cancer stem cells. Early studies showed that manipulating the ARF/p53 pathway in neural stem/progenitor cells resulted in high-grade glioma [8, 9]. Glioma stem cells (GSCs) identified within bulk GBM tumors might therefore share biologic similarities with normal neural stem cells, but also possess distinct genetic and epigenetic alterations that underpin their malignant growth potential. Elucidating such differences is key to improving therapeutic targeting, efficiency, and specificity; however, such targetable epigenetic and transcriptomic differences between NSCs and GSCs remain largely unknown.

GSCs acquire both genetic and epigenetic mutations [10]. Epigenetic changes, like genetic changes, act as driver events in transformation or collude with genetic events to drive transformation. In contrast to genetic alterations, epigenetic changes are, in principle, reversible and therefore represent attractive therapeutic targets. DNA methylation (5mC, mediated by the DNA methyltransferases DNMT1, 3A, and 3B) and DNA hydroxymethylation (5hmC, mediated by the ten-eleven translocation TET1, 2, 3 family) are extensively disrupted in GBM. The TET protein family is responsible for producing 5hmC, 5-formylcytosine (5fC), and 5-carboxylcytosine (5caC); and like the DNMTs, their expression is tightly regulated during development. Tumor cells, including GBM, are in general depleted for both 5mC and 5hmC, accompanied by reduced TET expression [11, 12]. GBM patients with G-CIMP (glioma-CpG island hypermethylator phenotype resulting from *IDH1/2* mutation), or overall elevated 5mC and/or 5hmC levels, exhibit better clinical outcome [13, 14]. Although DNA methylation and transcriptional alterations have been studied extensively in GBM, details of the epigenetic reprogramming events that contribute to and/or define glioma stem cells, especially in terms of the TET-regulated DNA marks and their association with enhancer function, remain poorly characterized. In the current study, we aimed to define epigenetic abnormalities linked to GSC stemness and their regulation in response to differentiation cues relative to those characteristics of neural stem cells, to identify features unique to GSCs that may serve as novel therapeutic targets.

## Results

### Characterization and global assessment of DNA epigenetic modifications in glioma stem cells

**Fig. 1:**
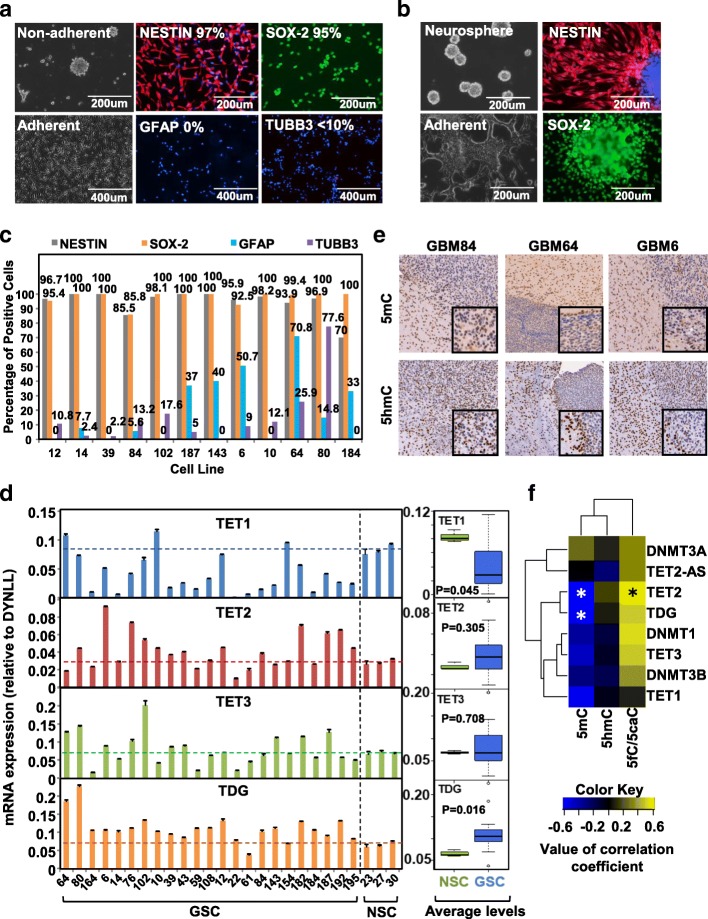
Cell line characterization and global analysis of DNA epigenetic modifications. **a** Representative immunofluorescence staining (IF) of PDX-derived glioma stem cell (GSC) line GSC12, labeled with the percent positive cells for each marker. Blue-DAPI, green-SOX-2, red-NESTIN, GFAP, and TUBB3. **b** Representative IF of a neural stem cell (NSC27) line, labeled as in (a). **c** Percent positive cells for each marker in the core set of GSCs by IF. *X-axis* denotes the cell line. *Y-axis* denotes the percent positive cells. **d** TET1, TET2, TET3, and TDG expression by quantitative reverse transcription polymerase chain reaction (qRT-PCR), normalized to DYNLL and expressed as the mean ± SEM. *Dashed line* marks the average expression level of each gene in NSCs. *Boxplots* depict the distribution of TET expression in NSC and GSC. *X-axis* denotes the cell line. *Y-axis* denotes the normalized messenger RNA (mRNA) expression. **e** Immunohistochemical quantification of 5mC and 5hmC at the margin of the tumor in PDX GBM6, GBM64, and GBM84 as indicated on *top* of the image. Images are enlarged to view the difference in staining intensity between tumor and surrounding normal murine tissue. **f** A *heatmap* of the correlation between the global level of DNA marks measured by mass spectrometry and expression of DNMTs and TETs. *Blue*: negative correlation; *yellow*: positive correlation. *Asterisk* – significant correlation with *P* < 0.05

Primary patient GBM tissue was transplanted into mice as described previously to create patient derived xenografts (PDX) [15]. The stem-cell population was isolated from 22 different PDX tumors (Additional file 1: Tables S1 and S2), whereas neural progenitor lines, a hypothetical origin of GSCs, were isolated from fetal brain (NSC23, NSC27, and NSC30). Both cell types formed neurospheres in non-adherent culture conditions but attached onto a laminin/ fibronectin-coated surface (Fig. 1a left, Fig. 1b left). Immunofluorescence (IF) for stem markers NESTIN and SOX-2 were detected in NSCs (Fig. 1b). The stemness of GSCs was assessed by staining for NESTIN, SOX-2, and CD44, and lineage markers GFAP (astrocyte), TUBB3 (neuron), and GALC (oligodendrocyte) (Fig. 1a, Additional file 1: Table S1, top). Twelve GSC lines containing a high proportion of stem marker-positive and lineage marker-negative cells were used for further molecular analyses (Fig. 1c, Additional file 1: Table S2). Indeed, consistent with these stem and lineage marker expression patterns, genome-wide 5mC patterns based on 450 k array analysis (run on 22 GSC lines and eight glioma cell lines) also largely segregates these 12 GSC lines into a distinct group (Additional file 2: Figure S1A).

Expression of the DNA modification machinery in GSCs is variable and distinct from that in NSCs. TET1 transcription is overall downregulated, whereas TET2 and TDG are upregulated in the majority of GSCs; TET3 is the most variably expressed (Fig. 1d). Global quantification of DNA marks using mass spectrometry (MS) [16] show that NSCs overall possess higher amounts of 5mC and 5hmC, but lower levels of 5fC, compared to GSCs (Additional file 2: Figure S1B). Their levels were further compared to those of established serum cultured glioma cell lines T98G and A172, and normal brain tissue. Notably, the cancer cell lines exhibit lower levels of 5mC compared to normal tissue (Additional file 2: Figure S1C top), but much reduced levels of 5hmC and 5fC (Additional file 2: Figure S1C middle and bottom). 5caC levels are not shown because our LC-MS/MS method failed to capture the 5caC-containing fractions from all samples analyzed. In keeping with these findings, PDX tissue derived from GBM6, GBM64, and GBM84 exhibit reduced immunohistochemical (IHC) staining intensity in the nucleus for both 5mC and 5hmC, relative to surrounding normal tissue (Fig. 1e). We correlated transcription level of the TETs to the level of each DNA mark (Additional file 2: Figure S1D) and observed that TET2 and TDG expression are significantly negatively correlated with 5mC level, while TET2 is positively correlated with 5fC (Fig. 1f, Additional file 2: Figure S1D), suggesting that TET2 expression contributes to the increased level of 5fC in GSCs.

### Transcriptome profiling reveals novel gene networks and GSC-specific targets

**Fig. 2:**
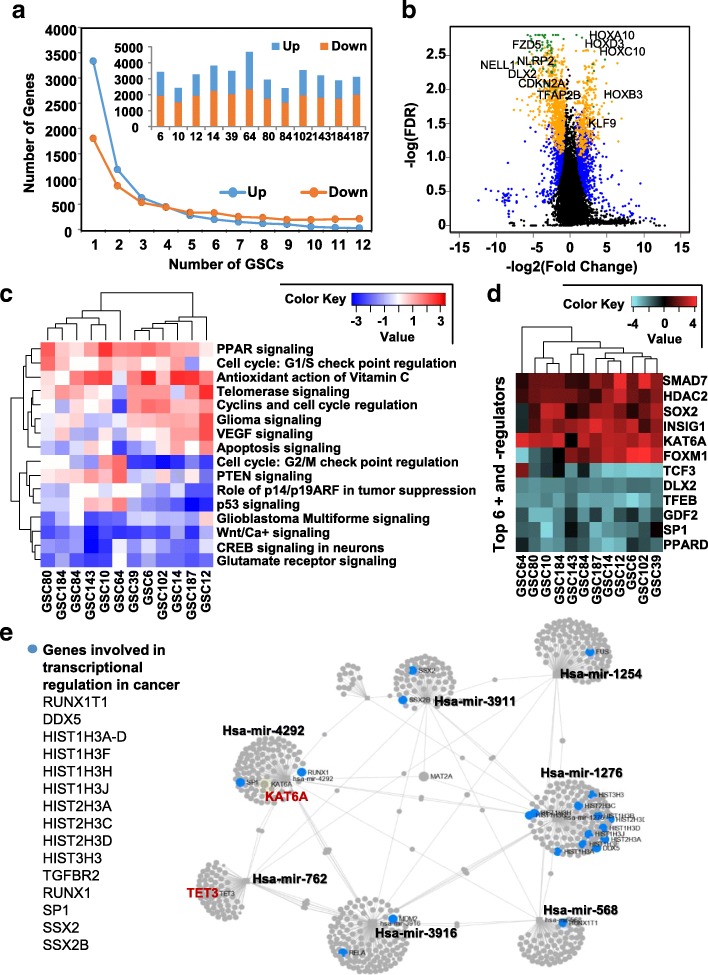
GSC-specific transcriptomic profiles relative to NSCs. **a** *Line plot* showing the number of deregulation events shared among different GSC lines. The embedded *bar graph* shows deregulation events in each GSC (as labeled on *X-axis*) compared to NSC. *Blue*: upregulation; *orange*: downregulation. **b** *Volcano plot* illustrating differential expression in GSC compared to NSC. *Black dots*: genes not differentially expressed. *Blue dots*: deregulated genes in any GSC. *Orange*: genes deregulated in ≥ 6 GSCs. *Green*: genes common to all 12 GSC lines. *X-axis* denotes the log2-transformed expression fold change. *Y-axis* denotes the log10-transformed false discovery rate. **c** Ingenuity pathway analysis (IPA) comparative analysis of the top most commonly activated (*red*) and inhibited (*blue*) biological pathways in GSCs relative to NSCs. The pathways are annotated to the right while GSC IDs are at the bottom of the *heatmap*. *Blue*: inhibition of the pathway. *Red*: activation of the pathway. **d** IPA analysis of the top activated (*red*) and inhibited (*teal*) upstream regulators in GSC relative to NSC. The regulators are annotated to the right; GSC number is at the bottom of the *heatmap*. **e** MicroRNA (miRNA) networks and their targeted genes (expressed as *gray dots*). Genes involved in transcriptional regulation in cancer are highlighted in *blue dots* in the network and listed at the left. KAT6A and TET3 are highlighted as targets of mir4292 and mir762, respectively

Transcriptional signatures of NSC23 and NSC27 are highly similar, but distinct from that of GSCs; GSCs do not cluster by the expression subtype of their primary tumor (Additional file 2: Figure S2A, S2B). Therefore, a pooled profile of NSC23 and NSC27 was compared to each GSC to assess similarities and differences among GSCs. Overall, downregulation events occurred more frequently than upregulation events in GSC compared to NSC (Fig. 2a, embedded bar graph). Notably, although each GSC harbors a substantial number of unique deregulated genes, a large proportion are still shared by a subset of GSCs, especially for downregulation events (Fig. 2a, Additional file 1: Table S3). Upregulation of the HOX gene clusters has been previously reported in three GSC lines [17]; our study confirms that HOX gene upregulation is indeed a highly conserved event in the GSC population in our larger dataset (Fig. 2b). In addition, genes involved in apoptotic processes, such as NLRP2 and TFAP2B, proliferation inhibition, such as CDKN2A and FZD5, and neural system development, such as DLX2 and NELL1, are downregulated in nearly all GSCs compared to NSCs. We further validated our analysis by examining expression of several deregulated genes by real-time quantitative reverse transcription polymerase chain reaction (qRT-PCR). Expression of these genes is consistent with the RNA-seq results (Additional file 2: Figure S2C). Although gene deregulation events appear heterogenous across GSCs, they converge on several common pathways, including cell proliferation, DNA damage response, apoptosis, and cancer development (Fig. 2c, Additional file 1: Table S4 for a complete list of ontology terms). Predicted upstream regulators of these pathways show convergent targets, including KAT6A, a known upstream regulator of the HOX gene clusters, and TFEB, a central transcriptional inhibitor of the lysosomal axis (Fig. 2d, Additional file 2: Figure S2D). Chromatin remodeling factors, such as SMAD7 and HDAC2, are additional conserved upstream regulators in GSCs.

We identified several highly conserved downregulation events for microRNAs (miRNAs) in GSCs, including hsa-mir-31 [18] and hsa-mir-210 [19] (conserved in 6–9 GSCs), that have been reported to be downregulated in GBM, and seven poorly characterized miRNAs deregulated in GBM (conserved in > 9 GSCs) (Additional file 1: Table S5, validation of select miRNAs in Additional file 2: Figure S2C). GSC-downregulated miRNAs are potentially involved in processes modulating nucleosome assembly, chromatin silencing, epigenetic regulation, and telomere organization through their target genes (Additional file 1: Table S5, Additional file 2: Figure S2E). Coordination of these deregulated miRNAs may together lead to transcriptional deregulation in cancer via a large network (Fig. 2e), in which KAT6A and TET3 may also participate. Because we could not directly derive prognostic value of these miRNAs, we examined expression of genes predicted to be targets of the deregulated miRNAs for links to patient survival. A total of 193 and 543 genes are significantly associated with better and worse survival, respectively, leveraging clinical information and RNA-seq data of TCGA GBM samples (Additional file 1: Table S6 for complete list). We identified 20 GSC-upregulated miRNA-targeted genes associated with significantly reduced 5-year survival in GBM patients, including previously identified glioma-promoting genes HMGA2 [20], MYO1C [21], RGS4 [22], and several novel targets, such as HPCAL1, IMPDH1, and YWHAG (Additional file 1: Table S5, orange-colored genes, Additional file 2: Figure S2F). Therefore, deregulation of miRNAs in GSCs may contribute to poor outcome in part through modulating expression of these miRNA-targeted loci.

### Ectopic gain and loss of enhancer marks in GSCs contribute to deregulated gene expression

**Fig. 3:**
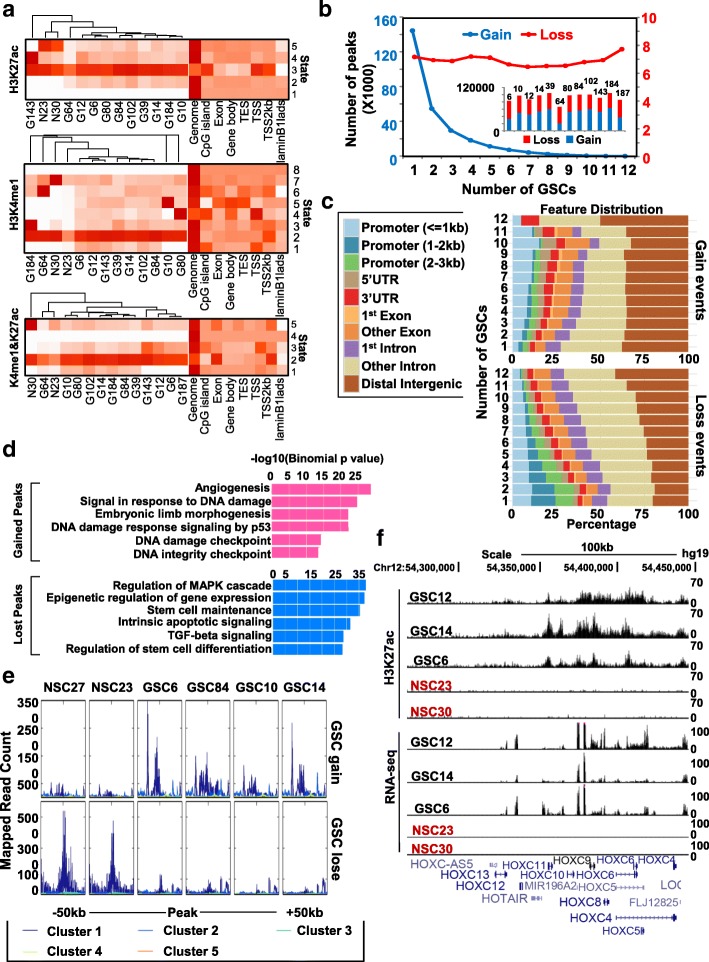
Links between differentially modified enhancers and gene regulation events. **a** ChromHMM modeling of H3K27ac (*top*), H3K4me1 (*middle*), and co-marked regions (*bottom*) in GSCs and NSCs, and their co-localization with genomic features as labeled at the *lower right*. State numbers are assigned at the *far left*. Abbreviations are used to describe cell identities. G GSC, N NSC. Enrichment level increases as color migrates from *white* to *dark red*. **b** *Line plot* showing the number of co-marked peaks gained, lost, or shared by different numbers of GSCs. Embedded stacked *bar graph* shows both gained and lost peaks for each GSC (labeled at top of each bar) compared to NSC. *Y-axis* denotes the number of peaks. *Blue*: gain; *red*: loss. **c** Genomic distribution of H3K4me1 and H3K27ac co-marked regions commonly gained (*top*) and lost (*bottom*) in different numbers of GSCs (labeled to the *left* of each bar) relative to NSCs. Genome features are color-coded in the legend bar. *Bars* are arranged in order of their conservation. *X-axis* shows the accumulated percent genomic occupancy of each feature. **d** Ontology illustrating biological processes associated with genes within 50 kb of gained (*top*, pink bars) and lost (*bottom*, blue bars) co-marked regions in GSC. *X-axis* denotes log10-transformed binomial *P* value. **e** *Tag-density plots* showing the transcript abundance of five gene clusters in a 100-kb window centered on regions gaining (*top*) and losing (*bottom*) co-marked regions in GSCs. Gene clusters were generated automatically by deepTools2 based on the enrichment pattern. **f** Browser view of H3K27ac peaks (top five tracks) and RNA-seq reads (bottom five tracks) at the HOXC locus. Scale bar: 100 kb

In general, GSCs share greater similarity in their enhancer landscape (marked by H3K27ac and H3K4me1) [23] and tend to cluster separately from normal tissue/stem cells (Additional file 2: Figure S3A). This result suggests that GSCs harbor both common and unique enhancer epigenetic features relative to non-transformed cells/tissues, and that the heterogeneity of GSCs may, in part, be attributable to their biological origin and/or culture derivation. To stratify differences between cell type and among cell lines, we leveraged the basic computational model learning system ChromHMM, and observed that most enhancers co-localize between GSCs and NSCs (e.g. H3K27ac-state 3, H3K4me1, and co-marked loci-state 2) (Fig. 3a), despite overall H3K4me1 localizing more at distal intergenic regions/promoters (1–3 kb), and H3K27ac localizing more frequently at proximal promoters (< 1 kb) (Additional file 2: Figure S3B). When referencing enhancer marks to NSC30, we observe 20–30% of H3K4me1 peaks are unique to each GSC, whereas 40–60% of H3K27ac peaks and co-peaks are unique to each GSC. This again indicates that enhancer marks are largely conserved between GSC and NSC (based on H3K4me1); only a subset of genomic regions are differentially occupied (Additional file 2: Figure S3C). Compared with NSCs, ectopic gained active enhancers occur at a similar frequency as lost enhancers in each GSC (Fig. 3b, stack bar graph). Many more unique active enhancer peaks are found in each GSC, with fewer such regions conserved in all lines (Fig. 3b, blue line). On the contrary, a relatively consistent number of active enhancers is lost in the GSCs (Fig. 3b, red line), although a growing number of common lost peaks is observed for H3K4me1 or H3K27ac alone (Additional file 2: Figure S3D). Interestingly, a step-wise increased occupancy at promoters and 5’ UTRs is observed for GSC-specific enhancer peaks, whereas lost peaks in GSC relative to NSC exhibit greater conservation in distal intergenic regions (Fig. 3c).

Gene clusters surrounding (< 50 kb) GSC-specific active enhancers (common to at least six GSCs) are primarily linked to DNA damage responses, p53 signaling, and angiogenesis (Fig. 3d top); genes near NSC-specific active enhancers (lost in at least six GSCs) are enriched for stem-cell differentiation, apoptosis, and epigenetic gene regulation (Fig. 3d bottom, Additional file 1: Table S7). We next investigated relationships between ectopic enhancers and gene transcription. Many more RNA-seq reads map to enhancer-gained regions in GSCs (common to at least six GSCs); fewer reads map to enhancer-lost regions (Fig. 3e top and bottom, cluster 1). Meanwhile, downregulation events are proportionally greater than upregulation events for genes linked to regions that lose enhancer marks in GSCs (common to at least six GSCs) and vice versa (Additional file 2: Figure S3E). These findings suggest that ectopic gain/loss of active enhancers is strongly linked to gene deregulation events in GSCs. To confirm our findings, several genomic regions positive for H3K4me1 and/or H3K27ac modifications were validated using locus-specific ChIP-qPCR. Results are consistent with ChIP-seq signals for both enhancer marks at these loci (Additional file 2: Figure S3F and G).

HOX loci are upregulated in primary tumor-isolated GSCs despite the presence of promoter hypermethylation [17]. Our data suggest that the HOX clusters acquire both H3K4me1 and H3K27ac in GSCs, which may explain the elevated HOX gene expression despite the presence of promoter methylation (Fig. 3f, Additional file 2: Figure S3H). Loss of flanking active enhancers is associated with gene repression of the PLEKHA5, PLEKHH2, and ST3GAL5 loci (Additional file 2: Figure S3H). PLEKHA5 facilitates cell migration and invasion and is linked to brain tumor metastasis via the PI3K-AKT pathway [24, 25], whereas ST3GAL5 encodes a sialyltransferase that catalyzes the formation of ganglioside GM3, a suppressor of EGFR/PI3K-AKT signaling and cell proliferation, and a inducer of apoptosis [26, 27]. Both gene clusters have known functions related to cancer cell properties, although their specific role in glioma stem cells has not been previously reported.

### Genome-wide single-base resolution mapping of DNA epigenetic marks in GSCs and NSCs

**Fig. 4:**
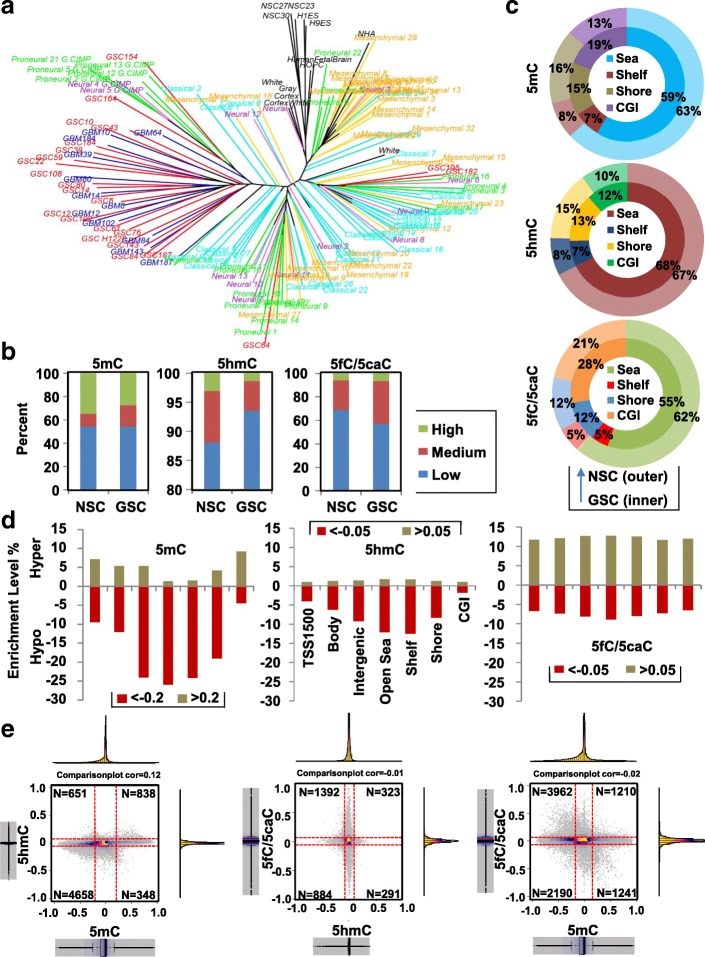
Differential distribution of DNA marks between GSC and NSC. **a** Phyloepigenetic evolutionary *tree* demonstrating the progression and relationship of the DNA methylation landscape from normal tissues/cell lines, to primary tumors, and GSCs isolated from the primary tumor. *Black*: normal tissue/cell line; *orange*: TCGA-mesenchymal subtype; *green*: TCGA-proneural; *blue*: TCGA-classical; *purple*: TCGA-neural; *red*: PDX-derived GSCs; *blue*: PDX primary tumor. **b** Stacked *bar graph* showing the proportion of low, medium, and highly modified CpG sites for 5mC, 5hmC, and 5fC/5caC. Cutoffs for defining highly/lowly modified sites are: 5mC, 0.6/0.2; 5hmC: 0.15/0.05; 5fC/5caC: 0.2/0.1. **c** *Ring-bar graph* depicting enrichment of highly modified 5mC (*top*), 5hmC (*middle*), and 5fC/5caC (*bottom*) sites at CpG islands (CGI), shores, shelves, and sea for NSC (outside) and GSC (inside). **d** *Bar graph* illustrating enrichment (normalized to the total number of CpGs associated with each feature, noted in the center panel) of differential modification events between GSC and NSC on average. Hyper-modification events are denoted by positive values, hypo-events by negative values. *Y-axis* enrichment level. *X-axis* genomic features, as shown for 5hmC. **e** Density *scatter plots* showing switching events at the same CpGs between NSC and GSC for 5mC and 5hmC (*left*), 5hmC and 5fC/5caC (*middle*), and 5mC and 5fC/5caC (*right*). *Red dashed line* indicates the cutoff for differential modification of 5mC (0.25), 5hmC (0.05), and 5fC/5caC (0.05). *Histograms* and *boxplots* demonstrate the distribution pattern of each mark. *X-axis* and *Y-axis* indicate levels of modification change for each mark. N number of events in each quadrant

We constructed a phyloepigenetic evolutionary tree based on the 450-K array (measuring 5mC + 5hmC) to obtain an overview of the relationship among GSCs, PDX, and primary tumors. Overall structure of the tree reveals that normal samples (black bars) tend to cluster closely, distinct from most PDX (blue bars)/PDX-derived GSCs (red bars) and an independently derived GSC line H1228 [28], with the four TCGA GBM expression subtypes largely spread in between (proneural-green bars, mesenchymal-yellow, classical-teal, neural-purple) (Fig. 4a). This branching pattern suggests that DNA marks go through substantial reprogramming during pathogenesis. Each primary PDX tumor is positioned adjacent to its corresponding GSC line (Additional file 2: Figure S4A), indicating that DNA methylation in GSCs is highly representative of the primary tumor from which they are derived. As previously reported [13], DNA methylation does not fully characterize the subtypes of primary TCGA GBMs (Additional file 2: Figure S4B). Interestingly, G-CIMP primary GBMs tend to cluster near the GSC population, suggesting G-CIMP tumors might share similar methylation patterns with GSCs.

We mapped DNA epigenetic modifications in greater detail by specifically examining 5mC, 5hmC, and 5fC/5caC, using modified-reduced representation bisulfite sequencing (RRBS) methods, including TET-assist bisulfite sequencing (TAB-RRBS) for 5hmC [29] and *M.SssI*-assisted bisulfite sequencing (MAB-RRBS) for 5fC + 5caC collectively [30]. Modification level for each mark is stratified into low, medium, and high tiers. Migration from the high to the medium tier, rather than complete loss, is observed for 5mC in GSCs compared with NSCs (Fig. 4b, left). In contrast, 5hmC undergoes substantial loss in GSCs, as evidenced by the dominant tier shift from high/medium to low (Fig. 4b, middle). Interestingly, GSCs accumulate medium tier 5fC/5caC sites at the expense of lowly modified CpGs (Fig. 4b, right). We validated our RRBS results at several genomic regions in GSCs and NSCs using DNA immunoprecipitation with antibodies specific for each of the four DNA marks, coupled with real-time PCR (DIP-qPCR). DIP-qPCR results at these loci correlate well with RRBS as shown in the browser views for each DNA mark (Additional file 2: Figure S4C–E). More importantly, DIP-qPCR differentiates 5fC from 5caC at these loci (which our MAB-RRBS protocol does not), unveiling region- and cell line-specific preferences for 5fC and/or 5caC occupancy (Additional file 2: Figure S4C).

The genomic localization of highly-modified CpG sites is modification- and cell type-dependent. In the NSC profile, 5fC/5caC exhibits greater occupancy at promoter TSS1500 regions (5fC/5caC vs 5mC/5hmC: 24% vs 18%) and CpG islands (CGI) (9% vs 4%) (Additional file 2: Figure S4F). However, localization of highly modified sites differs in GSCs, with CGIs becoming more enriched for all three DNA marks at the expense of open sea for 5mC (GSC vs NSC: 19% vs 13%) and 5fC/5caC (28% vs 21%), and shore regions for 5hmC (CGI: 10% vs 12%, shore: 15% vs 13%) (Fig. 4c). We next compared the entire dataset for each mark between cell types and observed global loss of 5mC and 5hmC accompanied by an overall gain of 5fC/5caC in GSC. Specifically, loss of 5mC and 5hmC occurs mainly at intergenic and CpG-poor regions; gain of 5mC occurs more frequently at promoters, gene bodies, and CGIs (Fig. 4d left and middle). In contrast, gain/loss of 5fC/5caC lacks feature-specificity, evidenced by the even distribution across the genome (Fig. 4d right).

We then examined the relationship between each DNA mark, and between NSCs and GSCs. Substantial loss of both 5mC and 5hmC is observed simultaneously in GSC compared to NSC (*n* = 4658), with a small population of sites losing 5mC to 5hmC (*n* = 651) or losing 5hmC to 5mC (*n* = 348) (Fig. 4e, left). While GSCs have globally lower 5hmC levels, a considerable number of these sites meanwhile gain 5fC/5caC (*n* = 1392), followed by 884 sites that lose both 5hmC and 5fC/5caC (Fig. 4e, middle). Interestingly, we observed that hypo-5mC events are largely accompanied by increases in 5fC/5caC (*n* = 3962), followed by 2190 sites that lose both 5mC and 5fC/5caC (Fig. 4e, right). These results taken together suggest that GSCs replace 5mC and 5hmC extensively with 5fC/5caC in the background of a global loss of all DNA marks.

### Distribution of 5hmC and 5fC/5caC reveals novel motif binding factors

**Fig. 5:**
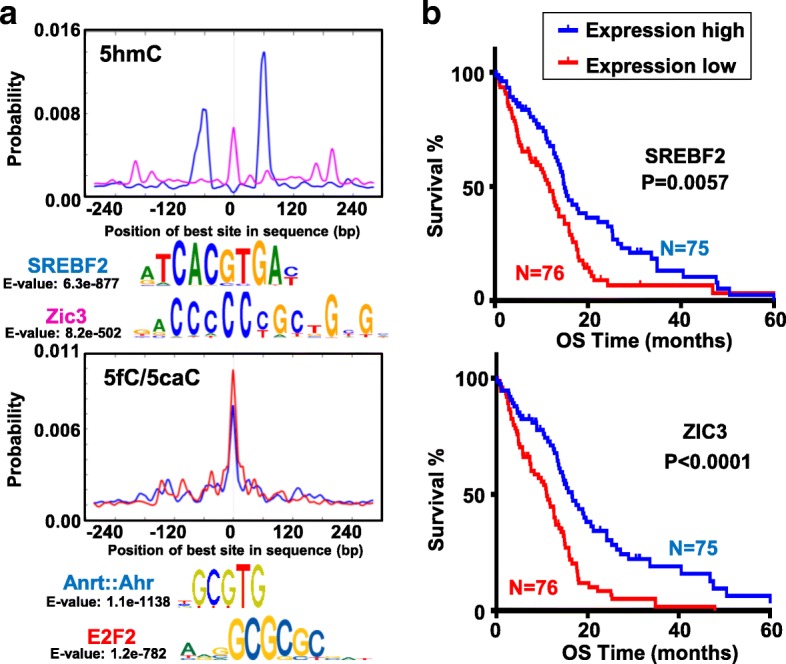
Prediction of novel motif binding factors at 5hmC and 5fC/5caC sites. **a** Predicted motifs and binding probability curves at highly modified 5hmC (*top*) and 5fC/5caC (*bottom*) sites. E-value and motif consensus are shown below each motif probability graph. Binding factors are colored as in the graph. *X-axis* shows the relative distance to the CpG site. *Y-axis* shows the probability value. **b** Five-year survival rate for TCGA GBM patients with high (*blue curve*) and low (*red curve*) expression (separated by median expression value) of SREBF2 (*top*) and ZIC3 (*bottom*). *Y-axis* denotes overall survival time (OS) in months. *X-axis* denotes the survival rate. *P* value indicates significance difference between the two survival curves

We searched for potential binding factors or readers of DNA marks in GSC/NSC by focusing on highly modified 5hmC and 5fC/5caC sites, and sites with differentially modified DNA marks among GSCs and NSCs. We identified the top 50 motif matches for highly modified 5hmC and 5fC/5caC sites (Additional file 1: Table S8). While many hits are shared between the two highly modified DNA mark sites, several motifs are either specific to 5hmC, such as SREBF2 and ZIC3 (Fig. 5a, top), or to 5fC/5caC, such as ANRT/AHR and E2F2 (Fig. 5a, bottom), suggesting that each DNA mark is associated with distinct sets of binding factors/readers. In keeping with the global loss of 5hmC in GSCs, SREBF2 expression is also downregulated in GSCs compared with NSCs and, along with ZIC3, is related to poorer survival as analyzed using GBM data from TCGA (Fig. 5b, Additional file 1: Table S6). Of note, many motifs lacking CG were identified, including those recognized by FOSL2, NR2F1, and TBX15 for 5hmC, and RFX4, NR2F6, and TFAP2B for 5fC/5caC (Additional file 2: Figure S5A) (Additional file 1: Table S8). Rather than being a reader, factors binding to these CpG-free motifs might participate in recruiting or stabilizing cytosine-modifying enzymes/complexes to nearby loci. Predicted binding factors, such as TBX [31] and RFX [32] family members, play important roles in gene regulation during development and cancer progression, and their action may thus be coordinated by these DNA marks.

Binding factors with cell-type specificity could lead to differential functional outcomes between NSC and GSC. We identified 16 GSC-specific and 130 NSC-specific motifs for 5mC, and 23 GSC-specific and 123 NSC-specific motifs for 5hmC (Additional file 1: Table S8). While binding of many of these factors has not previously been shown to be modulated by DNA marks, the expression of several of these factors correlates with levels of their associated DNA mark, and with GBM patient survival. For example, the NKX3–1 binding motif (CG-free) is unique at sites of GSC-specific 5mC and 5hmC, its expression ten times higher in GSCs than in NSCs (FPKM: 1.17 vs 0.12), and it is linked inversely to patient survival (Additional file 2: Figure S5B, top). The binding motif for KLF14 (CG-containing) is enriched only at 5mC-containing sites in NSC, and its transcription is higher in NSC relative to GSCs (Additional file 1: Table S8) and inversely related to patient survival (Additional file 2: Figure S5B, bottom).

### Integration of DNA epigenetic modifications with the transcriptome and enhancer histone marks

**Fig. 6:**
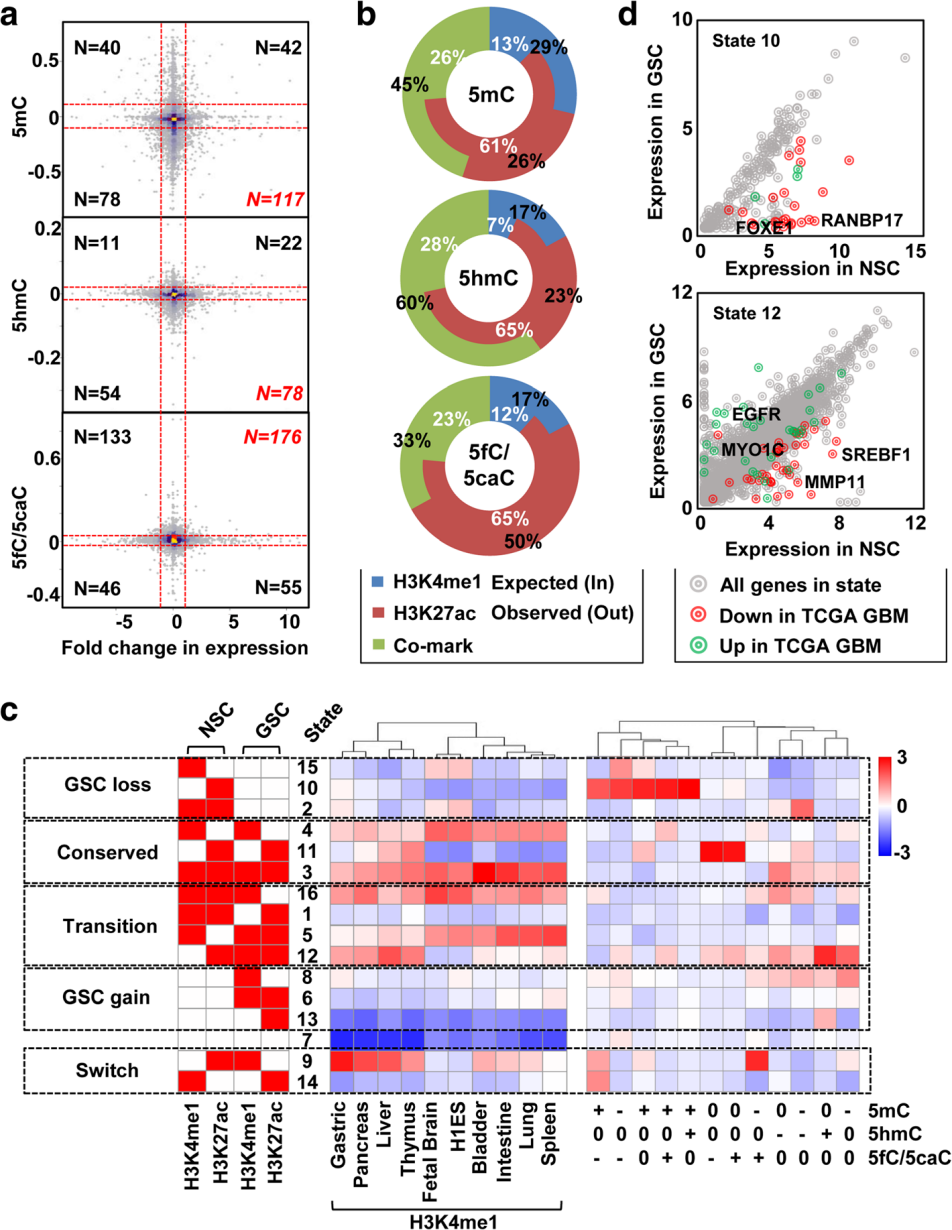
Distinct co-localization of DNA marks with enhancers in NSCs and GSCs. **a** *Scatter plots* showing the correlation of each DNA mark with gene expression changes between GSC and NSC. *X-axis*: average expression change. *Y-axis*: average DNA mark change. *Red dashed line* indicates the cutoff for differential modification of 5mC (0.1), 5hmC (0.025), and 5fC/5caC (0.025), and for expression fold change (> 1 or < − 1). N number of events in each quadrant. **b** *Ring-bar graph* depicting the observed proportion of highly modified 5mC (*top*), 5hmC (*middle*), and 5fC/5caC (*bottom*) (*outer ring*) sites relative to the expected proportional distribution (*inner ring*) at different enhancer histone marked regions. **c** Integrative ChromHMM model of enhancer marks states (*left*, *red color* represents highly enriched), co-localization with tissue-specific enhancers (*middle*), and DNA mark switching events (*right*) in five scenarios representing prominent enhancer differences among GSC and NSC lines (*far left*). Enrichment level for co-localization increases as color migrates from *blue* to *red*. Histone marks, tissues, and DNA mark switching events are labeled at the bottom. DNA mark switching is denoted as follows: 0, no change; +, gain; −, loss. **d** *Scatter plots* showing the expression correlation between GSC and NSC of genes near state 10 (*top*) and state 12 (*bottom*). *Red dots* indicate genes downregulated in TCGA GBM primary tumors compared with normal tissue. *Green dots* are upregulated genes. *X-axis* and *Y-axis* denote average expression as log2 transformed FPKM in NSCs and GSCs, respectively

Given that each DNA mark may have distinct effects on transcription, we determined the relationship between changes in average promoter modification level and expression of the associated gene and observed that upregulation of genes in GSC compared to NSC shows the highest overlap with hypomethylation (*n* = 117), hypohydroxymethylation (*n* = 78), and hyperformylation/carboxylation events (*n* = 176) (Fig. 6a). This is consistent with previous studies, which suggested a repressive role for 5mC and 5hmC at promoter regions [33]; a positive association between 5fC/5caC and transcriptional activity was noted in a recent study [34].

Active enhancers in general are depleted of 5mC but enriched with 5hmC [35]; much less is known about 5fC/5caC at these regions. We examined co-localization of each type of highly modified DNA mark region with enhancer histone marks in NSC, compared with an expected pattern based on chance by referencing global distributions of all CpG sites derived from RRBS datasets. We observe that both 5mC and 5hmC are most enriched at H3K4me1-marked regions (1.9-fold expected for 5mC, 2.2-fold expected for 5hmC), with 5mC preferentially enriched at H3K4me1-marked poised enhancers (5mC vs 5hmC: 29% vs 17%) and 5hmC enriched at H3K4me1 and H3K27ac co-marked active enhancers (5mC vs 5hmC: 45% vs 60%). In contrast, 5fC/5caC, while being consistently enriched at H3K4me1-marked regions, shows a onefold increased enrichment at H3K27ac marked regions relative to that of 5mC and 5hmC (5fC/5caC vs 5mC vs 5hmC: 50% vs 26% vs 23%) (Fig. 6b).

We defined 16 chromatin states by integrating the histone marks from two NSCs and 12 GSCs into a ChromHMM model and then grouped them into five scenarios: GSC loss, NSC-GSC conserved, NSC-GSC transition, GSC gain, and NSC-GSC switch (Fig. 6c, left column). When we examined the relationship between these groups and tissue-specific enhancers defined by H3K4me1, we observe that GSC-lost active enhancers co-localize with a subset of fetal brain-specific enhancers that also exhibit enrichment for other tissue-specific enhancers (states 16, 15, and 2). Meanwhile, GSC-gained enhancers overlap with other tissue-specific enhancers at multiple states, including transitioning state 5 and 12, and switching state 9, in which only NSC possess H3K27ac or H3K4me1, but there is a switch to poised or active enhancer status in GSC. GSC-unique gained enhancer groups (states 8, 6, and 13) show minimal co-localization with other tissue-specific enhancers (Fig. 6c middle). Such enhancer mark-switching patterns suggest that GSCs lack the tissue-specificity of NSCs in part due to loss of tissue-specific footprints established by brain/neural-specific enhancers and gain of other tissue-specific and/or random enhancer marks in the genome.

We demarcated distinct DNA mark switching events between GSCs and NSCs and investigated their co-localization with the most prevalent ChromHMM state changes (Additional file 2: Figure S6A, Additional file 1: Table S9). Several states having a unique combination of enhancer and DNA mark changes were analyzed for their potential impact on nearby genes. A collection of hypermethylation events, regardless of the change in 5hmC and 5fC/5caC, are a major driving force clustering a subgroup of states with exclusive loss of H3K27ac in GSC relative to NSC (state 10). A total of 283 genes functionally relevant to nervous system development and cell adhesion are adjacent to such regions (< 10 kb) (Additional file 1: Table S10, column C), with downregulated expression in GSC (*n* = 41) being the dominant effect (Fig. 6d, top). We compared this result to TCGA GBM data and found 24 genes consistently repressed in primary tumors, including RANBP17, an activator of p21 [36] and an indicator of poor survival (Additional file 2: Figure S6B). An additional 17 downregulated genes are unique to GSC lines, including FOXE1, a developmental transcription factor whose promoter and adjacent enhancer are specifically targeted for DNA hypermethylation in GSC lines (but not bulk tumor based on comparisons with TCGA data). Low FOXE1 expression also correlates with reduced survival of GBM patients (Additional file 2: Figure S6C). Consistent with their hypermethylation-mediated downregulation in GSC lines, ectopic re-expression of RANBP17 and FOXE1 leads to attenuated cell viability in the GSC6 and GSC14 lines that do not express these genes from the endogenous loci (Additional file 2: Figure S6D, E), suggesting they represent growth suppressors targeted via epigenetic mechanisms to promote GSC growth or survival.

Exclusive loss of 5mC and gain of 5hmC in GSC is enriched at states 12 and 8 and correlates with a set of GSC-specific H3K4me1 peaks. Gene deregulation in GSCs is observed near state 12 (loss of 5mC), including 37 upregulation and 105 downregulation events, among which 14 and 36 genes exhibit the same form of deregulation in TCGA GBM samples, respectively (Additional file 1: Table S10, column G). Upregulated genes, such as EGFR and MYO1C, are associated with glioma cell proliferation and migration, whereas downregulated genes such as MMP11 and SREBF1 are related to cell adhesion and lipid metabolism (Fig. 6d, bottom). Regions exclusively gaining 5hmC in state 12 are not linked to altered expression events, whereas 5hmC loss exhibits slightly higher enrichment at states 2, 3, and 16. This lack of enrichment specificity compared to altered 5mC events is consistent with the ubiquitous loss of 5hmC observed in GSCs.

Focusing on switching events among 5mC and 5fC/5caC marks between NSC to GSC reveals a novel correlation; loss of 5mC and gain of 5fC/5caC strongly occupies state 9, a collection of territories that lose H3K27ac to H3K4me1 in GSC (Fig. 6c). A total of 43 genes reside within 10 kb of these regions (Additional file 1: Table S10, column B), including those involved in cancer pathways (e.g. MMP2), angiogenesis (e.g. VEGFA), hypoxia response (e.g. SCAP), and neural development (e.g. KCNQ2), with downregulation of these loci being the dominant transcriptional effect (Additional file 1: Table S3). On the other hand, 5mC gain with 5fC/5caC loss events are enriched at state 14, regions that lose H3K4me1 to H3K27ac (Fig. 6c). Few genes are located near these regions and only a small subset are transcriptionally deregulated in GSC including ANK1, a gene known to maintain cell morphology and promote cancer cell growth [37]. Interestingly, while ANK1 is upregulated in GSCs, it is downregulated overall in primary GBM based on TCGA data (Additional file 2: Figure S6F, top panel), suggesting that GBM intratumoral heterogeneity masks molecular characteristics in GSCs. Using survival information from these 151 patients, we observe that high expression of ANK1 in GBM is associated with poor survival (Additional file 2: Figure S6F, bottom panel).

### Differentiation of neural and glioma stem cells is characterized by distinctive gene regulation events

**Fig. 7:**
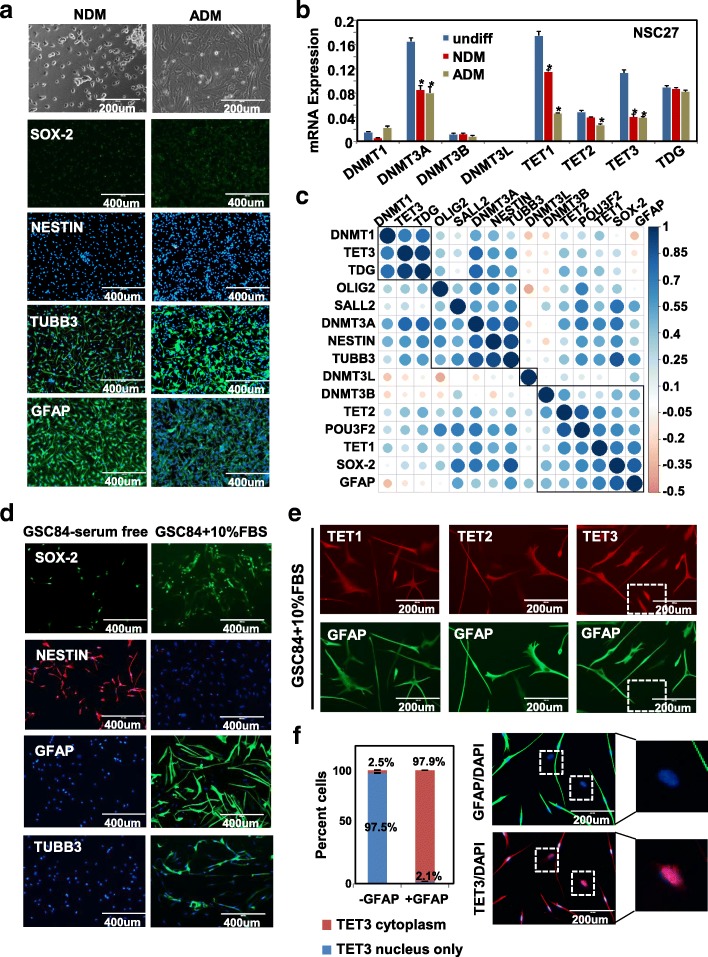
Responses of neural and glioma stem cells to differentiation cues. **a** Representative IF images for NSC27 cultured under neuronal- (NDM) and astrocytic-induction conditions (ADM). Bright field images (*top*) showing cell morphology. Scale bar is indicated. The indicated proteins are stained in *green*. DAPI indicates nuclear staining in *blue*. **b** Expression of DNMTs and TETs before and after differentiation induction in NSC27, expressed as mean ± SEM. *X-axis* shows the gene. *Y-axis* shows the normalized (to DYNLL) mRNA expression. * *P* < 0.05. **c** Correlation of differentiation-induced expression changes among DNA modification machinery genes and stem/lineage markers. *Blue*: positive correlation; *orange*: negative correlation. *Dot size* represents the magnitude of correlation. Genes within the same box are most correlated with each other. **d** IF images for stem and lineage markers in GSC84 cultured under serum-free stem conditions (*left*) and 10% FBS differentiation-inducing conditions (*right*). NESTIN is stained in *red*; SOX-2, GFAP, and TUBB3 are stained in *green*. Scale bar: 400 μm. **e** IF images for TET1, TET2, TET3 (*top*), and GFAP (*bottom*) in 10% FBS conditions in GSC84. The *white dashed box* indicates GFAP expression is absent in cells with nuclear TET3 expression. Scale bar: 200 μm. **f** Stacked *bar graph* showing the percent cells displaying only nuclear TET3 or cytoplasmic TET3 in the absence/presence of GFAP. Representative IF images of GFAP and TET3 are shown at the right. *White dashed box* highlights a region where GFAP expression is absent when TET3 staining is nuclear

Differentiating CSCs is a theoretical approach to eliminate this cell population; however, previous studies suggest that CSCs fail to terminally differentiate [38]. To unveil potential epigenetic mechanisms underlying this process, we induced differentiation in a subset of our NSCs and GSCs. Both NSC27 and NSC30 exhibit an astrocytic phenotype under astrocytic differentiation conditions (ADM) (Additional file 2: Figure S7A). NSC27 exhibits a neuronal phenotype under neuronal differentiation conditions (NDM) (Fig. 7a), consistent with a previous study [39]. In addition, NSC27 shows nearly complete loss of NESTIN, partial loss of nuclear SOX-2 staining, and elevated TUBB3 and GFAP expression (Fig. 7a, Additional file 2: Figure S7A). DNMT3A, TET1, and TET3 expression is significantly reduced in both differentiation conditions and in both NSCs (Fig. 7b, Additional file 2, Figure S7B). These findings show that programming of DNMT and TET expression is highly conserved in NSCs in response to differentiation cues.

In contrast to the phenotypic changes in NSCs, expression programming of GSC stem regulators [40] SOX-2, OLIG2, POU3F2, and SALL2, and lineage marks is heterogeneous under differentiating conditions. While OLIG2 is consistently repressed, responses of other loci vary among GSC lines in response to differentiation cues (Additional file 2: Figure S7C). Induction of GFAP occurs in a subset of GSCs, including GSC12, 39, 84, and 102; TUBB3 activation is observed in GSC6, 10, 12. Protein levels of these markers were quantified by IF with data summarized as the percent positive cells in Additional file 1: Table S1, bottom. DNMT1, DNMT3A, and DNMT3B are downregulated in several GSCs upon differentiation (Additional file 2: Figure S7D). TET2 expression is largely unchanged, whereas TET1, TET3, and TDG respond variably (Additional file 2: Figure S7E). We examined relationships between expression of stem/lineage markers and DNMT/TET expression during GSC differentiation, and observe three clusters of positive correlation, including DNMT3A correlating with OLIG2, SALL2, NESTIN, and TUBB3, and TET1/TET2 correlating with stem markers POU3F2 and SOX2, and lineage marker GFAP. Of note, several inversely correlated gene pairs were observed, including DNMT1, TET3, and TDG, whose expression is anti-correlated with DNMT3B, DNMT3L, and GFAP (Fig. 7c).

In addition to altered expression, dynamic changes in TET subcellular localization occur during GSC differentiation. IF staining for TET expression is consistent with our previous qRT-PCR data (Fig. 1d) and shows that TET1 is highly expressed in NSCs, while TET2 is highly expressed in GSCs (Additional file 2: Figure S7F). Moreover, TET1 and TET3 are more prominently localized to the nucleus in GSCs compared to NSCs. We co-stained for each TET and GFAP in GSC84, a GSC with robust astrocytic lineage differentiation (Fig. 7d). While all three TETs translocate from the nucleus to the cytoplasm and many cells display co-expression of cytoplasmic TET3 and GFAP, ~ 20% of the cells fail to induce GFAP expression under FBS-differentiation conditions (Fig. 7e). These cells express mainly nuclear TET3 and are devoid of GFAP (Fig. 7f, white boxed areas), suggesting that TET3 subcellular redistribution is important for lineage differentiation and the ongoing presence of nuclear TET3 inhibits GFAP expression.

### Atypical reprogramming of the transcriptome and epigenome during glioma stem-cell differentiation

**Fig. 8:**
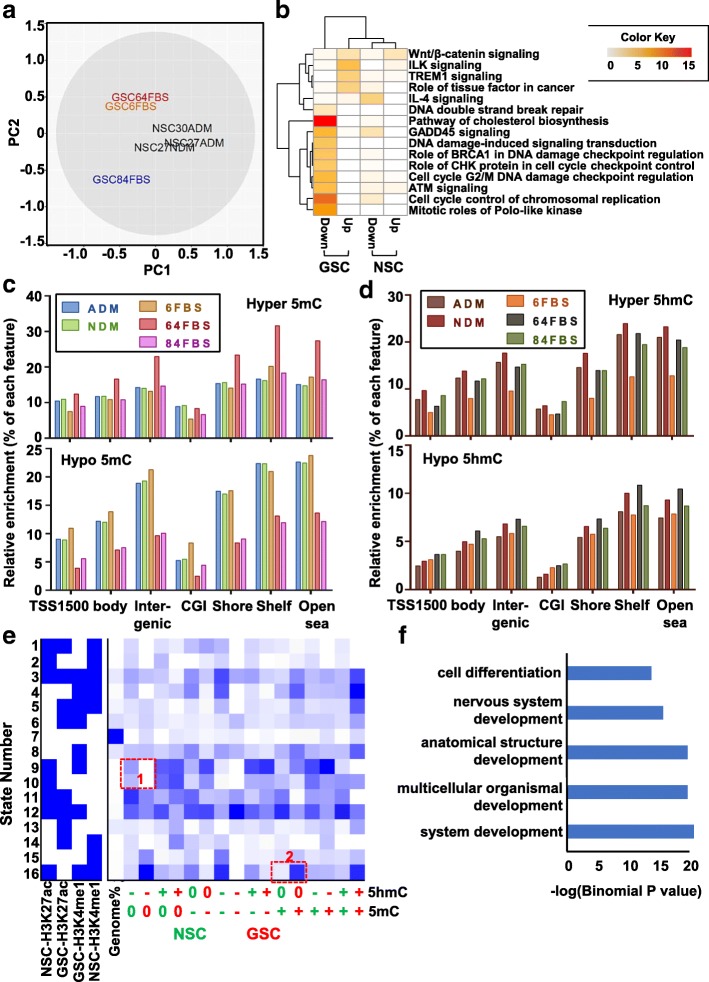
Transcriptome and epigenome reprogramming events during differentiation. **a** Principal component analysis *plot* illustrating differential expression patterns before and after induction of differentiation in each cell line. **b** *Heatmap* showing relative enrichment for the top canonical pathways in the uniquely up- and downregulated genes in each cell type in response to differentiation. Color migrating from *light yellow* to *red* indicates increased enrichment. The *left two columns* show pathway enrichment level for down- and upregulated genes in GSCs. *Right two columns* show the same data for NSCs. **c**, **d** *Bar graphs* depicting the feature enrichment of differential 5mC (**c**) and 5hmC (**d**) events in GSC and NSC, after normalizing to the total number of CpGs associated with the feature and total number of hyper- (positive) or hypo- (negative) events. *X-axis* indicates the genomic feature. *Y-axis* indicates the enrichment level. **e** ChromHMM model illustrating the co-localization of DNA modification changes induced by differentiation related to enhancer marks in the corresponding cell line in its undifferentiated state. *Green* denotes events in NSC, *red* events in GSC. DNA mark switching is denoted as follows: 0, no change; +, gain; −, loss. **f** *Bar graph* showing the biological processes associated with genes gaining methylation during NSC differentiation at state 16 in box 2

DNA modifications and the transcriptome were profiled in GSC6, GSC64, and GSC84, which exhibit efficient “differentiation” and variable TET expression changes, and in NSC27 and NSC30. Principal component analysis shows that transcriptional changes induced by differentiation in NSCs are distinct from those in GSCs, and that GSCs do not respond uniformly (Fig. 8a). More genes are upregulated in GSCs in response to differentiation, whereas up- and downregulation events are comparable in NSC (Additional file 2: Figure S8A, Additional file 1: Table S11). NSC27 and NSC30 share greater similarity in transcription regulation events upon differentiation induction, whereas the GSCs display substantial heterogeneity (Additional file 2: Figure S8B). Uniquely upregulated genes in any one GSC differentiation are associated with developmental disorders, cancer, and organ abnormalities, whereas the downregulation events are linked to the cell cycle, proliferation, and DNA repair (Fig. 8b), suggesting convergence at the pathway level for GSC changes.

Differentiation induction led to extensive DNA epigenetic modification changes in a cell lineage-specific manner. Astrocytic and neuronal induction triggers an almost equal number of hyper- and hypomethylation events in NSC, whereas GSCs under FBS conditions trigger a more variable set of 5mC alterations (Additional file 2: Figure S8C, top). Because 5mC changes are heavily influenced by cell type, samples clustered by cell line rather than differentiation status (Additional file 2: Figure S8D). In contrast, differentiation induced a much greater number of 5hmC gains in both NSCs and GSCs (Additional file 2: Figure S8C, bottom), which in turn led to two distinct clusters formed by differentiation status rather than cell type (Additional file 2: Figure S8E). In addition, GSCs share very few common modification-change events with NSCs during differentiation, again showing that differential regulation of 5mC and 5hmC is highly specific to cell type (Additional file 2: Figure S8F).

Genomic distribution of differentiation-induced 5mC and 5hmC changes did not differ greatly by gene feature or CpG-density. 5mC and 5hmC changes occur more frequently in CpG-sparse and intergenic regions and less commonly in promoters and CpG islands (Fig. 8c, d). We next mapped these sites to the enhancer landscape in the undifferentiated state described earlier and identify distinct localization of DNA marks among GSC and NSC (Fig. 8e). For example, NSCs lose 5hmC at states 9 and 10, while GSCs lose 5hmC at state 16 where NSCs display minimal 5hmC changes. We examined whether differentiation-induced loss of 5hmC in NSC at H3K27ac-marked regions (states 9 and 10) affects expression of nearby genes (Fig. 8e box 1). Downregulation events are indeed identified for such genes including GFRA1, GRIN2D, NKX2–2, and PDGFB, whereas upregulation of CLDN11 occurs in differentiated NSC. This finding suggests that 5hmC loss during differentiation at previously active regions is associated with decreased expression. NKX2–2 expression decreases upon astrocyte induction [41], whereas CLDN11 is upregulated during neural induction [42]. Our data, in keeping with a previous study, show that NKX2–2 is suppressed under astrocytic conditions, while CLDN11 is activated in neuronal conditions. GSCs under differentiation conditions, however, do not show comparable changes, indicating a differentiation defect likely resulting from failure to remodel their epigenome. Hypermethylation occurs specifically during GSC differentiation at state 16 (Fig. 8e, box 2), a collection of regions co-occupied by active enhancers in NSC but poised enhancers in GSC. Genes adjacent to such regions are involved in development and differentiation-related processes (Fig. 8f), thus an increase in 5mC at flanking enhancers may exert an inhibitory effect on differentiation. Indeed, we identified a subset of genes, including MBP (a glial marker) [43], COL18A1 (a neural retina gene) [44], SREBF1 (nerve fatty acid synthesis) [45], and WNT1 (neurogenesis gene) [46] that are upregulated in differentiation-induced NSC27, but not in any GSCs. Taken together, these data suggest that NSCs acquire 5mC/5hmC reprogramming events at regulatory regions of developmental genes during differentiation; this reprogramming process, however, is less specific in GSCs, which may account for their more varied response to differentiation cues and the ability of “differentiated” derivatives to continue to proliferate and contribute to the bulk tumor mass.

### Co-expression analysis and differential promoter DNA modification levels link TET2 and TET3 to the DNA damage response

**Fig. 9:**
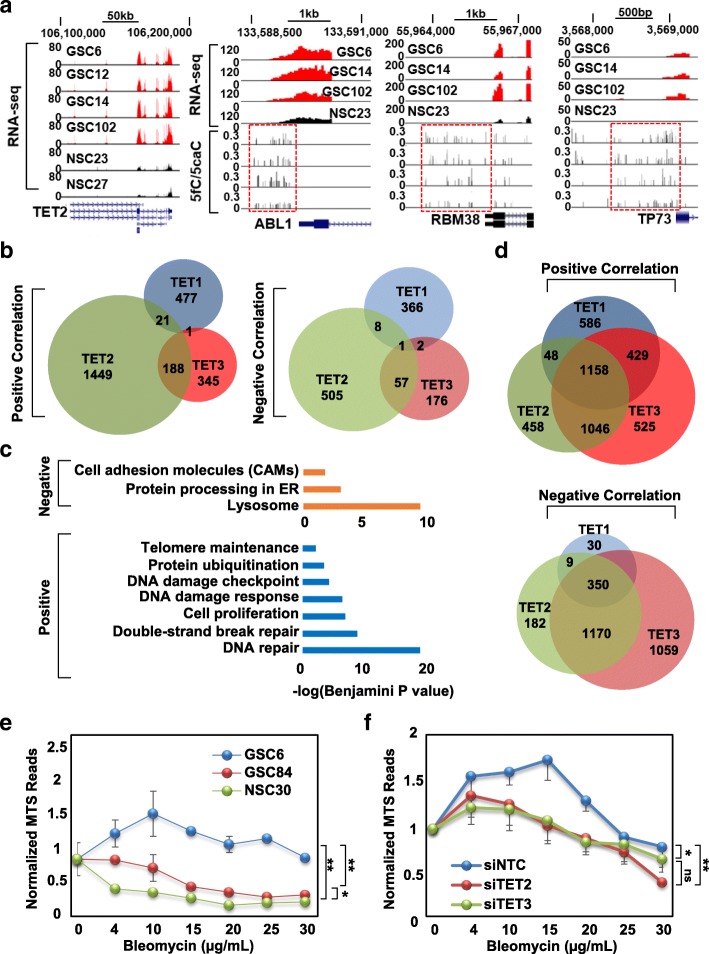
Co-expression of the TETs with DNA damage response genes and the functional impact of TET knockdown on cell viability under DNA damage conditions. **a** Transcript abundance for TET2 (*far left*) with the top four browser tracks displaying RNA-seq signals for GSC6, GSC12, GSC14, and GSC102, and the bottom two tracks showing RNA-seq data for NSC23 and NSC27. The three panels to the *right* show transcript levels (top four tracks, from RNA-seq) and 5fC/5caC levels (based on MAB-seq data, bottom four tracks) at the promoters of ABL1, RBM38, and TP73 in the indicated GSC and NSC lines. Order of cell lines is the same for RNA-seq data (top four tracks) and MAB-seq data (bottom four tracks). The genomic coordinates are denoted on the top of the track. Scale bar is indicated. *Y-axis* denotes the enrichment level represented by the raw reads of RNA-seq and ratio of modified reads to total reads for 5fC/5caC level. *Red dashed box* indicates the differentially modified promoter regions. **b** *Venn diagrams* showing the overlap of genes correlating with expression of each TET from the RNA-seq datasets. *Left*: positively correlated; *right*: negatively correlated. **c** *Bar graphs* showing ontology analysis of TET2 positively and negatively correlated genes. **d** *Venn diagrams* showing the overlap of genes correlating with expression of each TET in the TCGA GBM RNA-seq dataset. *Top*: positively correlated; *bottom*: negatively correlated. **e** *Line graph* showing the response of GSC6, GSC84, and NSC30 to bleomycin at different concentrations after 72 h. Data are normalized to the absorbance at 470 nm of vehicle and expressed as the mean ± SEM. Three replicates are performed for each drug concentration. Significance difference is assessed between each GSC and NSC30. *P < 0.05. **P < 0.01. **f** *Line graph* showing the viability of GSC6 transfected with each of the indicated small interfering RNAs at different concentrations of bleomycin and viability expressed as in part E. Significance difference is assessed between siNTC, siTET2, and siTET3. *P < 0.05. **P < 0.01. ns not significant

Our current analysis reveals that although TET expression is heterogeneous in GSCs, TET2 expression is associated with a significantly higher level of 5fC/5caC and lower level of 5mC (Fig. 1f), whereas TET3 may inhibit GSC differentiation (Fig. 7e, f). Given these observations, we examined whether a larger gene set displays consistent relationships with TET expression by linking expression of each TET to promoter DNA marks and gene expression on a genome-wide level. Gene sets with TET-correlated promoter modifications are highly specific for each TET and each DNA mark (Additional file 2: Figure S9A). Compared to TET1 and TET3, TET2 levels positively correlate with 5mC and negatively correlate with 5hmC and 5fC/5caC at a larger set of promoters. TET1 and TET3 levels, on the other hand, positively correlate with 5hmC and 5fC/5caC and negatively correlate with 5mC at more gene promoters (Additional file 2: Figure S9A). Ontology analysis for these genes demonstrates that TET2 is specifically negatively correlated with 5mC at promoters of genes related to Hippo signaling, such as SMAD3 and LIMD1 (Additional file 1: Table S12, Additional file 2: Figure S9B), and is positively correlated with promoter 5fC/5caC levels at DNA damage repair genes (e.g. ABL1, RBM38, and TP73) (Fig. 9a), cell-cycle genes (e.g. CCNE1), choline metabolism genes (e.g. RALGDS), and PI3K-AKT signaling genes (e.g. LPAR2) (Additional file 2: Figure S9C). TET3 postively correlated genes are specifically enriched in Hippo and neurotrophin signaling pathways, and organ morphogenesis, which contribute to developmental processes; all three TETs are linked to promoter 5fC/5caC levels at genes related to cancer pathways and stem-cell pluripotency (Additional file 1: Table S13).

A co-expression analysis approach was taken to identify genes potentially regulated by each TET in both our GSC dataset and the TCGA GBM dataset. Using our RNA-seq data, a total of 878, 2229, and 771 genes are linked to TET1, TET2, and TET3 expression, respectively (Additional file 1: Table S14). Based on this analysis each TET relates to a unique set of genes, but TET2 and TET3 share more common genes while TET1 is linked to a distinct group (Fig. 9b). Genes co-expressed with TET1 or TET3 are not significantly enriched for specific pathways (although several DNA damage repair and cell-cycle-regulating genes are in the TET3 list but do not reach the cutoff for statistical significance [not shown]). Genes positively correlating with TET2 expression are, however, strongly enriched in cellular processes including cell-cycle regulation, DNA damage repair, and telomere maintenance; TET2 negatively correlates with genes associated with the lysosome and cell adhesion processes (Fig. 9c, Additional file 1: Table S15). Next, we examined whether similar co-expression patterns (downloaded from cBioPortal) exist in bulk tumor and from a larger independent dataset using TCGA GBM data. Unlike GSCs, the TETs share a greater number of commonly co-expressed genes in primary GBM (Fig. 9d). Again, genes involved in lysosome regulation, metabolic pathways, and oxidative phosphorylation are negatively correlated with TET expression in general, while biological processes including DNA damage repair, cell proliferation, and pluripotency of stem cells are enriched for positively correlated gene sets for all three TETs (Additional file 1: Table S16). These co-expression patterns based on primary tumor data are well-preserved in GSCs for TET2, but not for TET1 and TET3, strongly suggesting that TET2 is involved in regulating cell-cycle control and DNA damage repair, functions that have been previously linked to TET2 and 5hmC [47].

The links we identified between TET2 and expression of genes regulating aspects of DNA repair motivated us to examine this link functionally. We monitored the viability of a representative GSC line with high TET2 expression (GSC6), a low TET2-expressing line (GSC84), and NSC30 (TET2 levels comparable to GSC84) after exposure to bleomycin, a chemotherapeutic agent that induces DNA double-strand breaks and mimics the effects of ionizing radiation [48]. NSC30 exhibits reduced proliferation at low bleomycin concentration. GSC84, whose TET2 expression is close to that of NSC30, also shows significantly attenuated proliferation at low bleomycin concentration. Proliferation of GSC6 (highest TET2 levels), however, is least affected by bleomycin (Fig. 9e). To directly investigate the contribution of TET2 to the response of GSC6 toward bleomycin-induced DNA damage, we performed the same assay under TET2- and TET3-small interfering RNA (siRNA) knockdown conditions (Additional file 2: Figure S9D, TET3 is included because it too exhibits a modest expression correlation with DNA repair genes). Results show that proliferation of TET2-depleted GSC6 is significantly reduced by bleomycin treatment relative to a no-target control siRNA transfection (*P* value = 0.009), while TET3 depletion also decreases cell proliferation, but to a lesser extent (*P* value = 0.015) (Fig. 9f). That suppression of TET2 and TET3 in GSC6 leads to attenuated proliferation under conditions of DNA damage suggests that they play a direct role in modulating the DNA damage response process and may contribute to the chemo−/radio-resistance of glioma stem cells.

### Non-CpG methylation exhibits unique patterns in GSCs and in response to differentiation

**Fig. 10:**
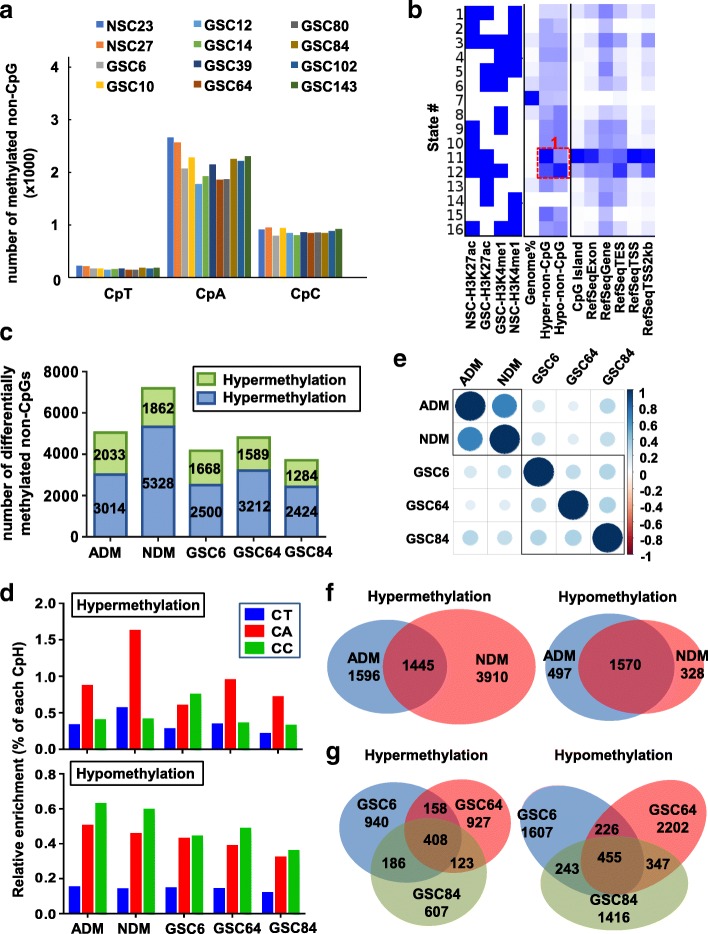
Distinct non-CpG methylation patterns in GSC and NSC. **a** *Bar graph* demonstrating the total number of highly modified CpT, CpA, and CpC sites in each cell line. **b** ChromHMM model illustrating the co-localization of non-CpG methylation changes among NSC and GSC lines. Enrichment level for co-localization increases as color migrates from *light blue* to *dark blue*. A *heatmap* depicting histone marks and states formed by different combinations is shown at the *left*. A region of interest discussed in the results section is marked with a *red box*. **c** Stacked *bar graph* showing gained and lost non-CpG methylation events, noted by the numbers in each bar, in NSCs and GSCs in response to differentiation cues. *Blue*: gain; *green*: loss. Cell line types and the differentiation condition are labeled on the *X-axis*. ADM astrocytic induction of NSC27, NDM neuronal induction of NSC27. GSC6/64/84: FBS induction. **d** *Bar graph* illustrating enrichment of differential non-CpG methylation events induced by differentiation of NSC and GSC lines. *Top*: hypermethylation events. *Bottom*: hypomethylation events. *X-axis*: genomic feature. *Y-axis*: percent enrichment by normalizing to the total number of non-CpGs associated with each feature. **e** Correlation of differentiation-induced non-CpG methylation changes in NSC and GSC. *Blue*: positive correlation; *orange*: negative correlation. *Dot size* represents the magnitude of correlation. Samples within the same box are most correlated with each other. **f** *Venn diagrams* illustrating the overlap of hyper- (*top*) and hypo- (*bottom*) non-CpG methylation events between astrocytic and neuronal differentiation induction conditions for NSC27. **g** *Venn diagrams* illustrating the overlap of hyper- (*left*) and hypo- (*right*) non-CpG methylation events in FBS-induced differentiation conditions for GSC6, GSC64, and GSC84

Since non-CpG methylation undergoes dynamic modification during differentiation of neural progenitor cells and becomes highly enriched in neurons [49], we investigated non-CpG methylation levels in our dataset and compared their patterns between NSC and GSC before and after differentiation. Our detection method yielded ~ 3 M CpA, ~ 1.1 M CpC, and ~ 1 M CpT sites (Additional file 2: Figure S10A, left). Throughout the genome, < 4000 non-CpG sites are highly modified (methylation ratio > 0.4) in NSC, occurring at CpA (70%) > CpC (24%) > CpT (6%, Additional file 2: Figure S10A, right). A reduction of highly modified sites (ratio > 0.4) in GSCs is observed in each non-CpG context (Fig. 10a). A total of 535 non-CpG sites are hypermethylated and 1694 sites hypomethylated in GSCs compared with NSCs (cutoff 0.2). Like the genomic distribution of differential CpG methylation, non-CpG hypermethylation occurs more frequently in TSS1500 regions and CpG islands, whereas hypomethylation is more frequent at intergenic regions (Fig. 4d, Additional file 2: Figure S10B). We aligned our histone modification data with differentially modified non-CpG methylation sites using ChromHMM and identified 12 genes with hypermethylated promoter non-CpG sites in state 11 (Fig. 10b, box 1). Although these promoters are occupied by H3K27ac in GSC, their expression overall is downregulated in GSC (Additional file 2: Figure S10C), including DLX1, an interneuron developmental transcription factor [50], and SEZ6L2, a transmembrane receptor regulating neurite outgrowth [51]. CpG methylation at these promoters does not differ between GSCs and NSCs, but non-CpG methylation is significantly higher in GSC (Additional file 2: Figure S10D), suggesting highly methylated non-CpG sites at select promoters dictate a transcriptionally repressive state.

Upon differentiation there is an overall increase in non-CpG methylation (delta > 0.1), with neuronal induction in NSCs resulting in the greatest number of hypermethylated non-CpG sites (Fig. 10c). Meanwhile, hypermethylation occurs more frequently within CpA, while the majority of hypomethylation events occur in CpC and CpA context (Fig. 10d). Correlation analysis reveals a similar pattern to that of CpG methylation, in which differentiation induced non-CpG methylation changes are highly specific to cell type and vary by genomic feature (Fig. 10e, Additional file 2: Figure S10E). Differentiation-induced non-CpG hypermethylation is also lineage-specific as neuronal differentiation triggers a substantial and unique set of gains (*n* = 3910) in non-CpG methylation in NSC (Fig. 10f left), while hypomethylation events are more common between two lineages (Fig. 10f right). In contrast, differential non-CpG methylation is largely unique to each GSC, especially for hypomethylation events (Fig. 10g), suggesting a distinct differentiation path between NSC and GSC.

## Discussion

Glioma stem cells exist as a minor subpopulation within the bulk tumor, but actively contribute to overall radiation and chemo-resistance, and tumor recurrence [6]. We referenced fetal brain-derived neural stem cells, since they are a possible cell of origin for GSCs, and profiled genome-wide DNA epigenetic modifications, enhancer marks, and transcriptomes in multiple PDX-derived GSCs. We performed an integrated analysis and compared epigenetic signatures before and after differentiation in each cell type to pinpoint cancer stem-cell-specific epigenetic abnormalities. Our data reveal several novel findings: (1) deregulation of TET expression correlates with global loss of 5mC and 5hmC, and gain of 5fC/5caC in GSCs, switching patterns of which display a distinct co-localization with enhancer mark changes; (2) despite heterogeneity within GSCs at both the transcriptional and enhancer levels, these cells exhibit substantial overlap in the deregulated genes/pathways that are highly associated with the differentially modified enhancer landscape between GSC and NSC; (3) TET expression responds differently during differentiation in GSCs compared to NSCs, with subcellular localization of TET3 associated with differentiation proficiency; (4) differentiation-induced reprogramming of 5mC and 5hmC localizes to distinct enhancer regions and contributes to the regulation of developmental genes; (5) integrative analysis identifies novel epigenetically deregulated loci relevant to GBM patient survival and GSC growth; and (6) high-level TET2 expression in GSCs contributes to their chemo-resistance, likely through modulation of the DNA damage response and repair system.

A primary goal of our study is to identify unique epigenomic features and associated gene networks in glioma stem cells that could be targeted clinically without harming neighboring normal cells. Global hypo-5mC/5hmC and promoter-specific hyper-5mC events are frequent in cancer. A potential division of labor exists within the TET protein family, with TET2 more efficient at generating 5fC and 5caC [16] and playing a greater role at enhancers [52]. For the first time, we show a global elevation in 5fC/5caC levels in GSCs, which is potentially attributable to elevated TET2 expression. Further support for this notion comes from our finding that TET2 expression strongly correlates with 5fC/5caC quantity and higher levels of TET2 transcription and 5fC/5caC in GSCs. Through global correlation analysis, we also discovered that TET2 expression is associated with a more robust response to DNA damage in GSCs. Previous studies demonstrated that GSCs exhibit enhanced ability to repair DNA damage through activation of checkpoint proteins such as ATM, RAD17, and CHK1/2 after exposure to radiation [53]. This survival advantage is eliminated by inhibition of CHK1/2; however, normal stem cells in this environment may suffer by acquiring undesired mutations due to the low-selectivity of CHK1/2 inhibition. Our results reveal that GSCs express DNA repair genes at a higher level than NSCs, which may contribute to their resistance to radiation- or chemotherapy-induced DNA damage. Meanwhile, because TET2 expression is higher in GSCs than NSCs, and TET2 expression linearly correlates with expression of repair genes, TET2 may modulate expression of this group of genes and thus serve as a potential therapeutic target. Given that TET2 is lowly expressed in neural stem cells and is further repressed during NSC differentiation, TET2 inhibition might have less impact on NSCs than on GSCs. In support of this, depletion of TET2 using siRNA knockdown sensitizes a GSC line to bleomycin treatment, suggesting that TET2 indeed functions to promote DNA repair or survival after DNA damage. A recent study showed that 5hmC localizes to DNA damage foci and facilitates DNA repair, a process requiring TET2 expression [47]. Our study suggests that TET2 and 5fC/5caC might be specifically involved in this process in GSCs. In addition, TET2 may also be responsible for generating promoter 5fC/5caC at cell cycle and DNA damage response-related genes whose expression is higher in GSC than NSC and positively correlated with TET2 levels, suggesting a possible mechanism through which TET2 modulates expression of these loci.

Another potential approach to eliminate or repress GSCs is to trigger an effective and lasting differentiation process. Previous studies revealed that *in vitro* induction of differentiation by growth factor withdrawal and addition of serum/bone morphogenetic proteins (BMPs) only triggers a process of pseudo-differentiation. These “differentiated” cells are vulnerable to cell-cycle re-entry and do not properly remodel their DNA methylation patterns [38]. Based on our study, we observe that the DNA methylation/demethylation machineries are not regulated in the same way in GSCs compared to NSCs, even though stem-cell markers, cell-cycle genes, and DNA repair pathways are inhibited upon differentiation induction as expected. This phenomenon might be directly attributable to the distinctive patterns of methylation/hydroxymethylation reprogramming in NSC and GSC, in which gain and loss of region-specific epigenetic marks is required for proper and unidirectional differentiation. More importantly, in addition to the well-established role of the TETs in brain development, we observed a specific abnormality in TET3 expression in GSC upon differentiation. Although TET expression in GSCs did not decrease in response to differentiation induction, the localization pattern of TET shifted from the nucleus to the cytoplasm and was accompanied by increased GFAP expression as shown in GSC84, one of the most efficiently differentiated GSCs. An unbiased correlation analysis revealed that TET3 expression is inversely correlated with GFAP expression. IF analysis indeed showed that when TET3 fails to translocate to the cytoplasm, GFAP expression is also attenuated. This irregular TET expression change and/or subcellular distribution under differentiation conditions might contribute to the inability of GSCs to effectively remodel their epigenome from a stem to a lineage committed and differentiated status, and thus account in part for the pseudo-differentiation phenotype.

One possible origin for GSCs is through transformation of neural stem/progenitor cells. As such, GSCs may possess attributes that contribute to normal stem-cell maintenance, while also possessing unique epigenetic characteristics associated with or driving their transformation. A previous study showed that neural progenitors committed to a specific lineage give rise to distinct glioma subtypes, even in response to the same driver mutation [54]. Given that malignant transformation of neural progenitors may occur at different developmental stages (lineage restricted or not) and originate from different regions of the brain, it is possible that GSCs derived from this process preserve distinct epigenetic signatures of their ancestry, which in part contributes to heterogeneity within the GSC population. An earlier study reported that reprogramming of the DNA methylome in GSCs to a non-neural lineage reactivates expression of tumor suppressors and inhibits tumorigenesis [55]. This finding suggests that even though GSCs originate through different mechanisms and/or cell types reflected by their genetic and epigenetic heterogeneity, these events collectively play a critical role in maintaining their cancer stem-cell identity and likely converge on common biological characteristics, as shown in our results and those of others [56]. Meanwhile, we observe heterogeneity among GSCs at multiple levels, including transcription, DNA modifications, and enhancer marks. This heterogeneity is also partially reflected at the level of TET expression among the GSCs. Recently, Jin *et al.* reported that heterogeneity among GSCs gives rise to distinct sensitivities to different epigenetic targeting mechanism [57]. Our observations related to TET2 and its relationship to DNA damage repair also support this conclusion, in that high TET2-expressing GSCs might be clinically more resistant to drug therapy and that a treatment targeting TET2 is likely to be effective in GSCs expressing TET2 at high level, instead of all GSCs, when combined with other cancer therapies.

## Conclusions

The present study has identified several novel epigenetically and transcriptionally convergent events, pinpointed key relationships between the TET machinery and unique epigenomic features of GSCs, and revealed distinctive GSC-specific patterns of TET expression during differentiation. These GSC-specific features likely bestow this cell population with unique molecular profiles and epigenetic properties, which represent potential therapeutic targets for inhibiting or eliminating the GSC population.

## Methods

### Glioma and neural stem-cell culture

Tumor tissue from a well-characterized panel of GBM patient-derived xenografts (PDX) developed as part of the Mayo Clinic Brain SPORE, was mechanically dissociated and selected for the stem-cell population in stem-cell culture medium (ThermoFisher, StemPro NSC SFM A1050901) containing L-glutamine and penicillin-streptomycin using laminin (#L2020) coated flasks to reduce culture condition-introduced heterogeneity [15, 58, 59]. Fetal brain-derived neural stem cells, NSC23, NSC27, and NSC30, were obtained from Dr. Philip H. Schwartz at the Children’s Hospital at Orange County (CHOC) [60]. Neural stem cells were expanded on Matrigel (Corning 356,230) or fibronectin-coated 6-well plates in stem-cell growth medium (GM) containing DMEM/F-12 plus Glutamax (Life Technologies 10,565–018), BIT9500 serum substitute (1×, Stem Cell Technologies 09500), heparin (20 μg/mL Sigma H3149), primocin (1×, InvivoGen ant-pm-1), human bFGF (20 ng/mL, Stemgent 03–0002), and human EGF (20 ng/mL, R&D Biosystems 236-EG) [28]. Low passage cultures of cells (< 5 and typically < 3 passages) were used for all experiments and deep sequencing.

### Differentiation of glioma and neural stem cells

Cells were seeded at low density (~ 0.2–0.5 × 10^6 per well of 6-well plate). Culture vessels were coated with poly-l-lysine followed by laminin to promote cell adherence. For neuronal lineage differentiation, growth factors bFGF and EGF were withdrawn for GSCs; neural differentiation medium containing Neurobasal medium (ThermoFisher, 21,103), B-27 supplement (ThermoFisher, 17,504), GlutaMax-I supplement (ThermoFisher, 35,050), dibutyryl cAMP (0.5 mM added on day 7 for three days) and primocin was used for NSCs. For astrocytic lineage differentiation, 10% FBS was added to the culture medium for GSCs; astrocyte differentiation medium containing DMEM (ThermoFisher, 11,995), N-2 supplement (ThermoFisher, 17,502), GlutaMax-I supplement, 10% FBS, and primocin was used for NSCs. Cells were incubated in differentiation media for 20–30 days.

### Immunofluorescence, immunohistochemistry, and imaging

For IF, cells were seeded onto a fibronectin- or laminin-coated surface and fixed with 4% paraformaldehyde (Electron Microscopy Sciences, 15,710) and permeabilized with 0.1% Triton X-100, followed by 5% goat serum blocking before primary antibody incubation overnight at 4 °C. IHC staining for FFPE tissues from PDX (5-μm thickness) was performed at the Mayo Clinic Pathology Research Core. Slides were retrieved using Epitope Retrieval 2 (EDTA; Leica), incubated in Rodent Block M (Biocare) for 30 min, followed by 15 min incubation of the primary antibody diluted in Background Reducing Diluent (Dako). DAB and DAB buffer (1:19 mixture) and Schmidt hematoxylin were applied for visualization (Bond Polymer Refine Detection System). The dilution factor for each antibody is as follows, anti-Nestin (AB92391,1:200), anti-SOX2 (MAB2018,1:50), anti-GFAP (AB4648, 1:100), anti-CD44 (HPA005785, 1:100), anti-Tuj1 (801,201, 1:100), anti-Galactocerebroside (MAB342, 1:100), anti-5mC (Calbiochem; Clone 16233D3, 1: 200), and anti-5hmC (Active Motif, 39,769, 1:1500). Imaging was performed using an EVOS FL cell imaging system (Life Technologies). Positive cells were counted from at least three fields (each containing > 50 cells). The “stemness” was ranked based on the proportion of cells staining positive for stem and lineage markers.

### Ectopic expression and knockdown, bleomycin treatment, and cell viability measurements

An infection complex composed of 20 μg of FOXE1 (VB170518-1071veg) or RANBP17 (VB170518-1058gjg) expression vector (Cyagen Biosciences Inc.), 10 μg packaging plasmid psPAX2, and 5 μg envelope plasmid pMD2.G were combined with 70 μL X-tremeGENE HP DNA Transfection Reagent in 2 mL opti-MEM media, which was then applied to HEK293T cells. Virus was collected at day 4 and day 5 from the supernatant and filtered through a 0.45-μm filter. Virus was concentrated at 26,000 rpm for 2 h at 4 °C then resuspended in 1 mL serum-free DMEM. For transduction, 2–3 × 10^5^ cells per well of GSC6 and GSC14 were seeded into a 6-well plate, followed by addition of 50–100 μL concentrated virus and 6.5 μL polybrene (4 μg/mL) for 8–12 h. Fresh GSC media was then added and cells were harvested at the indicated timepoints for viability assay (Promega CellTiter 96 Cell Proliferation Assay), RNA isolation, and real-time PCR. For knockdown experiments, GSC6 cells were seeded at a density of 0.5 × 10^6 per well in 6-well plates. Twelve hours after seeding, cells were transfected with siRNA no-target control (siNTC) and siTET2/TET3 (GE Dharmacon, SMARTpool) at 50 nM and 10 uL PepMute™ siRNA Transfection Reagent (SignaGen Laboratories) per well. After 24 h, cells were transfected again with the same reagents at the same concentration to increase knockdown efficiency. For assessing the effect of siRNA knockdown, cells were collected for RNA isolation and real-time PCR after three days. For bleomycin treatment, transfected cells were re-seeded into 96-well plates at a density of 5000 cells per well and supplied with different concentrations of bleomycin for three days. An MTS assay (Promega, Madison, WI, USA) was then performed to assess viability.

### RNA isolation, real-time PCR, and transcriptomic profiling by RNA-seq

RNA was extracted using Trizol reagent following the manufacturer’s protocol (Life Technologies). Reverse transcription and real-time PCR were performed as described [61]. MiRNA was extracted with the mirVana miRNA Isolation Kit (Life Technologies #AM1561), and reverse transcribed and detected by real-time PCR using the miScript PCR system (Qiagen). Primer sequences are in Additional file 1: Table S17. Quality control of mRNA, library preparation, and paired-end sequencing were conducted at the University of Minnesota Genomics Center using an Illumina HiSeq 2500. Approximately 30 million reads were obtained for each sample (Additional file 1: Table S18). Raw reads were mapped to the human genome hg19 and transcripts assembled using TopHat. Quantification and pair-wise comparison of gene expression was conducted through Cufflinks and Cuffdiff packages, respectively. Fragments per kilobase of transcript per million mapped reads (FPKM) was generated to represent transcriptional level and for generating figures. A total of 57,883 unique transcripts were mapped, including 1416 miRNAs. Differentially expressed genes were defined using both fold-change and statistical q-value generated by the Cuffdiff package (false discovery rate [FDR]) (cutoff > 1 or < − 1).

### Differential and co-expression analysis using RNA-seq data

To identify gene sets exhibiting co-expression with each TET we required a Pearson correlation *P* < 0.05 and log2-transformed expression fold change between the highest and lowest sample > 0.5. For TCGA GBM data, the co-expression gene list was acquired from cBioPortal using a Pearson correlation > 0.3 or < − 0.3. MiRNA targeting and network analysis was performed with miRDB (http://www.mirdb.org/miRDB/) and miRNet (http://www.mirnet.ca/) databases. Relationships between gene expression and patient survival were determined by the survival *P* value, calculated with a R package “Survival” under the “survdiff” function, whereas the odds ratio and its *P* value were calculated under the “coxph” function. Only genes with both *P* < 0.05 and expressed in more than half of all patient samples (*n* > 75) were considered to have prognostic value.

### Enhancer mapping using chromatin immunoprecipitation sequencing (ChIP-seq)

In brief, 10 million cells were fixed with 1% formaldehyde. Fixed cells were pelleted and lysed with lysis buffer. Cells were subjected to MNase digestion (NEB, M0247S), followed by sonication using a Diagenode Twin sonicator. Chromatin input was taken from the supernatant and quantified with a Qubit 3.0 fluorimeter. Antibodies recognizing H3K4me1 (AB8895) and H3K27ac (AB4729) were added to immunoprecipitate enhancer-associated DNA fragments using GE magnetic sepharose protein G beads (#28–9670-70). The beads were washed with high-salt, low-salt, and Tris/LiCl buffer. The binding complex was eluted from the beads and reverse cross-linked. DNA was then treated with RNase and proteinase K and purified using a Qiagen mini-elute kit. Paired-end sequencing was performed on an Illumina HiSeq 2500 at the Medical Genome Facility at Mayo Clinic using the ThruPLEX DNA-seq kit (Rubicon Genomics #R400428). The coverage report can be found in Additional file 1: Table S19.

### Stratifying cell type and tissue specific enhancers with ChIP-seq data

ChIP-seq peak calling was performed with the SICER package at the default settings and corrected for input. Differential enrichment was identified using SICER-diff at FDR < 0.01. Unique and common differentially enriched peaks across all glioma stem-cell lines vs neural stem-cell lines were identified with BEDOPS v2.4.20. The R package “ChIPseeker” was used to annotate ChIP peaks with general genomic features and compare feature distribution across different subsets of peaks. Tag density plots of transcript abundance centered at enhancer marks was plotted with deepTools2 software. Genes near enhancer peaks were mapped using Genomic Regions Enrichment of Annotations Tool (GREAT). Human tissue-specific enhancer regions marked with H3K4me1 were extracted from ten different tissue types using BEDOPS v2.4.20, including H1ES (GSM537679), fetal brain (GSM806934), gastric (GSM910574), pancreas (GSM910576), small intestine (GSM956019), lung (GSM1059443), thymus (GSM1059446), bladder (GSM1059450), liver (GSM1059451), and spleen (GSM1120351). Peaks called by SICER with FDR < 0.01 and enrichment score ≥ 30 were used for selecting regions uniquely marked in each tissue type. On average, 60,000 peaks from each tissue were assessed for overlap. When at least 60% of a single peak from one tissue overlapped with another, they were considered shared between those tissues. A total of 32,640 unique H3K4me1 peak regions were identified as brain-specific enhancers.

### Genomic DNA isolation, quantification by mass spectrometry, and DNA mark profiling by (modified) reduced representation bisulfite sequencing (RRBS)

Genomic DNA was isolated using a serial phenol: chloroform extraction and was quantified for total cellular levels of 5mC, 5hmC, 5fC, and 5caC by LC-MS/MS as described [16]. DNA from human ES, neural stem cells, human fetal brain, normal astrocytes, oligodendrocyte precursor cells, normal white/Gy matter-regions, and PDX-derived GSCs was assayed for methylation using the Infinium HumanMethylation450K BeadChip. DNA methylation of the primary PDX tumors was assayed on the Infinium MethylationEPIC BeadChip (only probes overlapping with the 450 K beadchip were used). For RRBS, genomic DNA was subjected to *Msp*I digestion, size selection, and library preparation following a standard RRBS protocol. Paired-end sequencing was conducted on an Illumina HiSeq 2000 at the Medical Genome Facility at Mayo Clinic Rochester. Raw sequencing reads were aligned to human genome hg19 and analyzed through a Streamlined Analysis and Annotation Pipeline (SAAP-RRBS) [62]. A methylation score was assigned based on the ratio of total methylated reads/ total sequenced reads. Only CpGs with > 10X coverage were used for downstream analysis. For TET-assist bisulfite sequencing (TAB-RRBS), *Msp*I digested DNA was first treated with β-glucosyltransferase to protect all 5hmC sites from the subsequent Tet1-based oxidation reaction following the company’s standard protocol (WiseGene kit #K001). Spike-in 5hmC and 5mC controls were used for evaluating the efficiency of 5hmC protection and Tet1 oxidation. For the TAB kit we used, the 5hmC protection rate was nearly 100% and the conversion efficiency of recombinant Tet1 was 97.04%. For *M.SssI* assisted bisulfite sequencing (MAB-RRBS, to assay 5fC + 5caC levels), genomic DNA was methylated by *M.SssI* according to a previously published protocol [30], before bisulfite conversion. We assessed methylation efficiency by deep-sequencing MAB-treated genomic DNA containing unmethylated lambda phage DNA (1:1000 ratio) and by deep-sequencing MAB-treated lambda phage DNA alone. The estimated methylation efficiency is 96.20%; therefore, the expected error rate is 3.8%. We applied a binomial test to each called CpG site, accounting for the *M.SssI* methylation failure rate and sequencing coverage of that base. A *P* value for each CpG is calculated as 1-binom (number of times sequenced as T, total reads, fail rate). Because of the relatively low levels of 5fC and 5caC in the genome, we considered 5fC and 5caC only at sites with at least 20X coverage with a *P* value < 0.1. The coverage report can be found in Additional file 1: Table S20.

### DNA epigenetic modification analysis at CpG and non-CpG sites

The phyloepigenetic evolutionary tree was generated using the top 150,000 most variably methylated CpGs by incorporating 154 TCGA human Infinium 450 K datasets from GBM patients with our sample set. We applied a cutoff of 0.15/0.02/0.02 for average 5mC/5hmC/5fC + 5caC changes at promoter regions (TSS1500) or a particular feature (enhancers) to assess patterns of differential methylation. A cutoff of 0.2/0.05/0.05 was used for individual site comparisons. CpGs were annotated with Bedtools to genomic features. DNA modification of a promoter CpG is associated with TET expression when the Pearson correlation *P* value is < 0.01 and the modification exhibits a difference of at least 0.1 for 5mC, 5hmC, and 5fC between the maximum and minimum values. To characterize the genome-wide non-CpG methylation pattern in NSC and GSC, and the effect of differentiation, we used non-CpG sites captured by RRBS with at least 10× coverage and analyzed them in a strand-specific manner [63].

### Validation of DNA and histone epigenetic marks by chromatin and DNA immunoprecipitation coupled with real-time PCR (ChIP-qPCR and DIP-qPCR)

For ChIP-qPCR, samples were prepared as described above for ChIP-seq. For DIP-qPCR, DNA was extracted and incubated with 2 μL RNAse (GenDNA RNAse) at 37 °C for 10 min, followed by phenol-chloroform extraction. DNA samples were subject to shearing with a Covaris S220 to achieve 150-bp fragment size. Fragmented DNA was incubated with 2.5 μg 5mC (Epigentek), or 5 μg 5hmC (in-house) [64], 5fC (in-house), or 5caC (Cell Signaling) antibody, respectively, at room temperature for 3 h, followed by addition of 3 μL bridge antibody (Active Motif) and pre-washed magnetic protein beads at 4 °C overnight. The complex was washed with MeDIP (10 mM sodium phosphate, pH 7.0, 140 mM NaCl, 0.05% Triton X-100) and TE buffer, and then eluted at 65 °C for 15 min. Eluate was digested with proteinase K and purified using a Qiagen purification column. Eluted DNA was measured for enrichment at selected loci using primers in Additional file 1: Table S17. The specificity of each antibody was tested using spike-in controls; validation results are shown in Additional file 1: Table S21.

### Motif search and gene ontology analysis

Motifs were identified using MEME-suite/CentriMO/HOMER software at the default settings. Matching frequencies to the input sequences (± 300 bp of each highly modified 5hmC or 5fC/5caC site) and significance assessment (E-value) were considered for selecting the best hits. Ontology analysis was performed using the DAVID bioinformatics database with Benjamini correction for multiple testing and Ingenuity Pathway Analysis (IPA, Qiagen) with standard program parameters. GREAT (version 3.0.0) was used to predict genes near enhancer histone marks within 50-kb distance and to generate functional annotation and basic ontology analysis for these genes (e.g. biological process, gene cluster).

### Statistical analysis

Results of real-time PCR are expressed as the mean ± SEM. Significant differences were tested using one-way ANOVA on ranks. Post-hoc analysis was performed with pair-wise comparison using Dunn’s method. Differences in overall modification level among groups was performed with one-way ANOVA on ranks, with post-hoc analysis between each cell line performed with the Mann–Whitney U-test using Sigma Plot software 12.0 (Systat Software, Inc). Significance level was set at *P* < 0.05 level for all statistical tests.

## Additional files

Additional file 1: **Tables S1-S21**. (XLSX 1010 kb)

Additional file 2: **Figures S1-S10** and Supplementary figure legends related to main text figures. (PPTX 6670 kb)
