# Supplementary material for: Distinctive epigenomes characterize glioma stem cells and their response to differentiation cues
Source: Genome Biol. 2018 Mar 27;19:43. doi: 10.1186/s13059-018-1420-6 (PMC5872397; doi:10.1186/s13059-018-1420-6)
Supplement: Supplementary file 2 — Figures S1-S10 and Supplementary figure legends related to main text figures. (PPTX 6670 kb) [file 13059_2018_1420_MOESM2_ESM.pptx]

## Slide 1
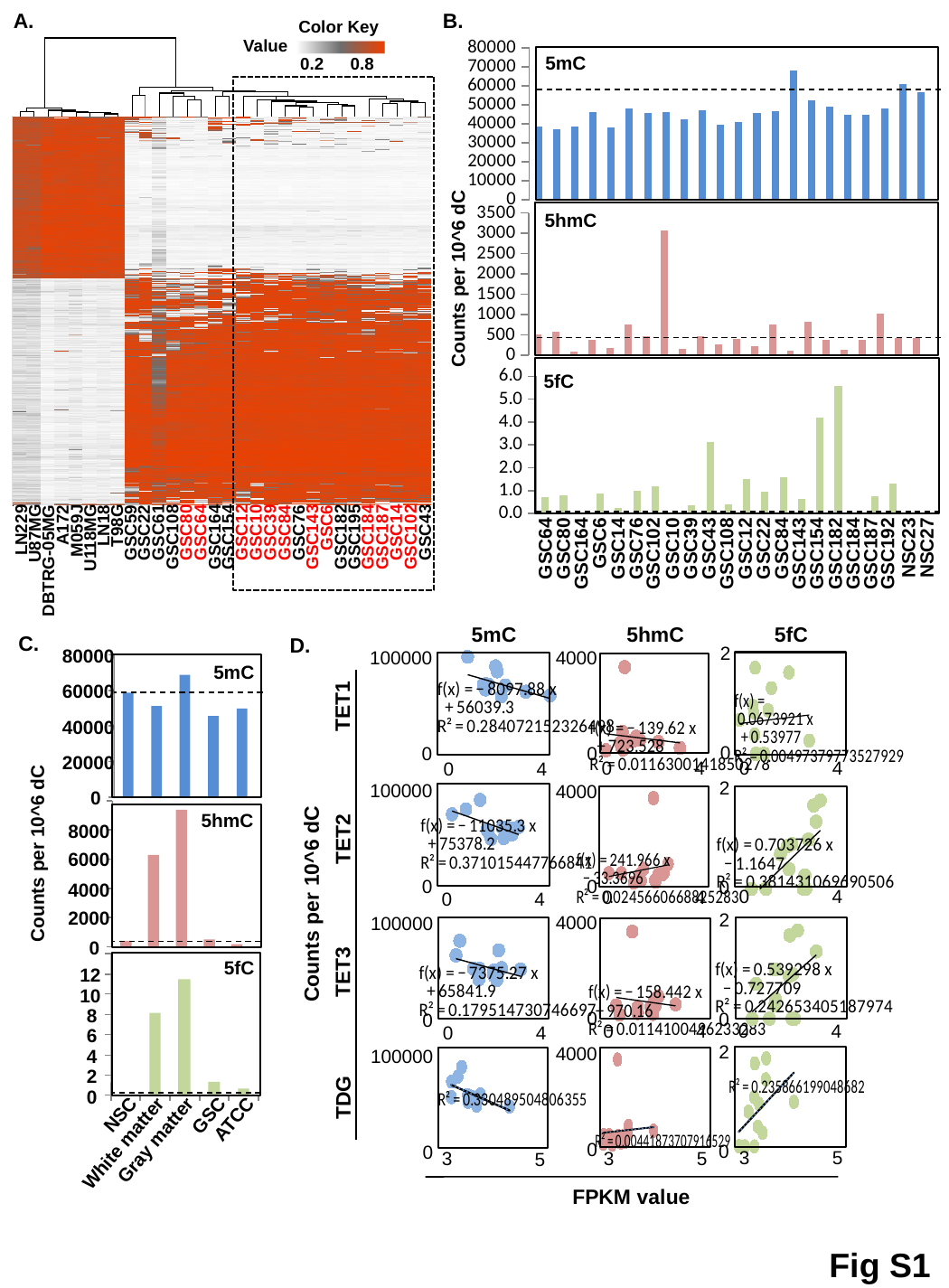

A.
B.
Color Key
Value
0.2
0.8
A172
M059J
GSC12
GSC22
GSC61
GSC80
GSC64
GSC10
GSC39
GSC84
GSC143
GSC6
GSC184
GSC187
GSC14
GSC102
U118MG
LN18
T98G
GSC59
GSC108
GSC164
GSC154
GSC76
GSC182
GSC195
GSC43
LN229
U87MG
DBTRG-05MG
### Chart
| Category | 5mC / 10^6 dC |
|---|---|
| GSC64 | 38322.23961189253 |
| GSC80 | 37218.57409142258 |
| GSC164 | 38520.23668926488 |
| GSC6 | 46249.57989621825 |
| GSC14 | 37839.27415208242 |
| GSC76 | 48009.2485194928 |
| GSC102 | 45660.78962598871 |
| GSC10 | 46225.782247685675 |
| GSC39 | 42532.97390733763 |
| GSC43 | 46919.12888979559 |
| GSC108 | 39569.87451193285 |
| GSC12 | 40764.0481414878 |
| GSC22 | 45538.58406979314 |
| GSC84 | 46689.81096438747 |
| GSC143 | 68218.14112610358 |
| GSC154 | 52086.30510669534 |
| GSC182 | 48772.56368334196 |
| GSC184 | 44555.62702888687 |
| GSC187 | 44590.06011477342 |
| GSC192 | 48197.68019648764 |
| sc23 | 60723.57819987863 |
| sc27 | 56765.069765760236 |5mC
### Chart
| Category | 5hmC / 10^6 dC |
|---|---|
| GSC64 | 507.456930820419 |
| GSC80 | 586.4314690451357 |
| GSC164 | 88.0005357843954 |
| GSC6 | 375.0998618479461 |
| GSC14 | 186.35333707624 |
| GSC76 | 763.034663750905 |
| GSC102 | 471.4451136086511 |
| GSC10 | 3067.298363788634 |
| GSC39 | 166.09051253291022 |
| GSC43 | 469.4651327571369 |
| GSC108 | 265.78546087357654 |
| GSC12 | 397.7030805025748 |
| GSC22 | 227.35827849005992 |
| GSC84 | 761.1711769687596 |
| GSC143 | 105.41215003020244 |
| GSC154 | 822.2294927274215 |
| GSC182 | 384.2519814091063 |
| GSC184 | 130.6460885229578 |
| GSC187 | 386.2582564154856 |
| GSC192 | 1030.627986719873 |
| sc23 | 431.2487331643752 |
| sc27 | 435.90092646782693 |5hmC
Counts per 10^6 dC
### Chart
| Category | 5fC / 10^6 dC |
|---|---|
| GSC64 | 0.7006113264796484 |
| GSC80 | 0.7986824576780769 |
| GSC164 | 0.0 |
| GSC6 | 0.8811127266901375 |
| GSC14 | 0.24323318147229037 |
| GSC76 | 0.9941993769572657 |
| GSC102 | 1.2033732442887664 |
| GSC10 | 0.0 |
| GSC39 | 0.3534161290482458 |
| GSC43 | 3.1453316333882353 |
| GSC108 | 0.40404839488098676 |
| GSC12 | 1.491007168096737 |
| GSC22 | 0.9657539735484704 |
| GSC84 | 1.579581201917876 |
| GSC143 | 0.622830379662801 |
| GSC154 | 4.184500966419928 |
| GSC182 | 5.561092114210293 |
| GSC184 | 0.0 |
| GSC187 | 0.7631952390375537 |
| GSC192 | 1.3115135541664735 |
| sc23 | 0.0 |
| sc27 | 0.0 |
5fC
GSC6
GSC64
GSC10
GSC184
NSC27
GSC192
GSC84
GSC182
GSC187
NSC23
GSC108
GSC14
GSC76
GSC39
GSC43
GSC12
GSC22
GSC80
GSC143
GSC164
GSC102
GSC154
5mC
5hmC
5fC
### Chart
| Category | |
|---|---|
### Chart
| Category | |
|---|---|
0
4
### Chart
| Category | |
|---|---|2
4000
100000
TET1
0
0
0
0
4
0
4
### Chart
| Category | |
|---|---|
### Chart
| Category | |
|---|---|2
100000
4000
### Chart
| Category | |
|---|---|
0
4
TET2
0
0
0
0
4
0
4
Counts per 10^6 dC
### Chart
| Category | |
|---|---|
### Chart
| Category | |
|---|---|2
4000
### Chart
| Category | |
|---|---|
0
4
100000
TET3
0
0
0
0
4
0
4
### Chart
| Category | |
|---|---|
### Chart
| Category | |
|---|---|2
4000
100000
### Chart
| Category | |
|---|---|
3
5
TDG
0
0
0
3
5
5
3
FPKM value
C.
80000
60000
40000
20000
0
5mC
8000
6000
4000
2000
0
5hmC
5fC
12
10
8
6
4
2
0
NSC
Gray matter
GSC
ATCC
White matter
Counts per 10^6 dC
D.
Fig S1

## Slide 2
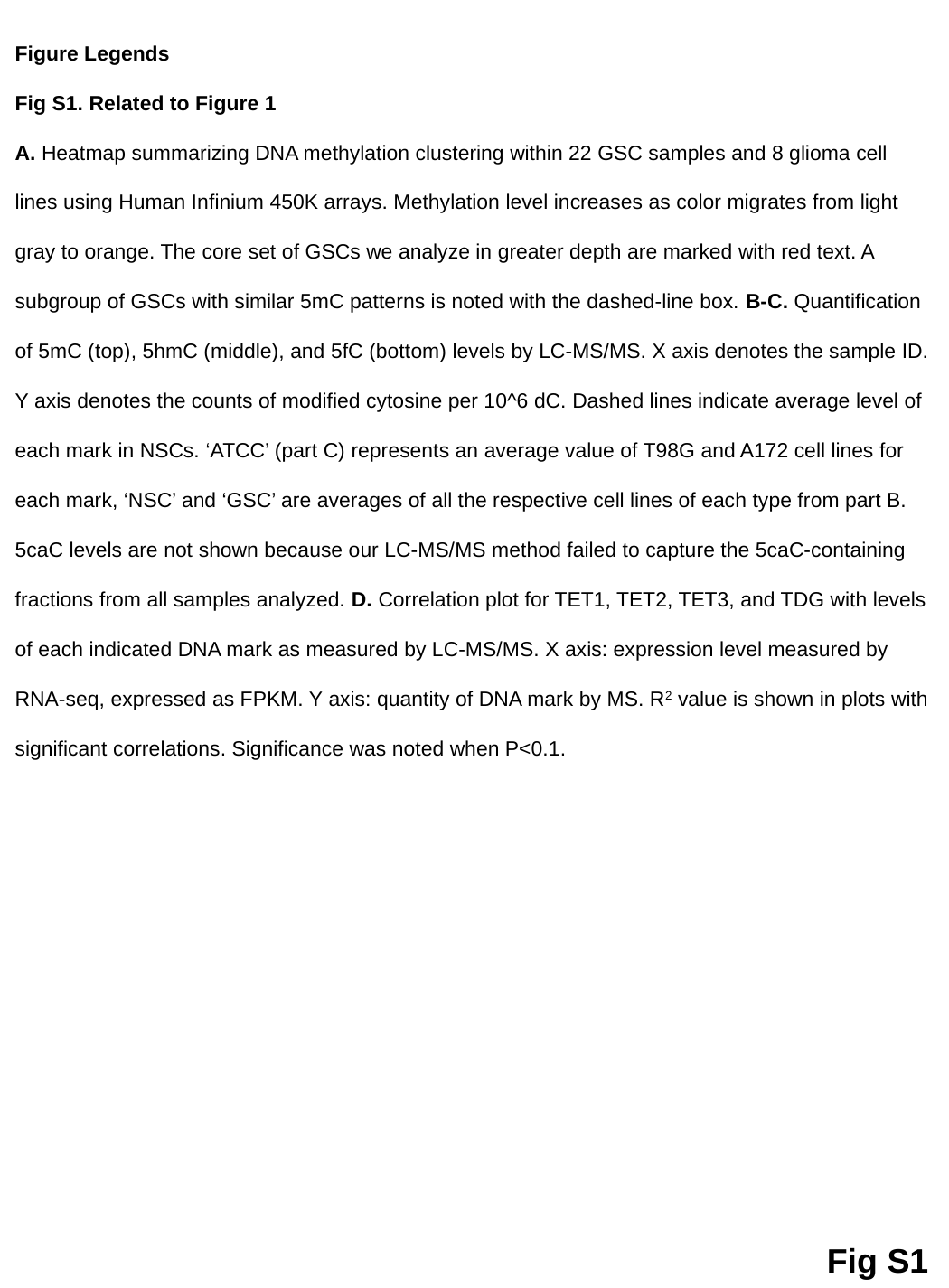

Figure Legends
Fig S1. Related to Figure 1
A. Heatmap summarizing DNA methylation clustering within 22 GSC samples and 8 glioma cell lines using Human Infinium 450K arrays. Methylation level increases as color migrates from light gray to orange. The core set of GSCs we analyze in greater depth are marked with red text. A subgroup of GSCs with similar 5mC patterns is noted with the dashed-line box. B-C. Quantification of 5mC (top), 5hmC (middle), and 5fC (bottom) levels by LC-MS/MS. X axis denotes the sample ID. Y axis denotes the counts of modified cytosine per 10^6 dC. Dashed lines indicate average level of each mark in NSCs. ‘ATCC’ (part C) represents an average value of T98G and A172 cell lines for each mark, ‘NSC’ and ‘GSC’ are averages of all the respective cell lines of each type from part B. 5caC levels are not shown because our LC-MS/MS method failed to capture the 5caC-containing fractions from all samples analyzed. D. Correlation plot for TET1, TET2, TET3, and TDG with levels of each indicated DNA mark as measured by LC-MS/MS. X axis: expression level measured by RNA-seq, expressed as FPKM. Y axis: quantity of DNA mark by MS. R2 value is shown in plots with significant correlations. Significance was noted when P<0.1.
Fig S1

## Slide 3
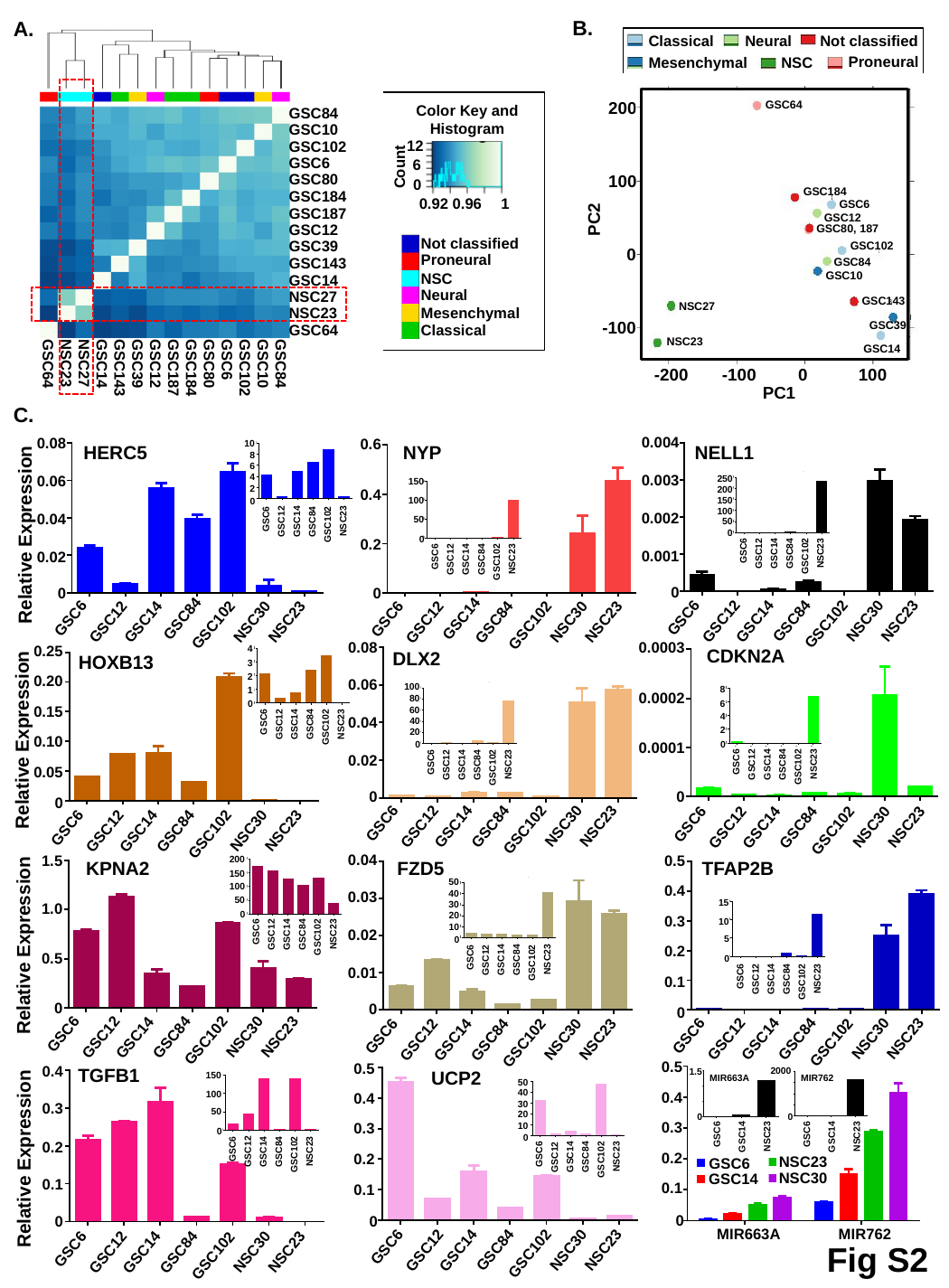

B.
A.
Neural
Classical
Not classified
Proneural
NSC
Mesenchymal
GSC84
GSC10
GSC102
GSC6
GSC80
GSC184
GSC187
GSC12
GSC39
GSC143
GSC14
NSC27
NSC23
GSC64
GSC84
GSC10
GSC102
GSC6
GSC80
GSC184
GSC187
GSC12
GSC39
GSC143
GSC14
NSC27
NSC23
GSC64
GSC64
200
100
GSC184
GSC6
PC2
GSC12
GSC80, 187
GSC102
0
GSC84
GSC10
GSC143
NSC27
GSC39
-100
NSC23
GSC14
-200
-100
0
100
PC1
Color Key and Histogram
12
6
Count
0
0.92
0.96
1
Not classified
Proneural
NSC
Neural
Mesenchymal
Classical
C.
0.004
0.08
0.6
10
8
6
4
2
0
NSC23
GSC12
GSC14
GSC6
GSC84
GSC102
HERC5
NYP
NELL1
0.003
0.06
250
200
150
100
50
0
NSC23
GSC6
GSC12
GSC14
GSC102
GSC84
150
100
50
0
NSC23
GSC12
GSC14
GSC84
GSC6
GSC102
0.4
0.002
0.04
Relative Expression
0.2
0.001
0.02
0
GSC6
GSC102
NSC30
NSC23
GSC12
GSC84
GSC14
0
GSC6
GSC84
GSC102
NSC30
NSC23
GSC12
GSC14
0
GSC14
GSC6
GSC102
NSC30
NSC23
GSC12
GSC84
0.08
0.0003
0.25
4
3
2
1
0
NSC23
GSC6
GSC12
GSC14
GSC102
GSC84
CDKN2A
DLX2
HOXB13
0.20
0.06
100
80
60
40
20
0
NSC23
GSC12
GSC84
GSC6
GSC14
GSC102
8
6
4
2
0
NSC23
GSC6
GSC12
GSC14
GSC84
GSC102
0.0002
0.15
0.04
Relative Expression
0.10
0.0001
0.02
0.05
0
0
GSC6
GSC102
NSC30
NSC23
GSC12
GSC84
GSC14
GSC6
GSC102
NSC30
NSC23
GSC12
GSC84
GSC14
GSC6
GSC102
NSC30
NSC23
GSC12
GSC84
GSC14
0
0.04
1.5
0.5
200
150
100
50
0
NSC23
GSC14
GSC84
GSC12
GSC6
GSC102
KPNA2
FZD5
TFAP2B
50
40
30
20
10
0
NSC23
GSC14
GSC6
GSC12
GSC84
GSC102
0.4
0.03
15
10
5
0
NSC23
GSC84
GSC6
GSC14
GSC102
GSC12
1.0
0.3
0.02
Relative Expression
0.2
0.5
0.01
0.1
0
GSC6
GSC102
NSC30
NSC23
GSC12
GSC84
GSC14
0
GSC6
GSC102
NSC30
NSC23
GSC12
GSC84
GSC14
GSC6
GSC102
NSC30
NSC23
GSC12
GSC84
GSC14
0
0.5
0.4
TGFB1
UCP2
150
100
50
0
GSC12
NSC23
GSC14
GSC84
GSC102
GSC6
50
40
30
20
10
0
GSC14
GSC6
NSC23
GSC102
GSC84
GSC12
0.4
0.3
0.3
0.2
Relative Expression
0.2
0.1
0.1
0
GSC6
GSC102
NSC30
NSC23
GSC12
GSC84
GSC14
0
GSC6
GSC102
NSC30
NSC23
GSC12
GSC84
GSC14
0.5
2000
1.5
MIR663A
MIR762
0.4
0
0
0.3
GSC14
GSC14
GSC6
GSC6
NSC23
NSC23
0.2
NSC23
GSC6
NSC30
GSC14
0.1
0
MIR663A
MIR762
Fig S2

## Slide 4
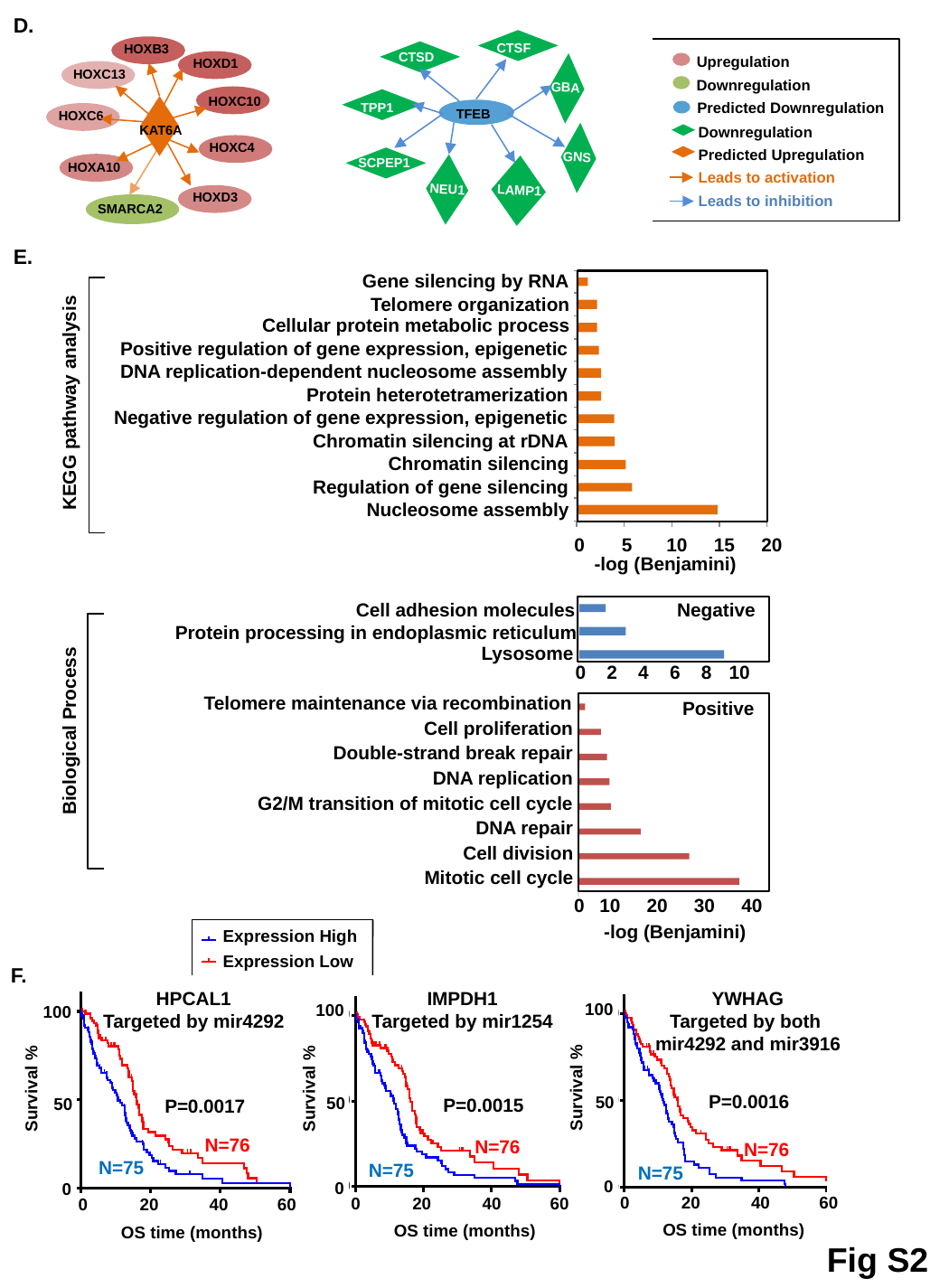

D.
CTSF
CTSD
GBA
TPP1
TFEB
GNS
SCPEP1
LAMP1
NEU1
HOXB3
HOXD1
HOXC13
HOXC6
KAT6A
HOXC4
HOXA10
HOXD3
SMARCA2
HOXC10
Upregulation
Downregulation
Downregulation
Predicted Upregulation
Predicted Downregulation
Leads to activation
Leads to inhibition
E.
Gene silencing by RNA
0
5
10
15
20
Telomere organization
Cellular protein metabolic process
Positive regulation of gene expression, epigenetic
DNA replication-dependent nucleosome assembly
Protein heterotetramerization
KEGG pathway analysis
Negative regulation of gene expression, epigenetic
Chromatin silencing at rDNA
Chromatin silencing
Regulation of gene silencing
Nucleosome assembly
-log (Benjamini)
Negative
0
2
4
6
8
10
Positive
0
10
20
30
40
-log (Benjamini)
Cell adhesion molecules
Protein processing in endoplasmic reticulum
Lysosome
Telomere maintenance via recombination
Cell proliferation
Double-strand break repair
DNA replication
G2/M transition of mitotic cell cycle
DNA repair
Cell division
Mitotic cell cycle
Biological Process
Expression High
Expression Low
F.
HPCAL1
Targeted by mir4292
IMPDH1
Targeted by mir1254
YWHAG
Targeted by both
mir4292 and mir3916
100
100
100
Survival %
Survival %
Survival %
50
50
50
P=0.0016
P=0.0015
P=0.0017
0
0
0
20
40
60
0
20
40
60
0
20
40
60
0
OS time (months)
OS time (months)
OS time (months)
N=76
N=76
N=76
N=75
N=75
N=75
Fig S2

## Slide 5
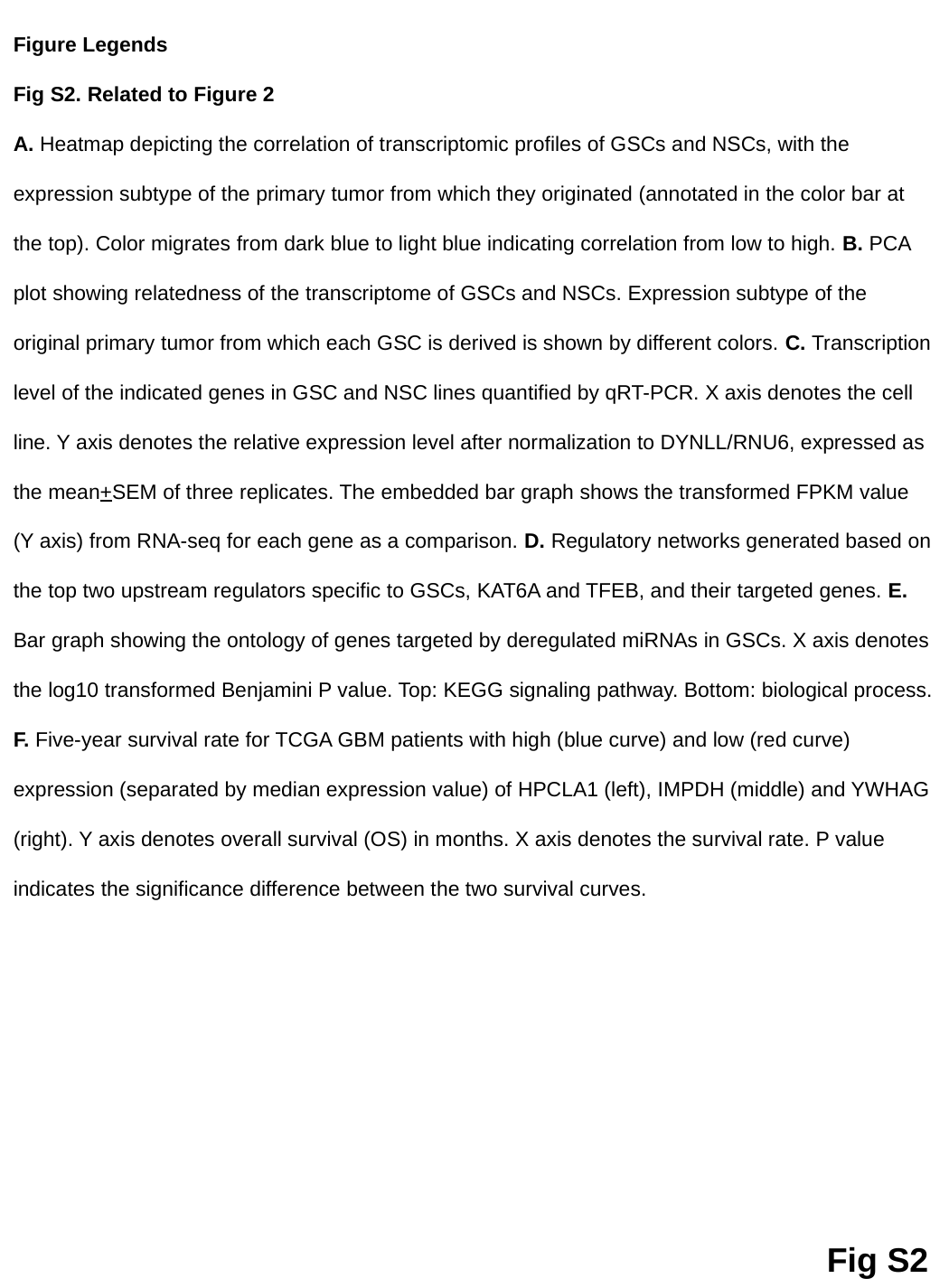

Figure Legends
Fig S2. Related to Figure 2
A. Heatmap depicting the correlation of transcriptomic profiles of GSCs and NSCs, with the expression subtype of the primary tumor from which they originated (annotated in the color bar at the top). Color migrates from dark blue to light blue indicating correlation from low to high. B. PCA plot showing relatedness of the transcriptome of GSCs and NSCs. Expression subtype of the original primary tumor from which each GSC is derived is shown by different colors. C. Transcription level of the indicated genes in GSC and NSC lines quantified by qRT-PCR. X axis denotes the cell line. Y axis denotes the relative expression level after normalization to DYNLL/RNU6, expressed as the mean+SEM of three replicates. The embedded bar graph shows the transformed FPKM value (Y axis) from RNA-seq for each gene as a comparison. D. Regulatory networks generated based on the top two upstream regulators specific to GSCs, KAT6A and TFEB, and their targeted genes. E. Bar graph showing the ontology of genes targeted by deregulated miRNAs in GSCs. X axis denotes the log10 transformed Benjamini P value. Top: KEGG signaling pathway. Bottom: biological process. F. Five-year survival rate for TCGA GBM patients with high (blue curve) and low (red curve) expression (separated by median expression value) of HPCLA1 (left), IMPDH (middle) and YWHAG (right). Y axis denotes overall survival (OS) in months. X axis denotes the survival rate. P value indicates the significance difference between the two survival curves.
Fig S2

## Slide 6
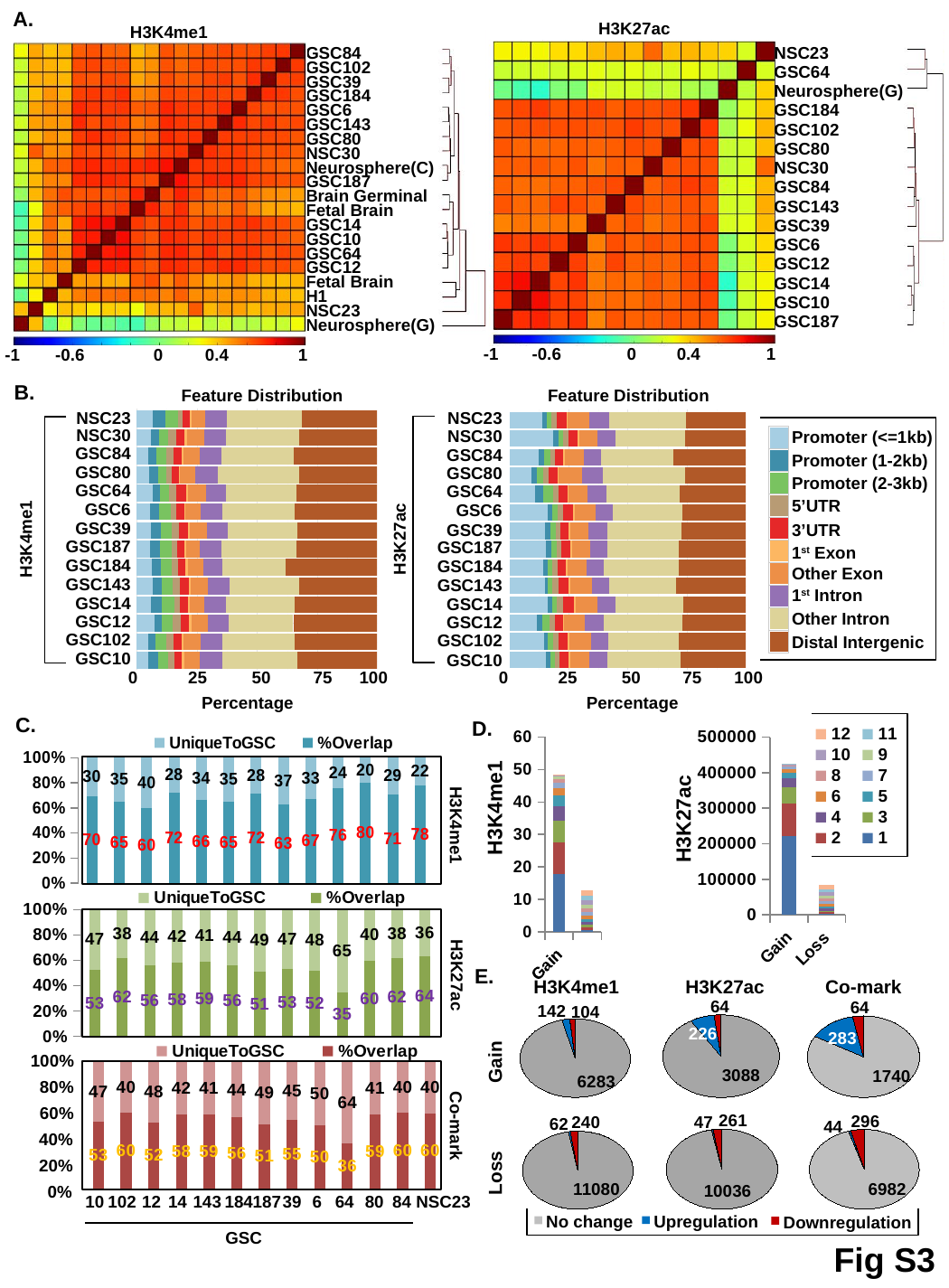

A.
H3K27ac
NSC23
GSC64
Neurosphere(G)
GSC184
GSC102
GSC80
NSC30
GSC84
GSC143
GSC39
GSC6
GSC12
GSC14
GSC10
GSC187
-1
-0.6
0
0.4
1
H3K4me1
GSC84
-1
-0.6
0
0.4
1
GSC102
GSC39
GSC184
GSC6
GSC143
GSC80
NSC30
Neurosphere(C)
GSC187
Brain Germinal
Fetal Brain
GSC14
GSC10
GSC64
GSC12
Fetal Brain
H1
NSC23
Neurosphere(G)
B.
Feature Distribution
Feature Distribution
NSC23
NSC23
Promoter (<=1kb)
Promoter (1-2kb)
Promoter (2-3kb)
5’UTR
3’UTR
1st Exon
Other Exon
1st Intron
Other Intron
Distal Intergenic
NSC30
NSC30
GSC84
GSC84
GSC80
GSC80
GSC64
GSC64
GSC6
GSC6
GSC39
GSC39
H3K4me1
H3K27ac
GSC187
GSC187
GSC184
GSC184
GSC143
GSC143
GSC14
GSC14
GSC12
GSC12
GSC102
GSC102
GSC10
GSC10
0
25
50
75
100
0
25
50
75
100
Percentage
Percentage
### Chart
| Category | %Overlap | UniqueToGSC |
|---|---|---|
| GBM10 | 69.53191489361701 | 30.468085106382986 |
| GBM102 | 65.01138050470064 | 34.98861949529936 |
| GBM12 | 59.93864423552697 | 40.06135576447303 |
| GBM14 | 72.24047501237011 | 27.759524987629888 |
| GBM143 | 66.45423057892133 | 33.545769421078674 |
| GBM184 | 65.24987629886195 | 34.75012370113805 |
| GBM187 | 71.64275111331024 | 28.35724888668976 |
| GBM39 | 63.086590796635335 | 36.913409203364665 |
| GBM6 | 67.41613062840179 | 32.58386937159821 |
| GBM64 | 76.04948045522019 | 23.95051954477981 |
| GBM80 | 80.16724393864423 | 19.832756061355767 |
| GBM84 | 70.8441365660564 | 29.155863433943594 |
| NSC23 | 78.24245423057891 | 21.757545769421085 |
### Chart
| Category | %Overlap | UniqueToGSC |
|---|---|---|
| GBM10 | 52.68013947924356 | 47.31986052075644 |
| GBM102 | 61.81501183537377 | 38.18498816462623 |
| GBM12 | 56.1213062180254 | 43.8786937819746 |
| GBM14 | 58.00223981267021 | 41.99776018732979 |
| GBM143 | 58.68309195958156 | 41.31690804041844 |
| GBM184 | 56.2345694723714 | 43.7654305276286 |
| GBM187 | 51.09699916007025 | 48.90300083992975 |
| GBM39 | 53.237547405126115 | 46.762452594873885 |
| GBM6 | 51.7384000610858 | 48.2615999389142 |
| GBM64 | 34.57456285474306 | 65.42543714525695 |
| GBM80 | 59.780091119651814 | 40.219908880348186 |
| GBM84 | 61.57321387665758 | 38.42678612334242 |
| NSC23 | 63.51268802972842 | 36.48731197027158 |
### Chart
| Category | %Overlap | UniqueToGSC |
|---|---|---|
| GBM10 | 52.7742942340384 | 47.2257057659616 |
| GBM102 | 60.19563581640332 | 39.80436418359668 |
| GBM12 | 52.339547975364376 | 47.660452024635624 |
| GBM14 | 58.33124320708971 | 41.66875679291029 |
| GBM143 | 58.97778892511775 | 41.02221107488225 |
| GBM184 | 56.196527603600586 | 43.803472396399414 |
| GBM187 | 51.12030766658307 | 48.87969233341693 |
| GBM39 | 54.549508123624 | 45.450491876376 |
| GBM6 | 50.28147033414152 | 49.71852966585848 |
| GBM64 | 35.95713847783073 | 64.04286152216926 |
| GBM80 | 58.636401638658974 | 41.363598361341026 |
| GBM84 | 60.0200652119388 | 39.9799347880612 |
| NSC23 | 59.510074408494276 | 40.489925591505724 |
H3K4me1
H3K27ac
Co-mark
10
102
12
14
143
184
187
39
6
64
80
84
NSC23
GSC
C.
D.
### Chart
| Category | 1 | 2 | 3 | 4 | 5 | 6 | 7 | 8 | 9 | 10 | 11 | 12 |
|---|---|---|---|---|---|---|---|---|---|---|---|---|
| Gain | 222354.0 | 91544.0 | 45004.0 | 25595.0 | 15718.0 | 10379.0 | 6275.0 | 3891.0 | 2009.0 | 947.0 | 345.0 | 39.0 |
| Loss | 2620.0 | 3875.0 | 4726.0 | 5692.0 | 6349.0 | 6997.0 | 7340.0 | 7914.0 | 8109.0 | 8797.0 | 9738.0 | 12700.0 |
### Chart
| Category | 1 | 2 | 3 | 4 | 5 | 6 | 7 | 8 | 9 | 10 | 11 | 12 |
|---|---|---|---|---|---|---|---|---|---|---|---|---|
| Gain | 177118.0 | 99056.0 | 65016.0 | 46028.0 | 32774.0 | 23419.0 | 16598.0 | 10905.0 | 6708.0 | 3847.0 | 1691.0 | 452.0 |
| Loss | 6142.0 | 7491.0 | 8137.0 | 8805.0 | 9626.0 | 10049.0 | 10482.0 | 11182.0 | 11847.0 | 12913.0 | 14159.0 | 17296.0 |H3K4me1
H3K27ac
E.
H3K4me1
H3K27ac
Co-mark
64
226
3088
64
283
1740
142
104
6283
Gain
261
47
10036
296
44
6982
240
62
11080
Loss
100%
80%
60%
40%
20%
0%
Upregulation
No change
Downregulation
Fig S3

## Slide 7
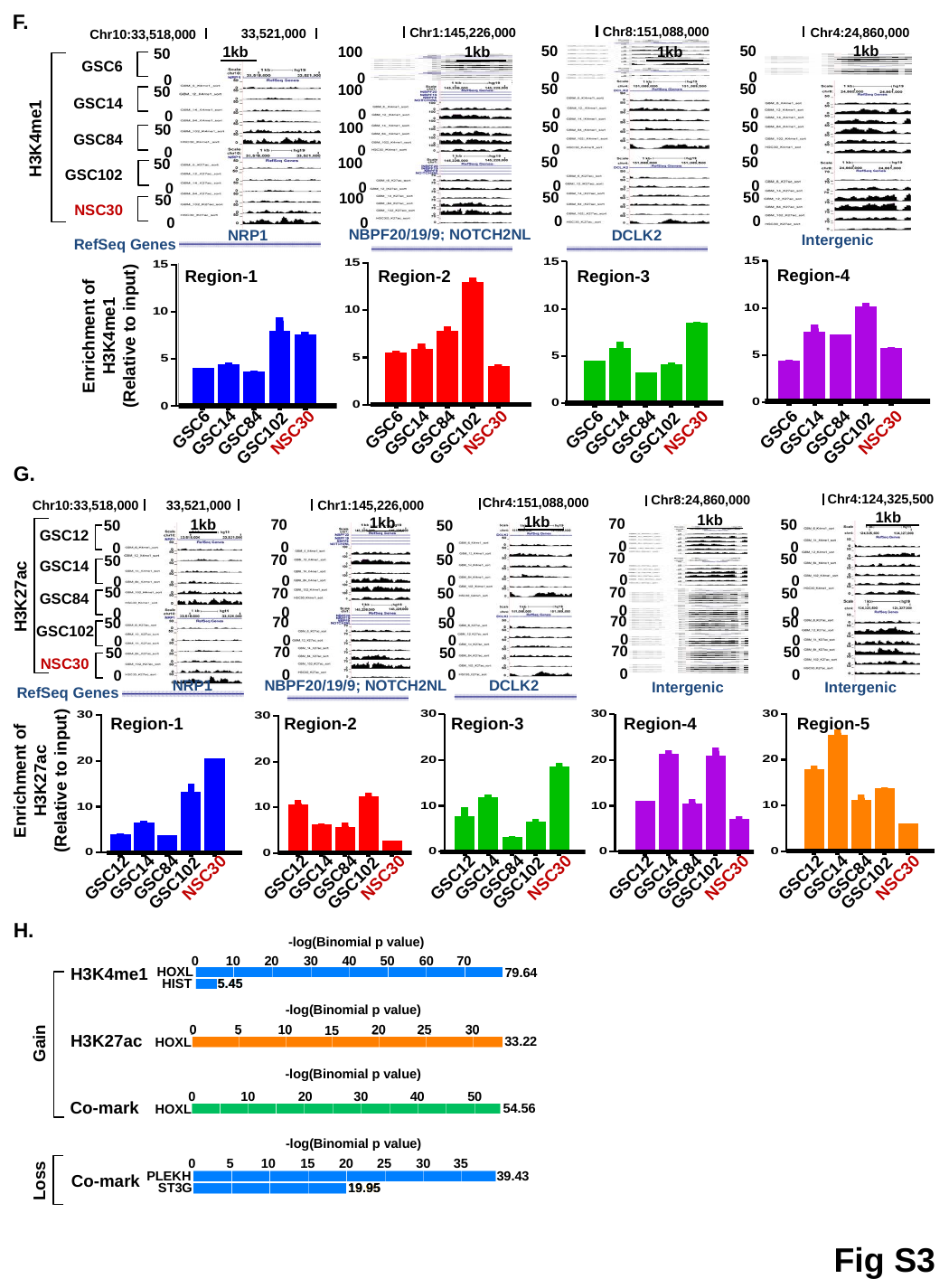

F.
Chr8:151,088,000
Chr1:145,226,000
Chr4:24,860,000
33,521,000
Chr10:33,518,000
1kb
50
50
100
1kb
1kb
1kb
50
GSC6
0
0
0
0
50
50
100
50
GSC14
0
0
0
0
50
50
100
50
H3K4me1
GSC84
0
0
0
0
50
50
100
50
GSC102
0
0
0
0
50
50
100
50
NSC30
0
0
0
0
NBPF20/19/9; NOTCH2NL
DCLK2
NRP1
Intergenic
RefSeq Genes
Enrichment of H3K4me1
(Relative to input)
GSC6
GSC14
GSC84
NSC30
GSC6
GSC14
GSC84
NSC30
GSC6
GSC14
GSC84
NSC30
GSC6
GSC14
GSC84
NSC30
GSC102
GSC102
GSC102
GSC102
Region-4
Region-1
Region-2
Region-3
G.
Chr4:124,325,500
Chr8:24,860,000
Chr4:151,088,000
Chr1:145,226,000
33,521,000
Chr10:33,518,000
1kb
1kb
1kb
1kb
70
1kb
50
70
50
50
GSC12
GSC14
H3K27ac
GSC84
GSC102
NSC30
0
0
0
0
0
70
50
70
50
50
0
0
0
0
0
70
50
50
70
50
0
0
0
0
0
70
50
70
50
50
0
0
0
0
0
70
50
70
50
50
0
0
0
0
0
DCLK2
NRP1
NBPF20/19/9; NOTCH2NL
Intergenic
Intergenic
RefSeq Genes
Region-1
Region-2
Region-3
Region-4
Region-5
Enrichment of H3K27ac
(Relative to input)
GSC12
GSC14
GSC84
NSC30
GSC12
GSC14
GSC84
NSC30
GSC12
GSC14
GSC84
NSC30
GSC12
GSC14
GSC84
NSC30
GSC12
GSC14
GSC84
NSC30
GSC102
GSC102
GSC102
GSC102
GSC102
H.
-log(Binomial p value)
0
10
20
30
40
50
60
70
H3K4me1
Gain
H3K27ac
Co-mark
Loss
Co-mark
HOXL
79.64
5.45
HIST
-log(Binomial p value)
10
0
5
20
25
30
15
33.22
HOXL
-log(Binomial p value)
0
10
20
30
40
50
54.56
HOXL
-log(Binomial p value)
0
5
10
15
20
25
30
35
PLEKH
39.43
19.95
ST3G
Fig S3

## Slide 8
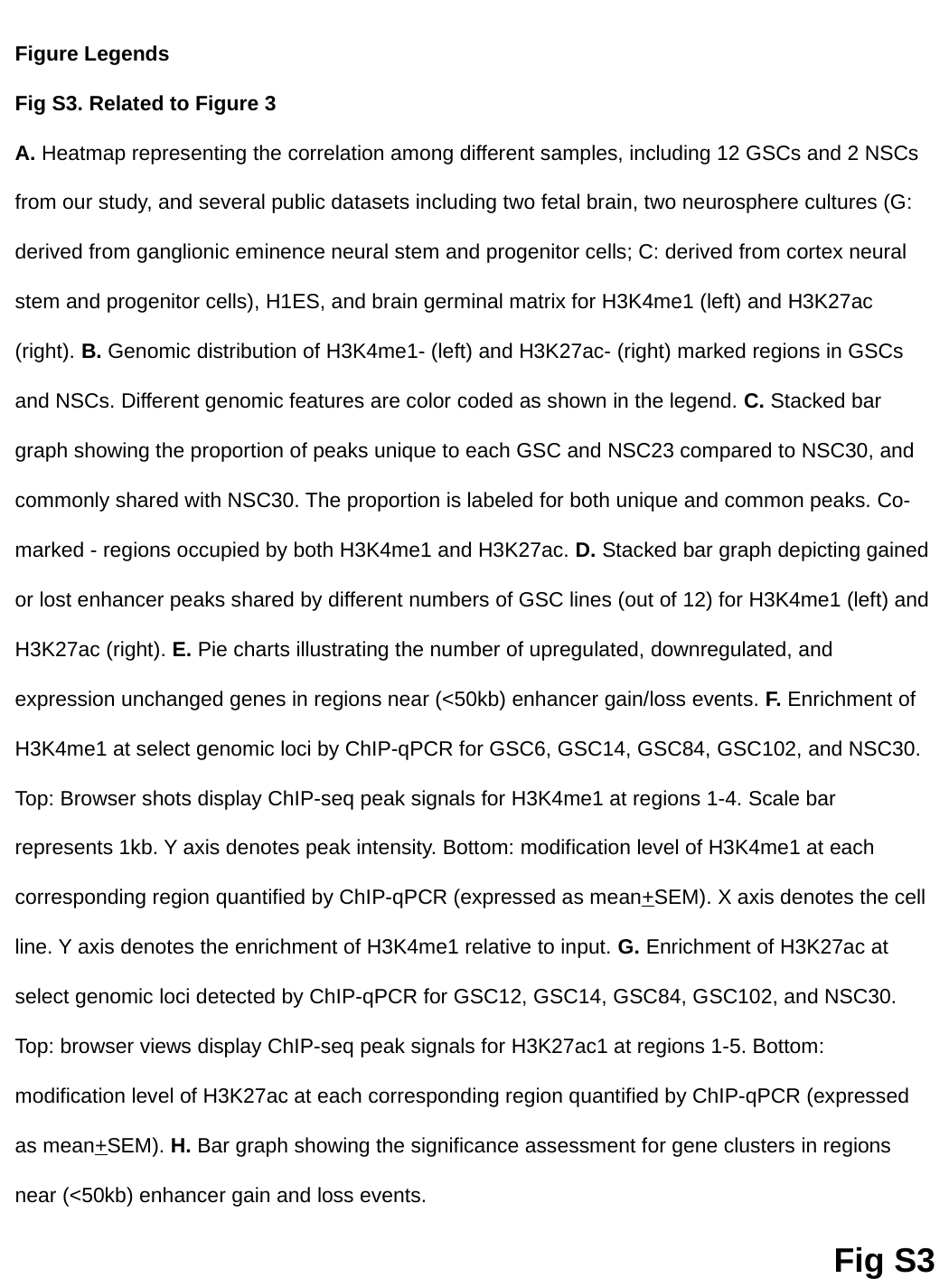

Figure Legends
Fig S3. Related to Figure 3
A. Heatmap representing the correlation among different samples, including 12 GSCs and 2 NSCs from our study, and several public datasets including two fetal brain, two neurosphere cultures (G: derived from ganglionic eminence neural stem and progenitor cells; C: derived from cortex neural stem and progenitor cells), H1ES, and brain germinal matrix for H3K4me1 (left) and H3K27ac (right). B. Genomic distribution of H3K4me1- (left) and H3K27ac- (right) marked regions in GSCs and NSCs. Different genomic features are color coded as shown in the legend. C. Stacked bar graph showing the proportion of peaks unique to each GSC and NSC23 compared to NSC30, and commonly shared with NSC30. The proportion is labeled for both unique and common peaks. Co-marked - regions occupied by both H3K4me1 and H3K27ac. D. Stacked bar graph depicting gained or lost enhancer peaks shared by different numbers of GSC lines (out of 12) for H3K4me1 (left) and H3K27ac (right). E. Pie charts illustrating the number of upregulated, downregulated, and expression unchanged genes in regions near (<50kb) enhancer gain/loss events. F. Enrichment of H3K4me1 at select genomic loci by ChIP-qPCR for GSC6, GSC14, GSC84, GSC102, and NSC30. Top: Browser shots display ChIP-seq peak signals for H3K4me1 at regions 1-4. Scale bar represents 1kb. Y axis denotes peak intensity. Bottom: modification level of H3K4me1 at each corresponding region quantified by ChIP-qPCR (expressed as mean+SEM). X axis denotes the cell line. Y axis denotes the enrichment of H3K4me1 relative to input. G. Enrichment of H3K27ac at select genomic loci detected by ChIP-qPCR for GSC12, GSC14, GSC84, GSC102, and NSC30. Top: browser views display ChIP-seq peak signals for H3K27ac1 at regions 1-5. Bottom: modification level of H3K27ac at each corresponding region quantified by ChIP-qPCR (expressed as mean+SEM). H. Bar graph showing the significance assessment for gene clusters in regions near (<50kb) enhancer gain and loss events.
Fig S3

## Slide 9
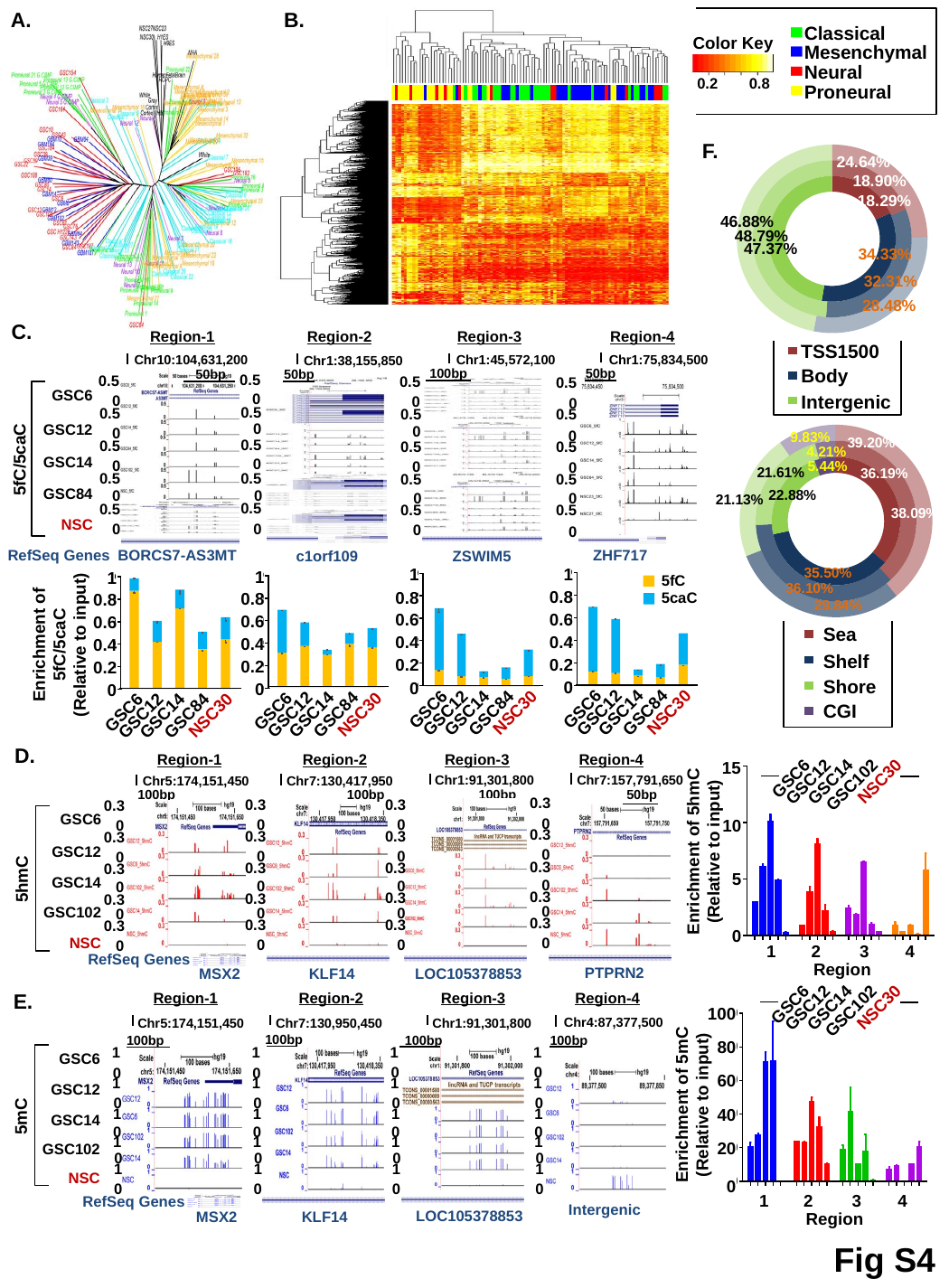

A.
B.
Classical
Mesenchymal
Neural
Proneural
Color Key
0.2
0.8
F.
C.
Region-1
Region-2
Region-3
Region-4
Chr10:104,631,200
Chr1:75,834,500
Chr1:45,572,100
Chr1:38,155,850
50bp
100bp
50bp
50bp
0.5
0.5
0.5
0.5
GSC6
GSC12
5fC/5caC
GSC14
GSC84
NSC
0
0
0
0
0.5
0.5
0.5
0.5
0
0
0
0
0.5
0.5
0.5
0.5
0
0
0
0
0.5
0.5
0.5
0.5
0
0
0
0
0.5
0.5
0.5
0.5
0
0
0
0
RefSeq Genes
BORCS7-AS3MT
ZHF717
c1orf109
ZSWIM5
1
1
1
0.8
0.6
0.4
0.2
0
1
0.8
0.6
0.4
0.2
0
5fC
5caC
0.8
0.8
0.6
0.6
Enrichment of 5fC/5caC
(Relative to input)
0.4
0.4
0.2
0.2
GSC6
GSC12
GSC14
NSC30
GSC84
GSC6
GSC12
GSC14
NSC30
GSC84
GSC6
GSC12
GSC14
NSC30
GSC84
GSC6
GSC12
GSC14
NSC30
GSC84
0
0
TSS1500
Body
Intergenic
Sea
Shelf
Shore
CGI
GSC6
GSC12
GSC14
NSC30
GSC102
15
10
Enrichment of 5hmC
(Relative to input)
5
0
1
2
3
4
Region
D.
Region-1
Region-2
Region-3
Region-4
Chr1:91,301,800
Chr7:157,791,650
Chr5:174,151,450
Chr7:130,417,950
100bp
100bp
100bp
50bp
0.3
0.3
0.3
0.3
GSC6
GSC12
5hmC
GSC14
GSC102
NSC
0
0
0
0
0.3
0.3
0.3
0.3
0
0
0
0
0.3
0.3
0.3
0.3
0
0
0
0
0.3
0.3
0.3
0.3
0
0
0
0
0.3
0.3
0.3
0.3
0
0
0
0
RefSeq Genes
PTPRN2
LOC105378853
MSX2
KLF14
GSC6
GSC12
GSC14
NSC30
GSC102
100
80
60
Enrichment of 5mC
(Relative to input)
40
20
0
1
2
3
4
Region
Region-1
Region-2
Region-3
Region-4
Chr7:130,950,450
Chr1:91,301,800
Chr5:174,151,450
100bp
100bp
100bp
100bp
E.
Chr4:87,377,500
1
1
1
1
GSC6
GSC12
5mC
GSC14
GSC102
NSC
0
0
0
0
1
1
1
1
0
0
0
0
1
1
1
1
0
0
0
0
1
1
1
1
0
0
0
0
1
1
1
1
0
0
0
0
RefSeq Genes
LOC105378853
MSX2
KLF14
Intergenic
Fig S4

## Slide 10
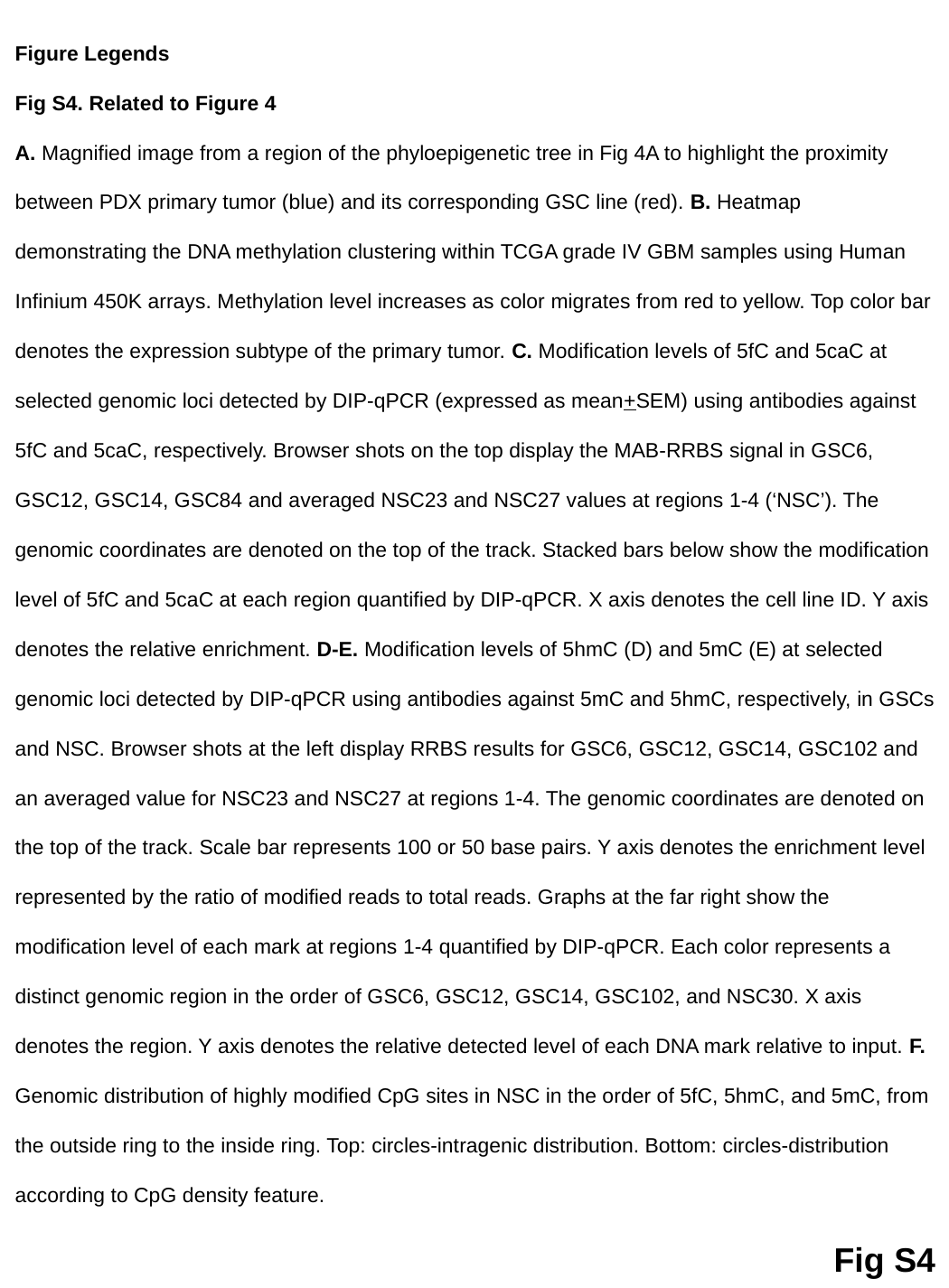

Figure Legends
Fig S4. Related to Figure 4
A. Magnified image from a region of the phyloepigenetic tree in Fig 4A to highlight the proximity between PDX primary tumor (blue) and its corresponding GSC line (red). B. Heatmap demonstrating the DNA methylation clustering within TCGA grade IV GBM samples using Human Infinium 450K arrays. Methylation level increases as color migrates from red to yellow. Top color bar denotes the expression subtype of the primary tumor. C. Modification levels of 5fC and 5caC at selected genomic loci detected by DIP-qPCR (expressed as mean+SEM) using antibodies against 5fC and 5caC, respectively. Browser shots on the top display the MAB-RRBS signal in GSC6, GSC12, GSC14, GSC84 and averaged NSC23 and NSC27 values at regions 1-4 (‘NSC’). The genomic coordinates are denoted on the top of the track. Stacked bars below show the modification level of 5fC and 5caC at each region quantified by DIP-qPCR. X axis denotes the cell line ID. Y axis denotes the relative enrichment. D-E. Modification levels of 5hmC (D) and 5mC (E) at selected genomic loci detected by DIP-qPCR using antibodies against 5mC and 5hmC, respectively, in GSCs and NSC. Browser shots at the left display RRBS results for GSC6, GSC12, GSC14, GSC102 and an averaged value for NSC23 and NSC27 at regions 1-4. The genomic coordinates are denoted on the top of the track. Scale bar represents 100 or 50 base pairs. Y axis denotes the enrichment level represented by the ratio of modified reads to total reads. Graphs at the far right show the modification level of each mark at regions 1-4 quantified by DIP-qPCR. Each color represents a distinct genomic region in the order of GSC6, GSC12, GSC14, GSC102, and NSC30. X axis denotes the region. Y axis denotes the relative detected level of each DNA mark relative to input. F. Genomic distribution of highly modified CpG sites in NSC in the order of 5fC, 5hmC, and 5mC, from the outside ring to the inside ring. Top: circles-intragenic distribution. Bottom: circles-distribution according to CpG density feature.
Fig S4

## Slide 11
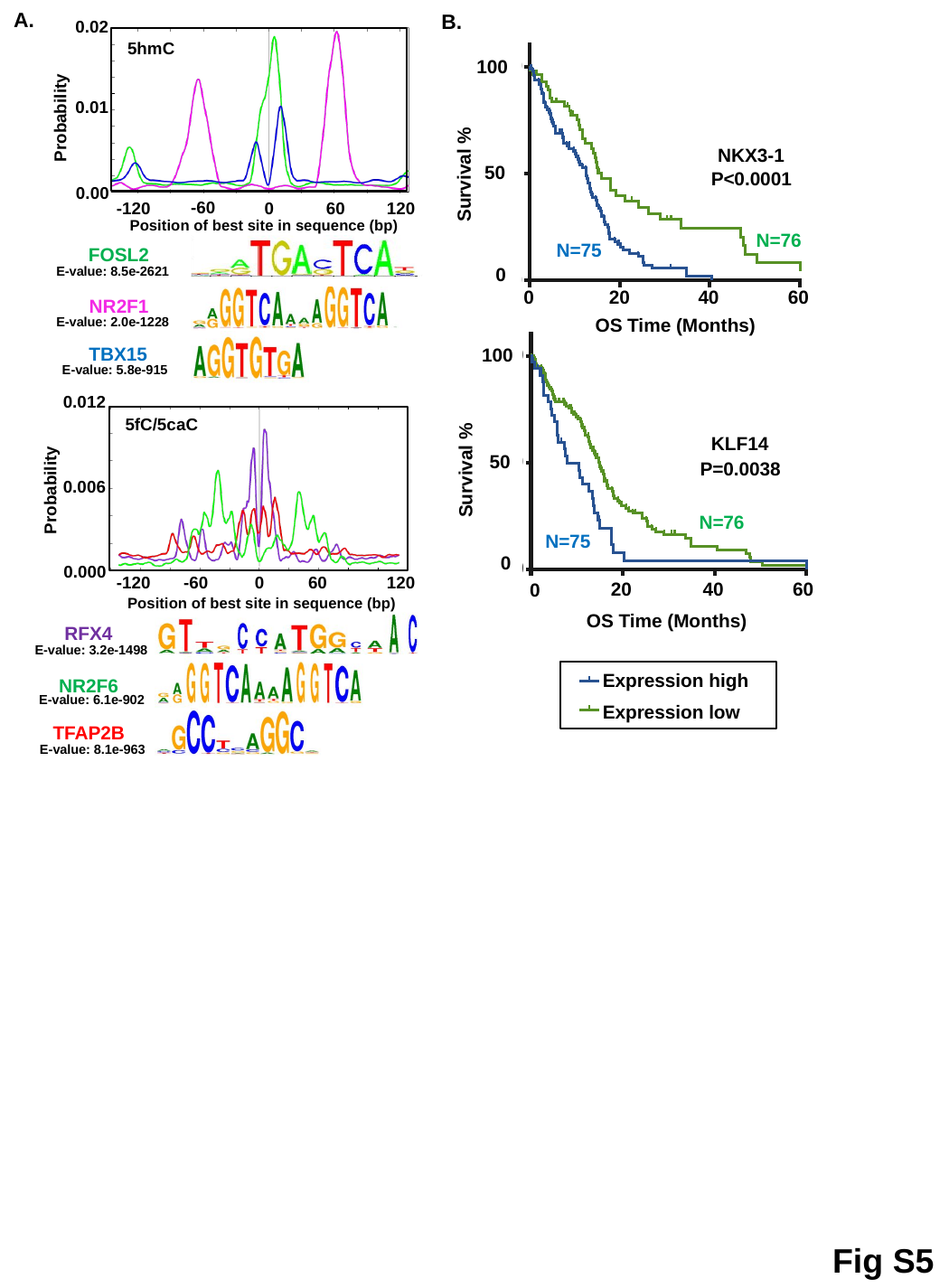

A.
B.
0.02
0.01
0.00
-60
120
-120
0
60
5hmC
Probability
Position of best site in sequence (bp)
FOSL2
E-value: 8.5e-2621
NR2F1
E-value: 2.0e-1228
TBX15
E-value: 5.8e-915
100
Survival %
NKX3-1
50
P<0.0001
0
N=76
N=75
20
40
60
0
OS Time (Months)
100
KLF14
50
0
OS Time (Months)
20
40
60
0
P=0.0038
0.012
5fC/5caC
0.006
Probability
0.000
-60
120
-120
0
60
Position of best site in sequence (bp)
RFX4
E-value: 3.2e-1498
NR2F6
E-value: 6.1e-902
TFAP2B
E-value: 8.1e-963
Survival %
N=76
N=75
Expression high
Expression low
Fig S5

## Slide 12
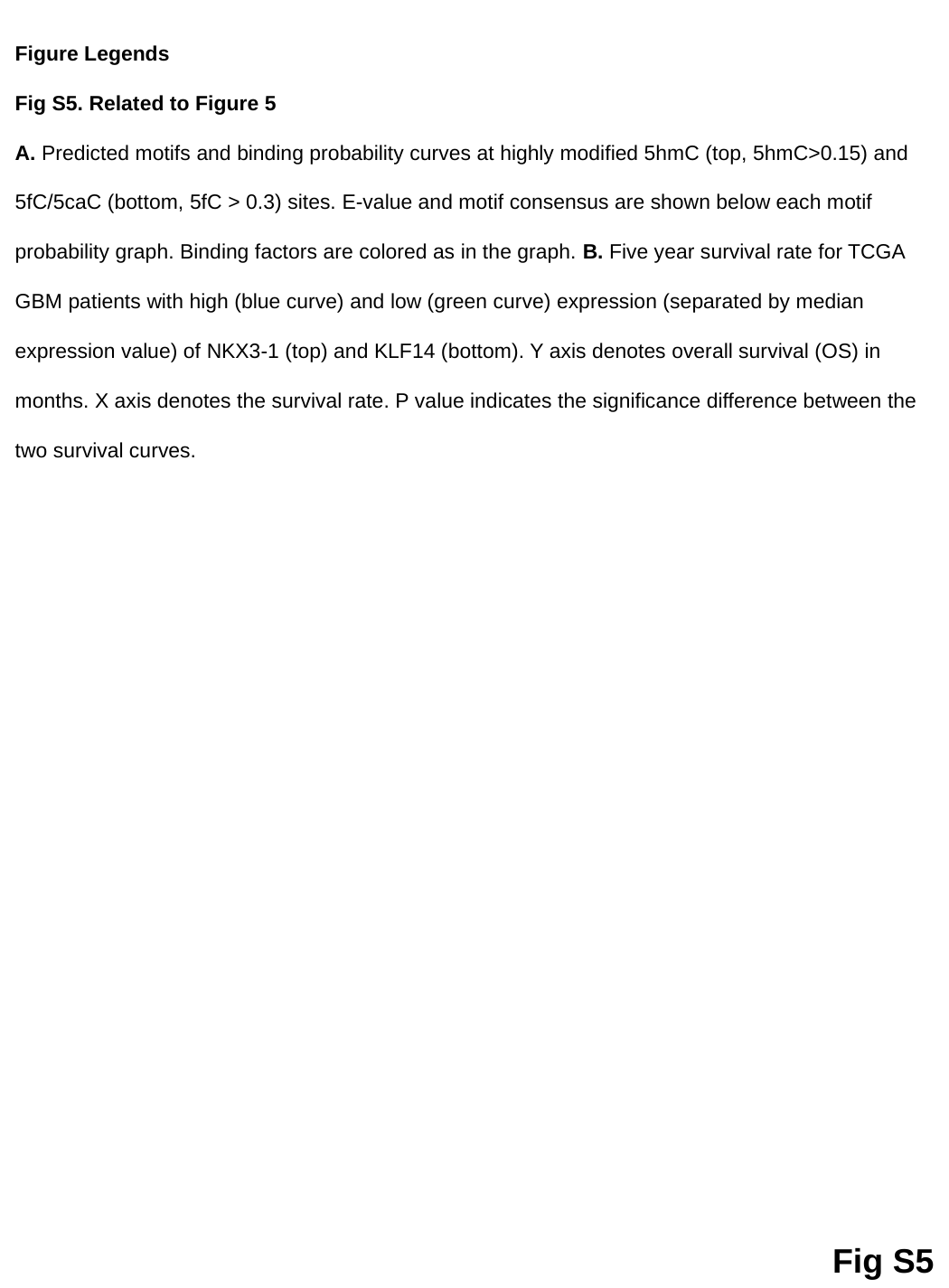

Figure Legends
Fig S5. Related to Figure 5
A. Predicted motifs and binding probability curves at highly modified 5hmC (top, 5hmC>0.15) and 5fC/5caC (bottom, 5fC > 0.3) sites. E-value and motif consensus are shown below each motif probability graph. Binding factors are colored as in the graph. B. Five year survival rate for TCGA GBM patients with high (blue curve) and low (green curve) expression (separated by median expression value) of NKX3-1 (top) and KLF14 (bottom). Y axis denotes overall survival (OS) in months. X axis denotes the survival rate. P value indicates the significance difference between the two survival curves.
Fig S5

## Slide 13
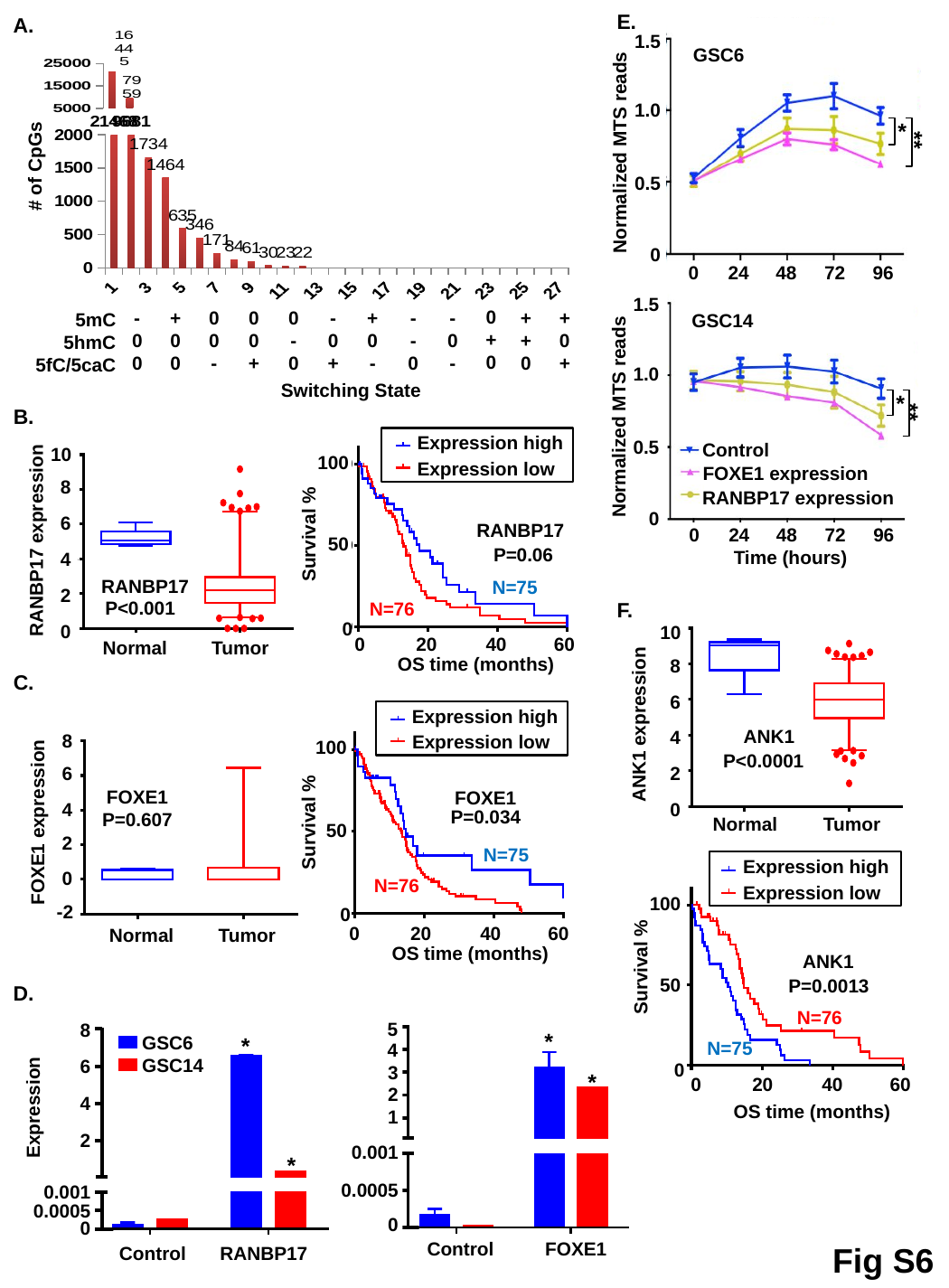

E.
A.
[unsupported chart]
[unsupported chart]
# of CpGs
0
+
0
-
0
0
+
0
0
0
0
-
0
0
+
0
-
0
-
0
+
+
0
-
-
-
0
-
0
-
+
+
0
+
0
+
5mC
5hmC
5fC/5caC
Switching State
*
**
1.5
GSC6
1.0
Normalized MTS reads
0.5
0
0
24
48
72
96
1.5
*
**
GSC14
1.0
0.5
Control
FOXE1 expression
RANBP17 expression
0
0
24
48
72
96
Normalized MTS reads
B.
Expression high
Expression low
10
8
6
RANBP17 expression
4
2
P<0.001
0
Normal
Tumor
100
Survival %
RANBP17
50
P=0.06
RANBP17
0
20
40
60
0
OS time (months)
Time (hours)
N=75
F.
N=76
10
8
6
ANK1 expression
4
P<0.0001
2
0
Normal
Tumor
C.
Expression high
Expression low
100
FOXE1
Survival %
P=0.034
50
0
20
40
60
0
OS time (months)
8
6
FOXE1
P=0.607
4
FOXE1 expression
2
0
-2
Normal
Tumor
ANK1
N=75
Expression high
Expression low
N=76
100
Survival %
ANK1
50
P=0.0013
D.
N=76
8
*
6
4
Expression
2
*
0.001
0.0005
0
Control
RANBP17
GSC6
GSC14
5
*
4
3
*
2
1
0.001
0.0005
0
Control
FOXE1
N=75
0
20
40
60
0
OS time (months)
Fig S6

## Slide 14
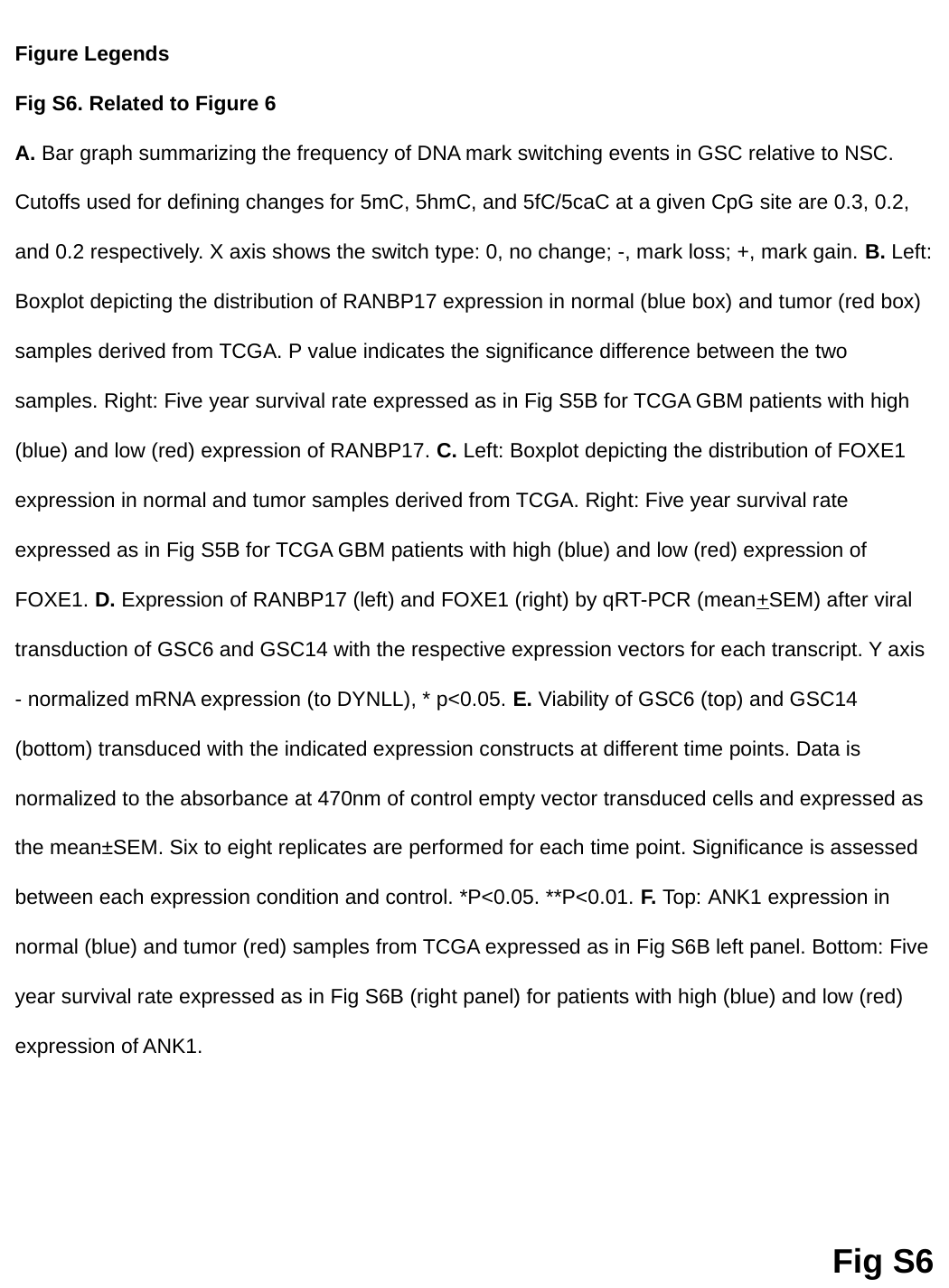

Figure Legends
Fig S6. Related to Figure 6
A. Bar graph summarizing the frequency of DNA mark switching events in GSC relative to NSC. Cutoffs used for defining changes for 5mC, 5hmC, and 5fC/5caC at a given CpG site are 0.3, 0.2, and 0.2 respectively. X axis shows the switch type: 0, no change; -, mark loss; +, mark gain. B. Left: Boxplot depicting the distribution of RANBP17 expression in normal (blue box) and tumor (red box) samples derived from TCGA. P value indicates the significance difference between the two samples. Right: Five year survival rate expressed as in Fig S5B for TCGA GBM patients with high (blue) and low (red) expression of RANBP17. C. Left: Boxplot depicting the distribution of FOXE1 expression in normal and tumor samples derived from TCGA. Right: Five year survival rate expressed as in Fig S5B for TCGA GBM patients with high (blue) and low (red) expression of FOXE1. D. Expression of RANBP17 (left) and FOXE1 (right) by qRT-PCR (mean+SEM) after viral transduction of GSC6 and GSC14 with the respective expression vectors for each transcript. Y axis - normalized mRNA expression (to DYNLL), * p<0.05. E. Viability of GSC6 (top) and GSC14 (bottom) transduced with the indicated expression constructs at different time points. Data is normalized to the absorbance at 470nm of control empty vector transduced cells and expressed as the mean±SEM. Six to eight replicates are performed for each time point. Significance is assessed between each expression condition and control. *P<0.05. **P<0.01. F. Top: ANK1 expression in normal (blue) and tumor (red) samples from TCGA expressed as in Fig S6B left panel. Bottom: Five year survival rate expressed as in Fig S6B (right panel) for patients with high (blue) and low (red) expression of ANK1.
Fig S6

## Slide 15
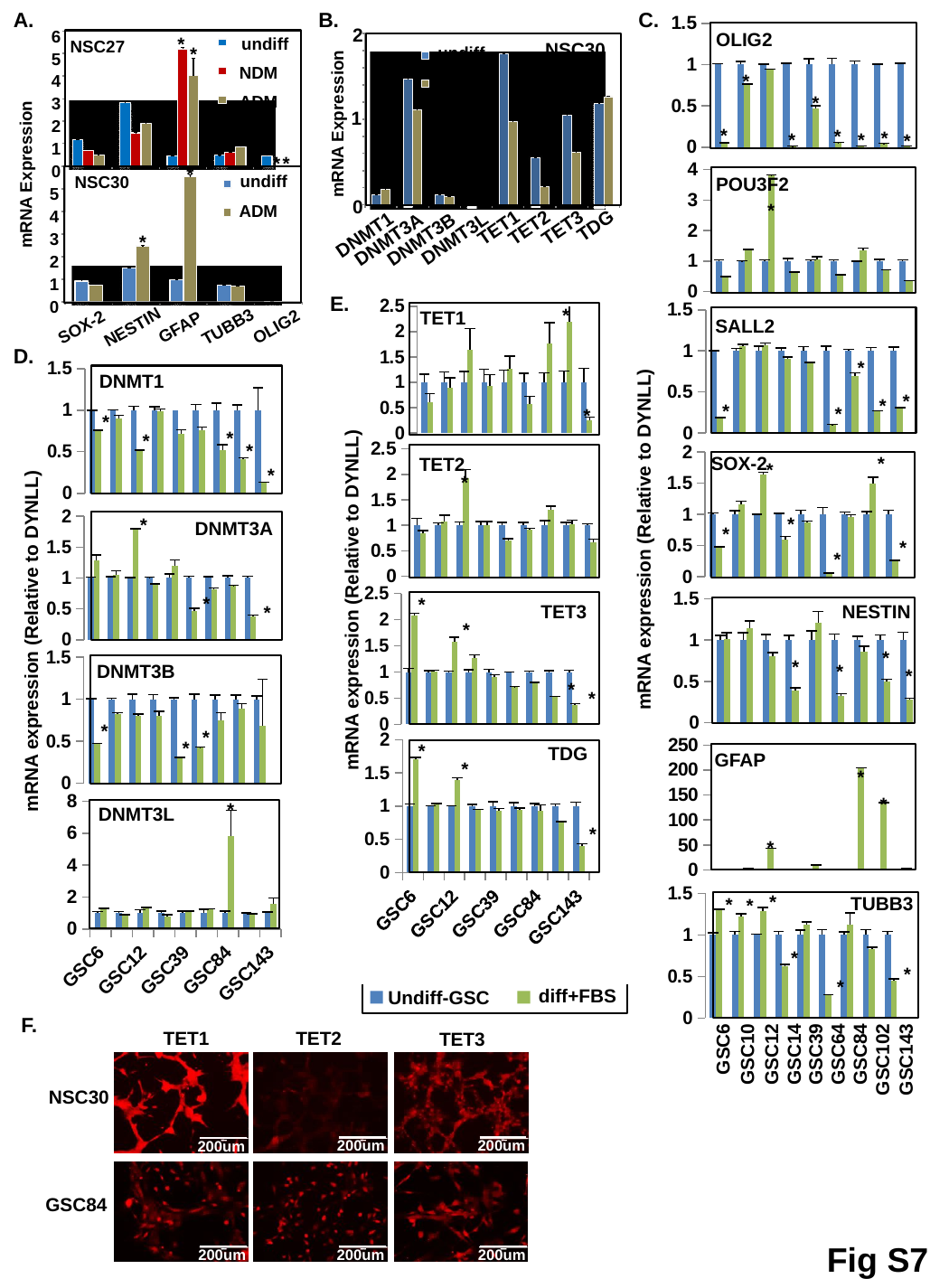

A.
C.
### Chart
| Category | | |
|---|---|---|
| GSC6 | 1.0 | 0.05140482499337273 |
| GSC10 | 0.9999999999999999 | 0.7653193275675475 |
| GSC12 | 1.0 | 0.93953967656719 |
| GSC14 | 1.0 | 0.01201656599328882 |
| GSC39 | 1.0 | 0.4695473202576802 |
| GSC64 | 1.0 | 0.05451813194011394 |
| GSC84 | 1.0 | 0.011963680728629793 |
| GSC102 | 1.0 | 0.04373705829984975 |
| GSC143 | 0.9999999999999999 | 0.01417896495695463 |OLIG2
*
*
*
*
*
*
*
*
### Chart
| Category | | |
|---|---|---|
| GSC6 | 1.0 | 0.4761560324260774 |
| GSC10 | 1.0 | 1.3661633177640504 |
| GSC12 | 1.0 | 3.794177970414587 |
| GSC14 | 1.0 | 0.6372908042339027 |
| GSC39 | 1.0 | 1.0665430339247053 |
| GSC64 | 1.0 | 0.5337602454729664 |
| GSC84 | 1.0 | 1.3535024928551063 |
| GSC102 | 1.0 | 0.7183620614754456 |
| GSC143 | 1.0 | 0.3498565572926581 |POU3F2
*
### Chart
| Category | | |
|---|---|---|
| GSC6 | 1.0 | 0.18495009329672715 |
| GSC10 | 1.0000000000000002 | 1.0603311495638295 |
| GSC12 | 0.9999999999999999 | 1.063252879861491 |
| GSC14 | 1.0 | 0.8980299321231908 |
| GSC39 | 1.0000000000000002 | 0.8457293776081991 |
| GSC64 | 1.0 | 0.09898568508684986 |
| GSC84 | 1.0 | 0.6956269110187241 |
| GSC102 | 1.0 | 0.26592484796330007 |
| GSC143 | 1.0 | 0.30747582426593467 |SALL2
*
*
*
*
*
### Chart
| Category | | |
|---|---|---|
| GSC6 | 1.0 | 0.46189076940875173 |
| GSC10 | 1.0 | 1.1556130100287962 |
| GSC12 | 1.0 | 1.630771271278237 |
| GSC14 | 0.9999999999999999 | 0.5926251888579643 |
| GSC39 | 1.0 | 0.8689222166709344 |
| GSC64 | 1.0 | 0.05377775414438272 |
| GSC84 | 1.0 | 0.9527695254766066 |
| GSC102 | 1.0 | 1.4967966797095733 |
| GSC143 | 1.0 | 0.25303402444254275 |SOX-2
*
*
*
*
mRNA expression (Relative to DYNLL)
*
*
### Chart
| Category | | |
|---|---|---|
| GSC6 | 0.9999999999999999 | 1.0119065363576667 |
| GSC10 | 0.9999999999999999 | 1.14340800760914 |
| GSC12 | 0.9999999999999999 | 0.8057954893370165 |
| GSC14 | 1.0 | 0.38640656622838965 |
| GSC39 | 1.0 | 1.2141948843950447 |
| GSC64 | 1.0 | 0.3222425626958688 |
| GSC84 | 1.0 | 0.8547184306900175 |
| GSC102 | 1.0 | 0.49806692272030323 |
| GSC143 | 1.0 | 0.2760107536931651 |NESTIN
*
*
*
*
### Chart
| Category | | |
|---|---|---|
| GSC6 | 1.0 | 0.5640079297504506 |
| GSC10 | 1.0 | 2.181218053008097 |
| GSC12 | 1.0 | 43.557866159336385 |
| GSC14 | 1.0 | 1.1527486471774964 |
| GSC39 | 1.0 | 9.917231370548427 |
| GSC64 | 0.9999999999999999 | 0.024055858718274245 |
| GSC84 | 0.9999999999999999 | 204.0525264231719 |
| GSC102 | 1.0 | 135.00874870938898 |
| GSC143 | 1.0 | 3.1862710652978032 |GFAP
*
*
*
### Chart
| Category | | |
|---|---|---|
| GSC6 | 1.0 | 1.2920967619719161 |
| GSC10 | 1.0 | 1.2260897499052028 |
| GSC12 | 1.0 | 1.2841273480713387 |
| GSC14 | 1.0 | 0.6240004415882037 |
| GSC39 | 1.0 | 1.1274409702489763 |
| GSC64 | 0.9999999999999999 | 0.2731141806602699 |
| GSC84 | 1.0 | 1.1217731711016978 |
| GSC102 | 1.0 | 0.82784287689829 |
| GSC143 | 1.0000000000000002 | 0.44781340800565766 |TUBB3
*
*
*
*
*
*
GSC6
GSC10
GSC12
GSC14
GSC39
GSC64
GSC84
GSC102
GSC143
B.
2
NSC30
*
*
*
*
TDG
TET1
TET2
TET3
DNMT3L
DNMT3A
DNMT3B
DNMT1
1
0
mRNA Expression
6
*
undiff
NDM
ADM
*
NSC27
5
4
3
*
*
2
*
*
*
1
*
*
*
mRNA Expression
0
undiff
ADM
NSC30
5
4
*
3
2
1
0
OLIG2
TUBB3
SOX-2
NESTIN
GFAP
undiff
ADM
E.
### Chart
| Category | | |
|---|---|---|
| GSC6 | 0.9999999999999999 | 0.6106576993902748 |
| GSC10 | 1.0 | 0.8932378419752619 |
| GSC12 | 1.0 | 1.6434560260668183 |
| GSC14 | 1.0 | 0.9209292788542072 |
| GSC39 | 1.0 | 1.2595491960334642 |
| GSC64 | 1.0 | 0.574034467555298 |
| GSC84 | 1.0 | 1.7648913315193993 |
| GSC102 | 1.0 | 2.1996108402678467 |
| GSC143 | 1.0 | 0.24813130022503904 |
### Chart
| Category | | |
|---|---|---|
| GSC6 | 1.0 | 0.8352757365747727 |
| GSC10 | 0.9999999999999998 | 1.0802474775576596 |
| GSC12 | 1.0 | 1.9282376645752213 |
| GSC14 | 1.0 | 1.0000000000000013 |
| GSC39 | 1.0 | 0.7066316971209885 |
| GSC64 | 1.0 | 0.9099323503398391 |
| GSC84 | 1.0 | 1.3146525599884824 |
| GSC102 | 0.9999999999999999 | 1.0259586231882385 |
| GSC143 | 1.0 | 0.6586613328657593 |
### Chart
| Category | | |
|---|---|---|
| GSC6 | 1.0 | 2.08086926118115 |
| GSC10 | 1.0 | 1.0138681620805676 |
| GSC12 | 0.9999999999999999 | 1.5768859399257042 |
| GSC14 | 1.0 | 1.266182025604101 |
| GSC39 | 1.0 | 0.8979653389581861 |
| GSC64 | 1.0 | 0.7169776240079135 |
| GSC84 | 1.0 | 0.7793751317813633 |
| GSC102 | 1.0 | 0.5266046211175415 |
| GSC143 | 1.0 | 0.3750615236198934 |
### Chart
| Category | | |
|---|---|---|
| GSC6 | 1.0 | 1.6987986883711201 |
| GSC10 | 0.9999999999999998 | 1.0246060483712425 |
| GSC12 | 1.0 | 1.3856335645752766 |
| GSC14 | 0.9999999999999999 | 0.9490628194458827 |
| GSC39 | 1.0 | 0.9349079756886844 |
| GSC64 | 1.0 | 0.9387110835896524 |
| GSC84 | 0.9999999999999999 | 0.927176526701581 |
| GSC102 | 1.0000000000000002 | 0.7601241449016357 |
| GSC143 | 1.0 | 0.39726993345010125 |
TET1
TET2
mRNA expression (Relative to DYNLL)
TET3
TDG
*
*
*
*
*
*
*
*
*
*
D.
### Chart
| Category | | |
|---|---|---|
| GSC6 | 1.0 | 0.7552544356317054 |
| GSC10 | 1.0 | 0.8990386934973538 |
| GSC12 | 1.0 | 0.5048619072755384 |
| GSC14 | 1.0 | 0.9824489794149949 |
| GSC39 | 1.0 | 0.7183731964565621 |
| GSC64 | 1.0 | 0.7594362734227098 |
| GSC84 | 0.9999999999999999 | 0.5196466924104556 |
| GSC102 | 0.9999999999999999 | 0.4111954684435324 |
| GSC143 | 1.0 | 0.1341224733295111 |DNMT1
### Chart
| Category | | |
|---|---|---|
| GSC6 | 1.0000000000000002 | 1.2778149999806039 |
| GSC10 | 1.0 | 1.047894541382231 |
| GSC12 | 1.0 | 1.7962215955911476 |
| GSC14 | 1.0 | 0.8981054045903352 |
| GSC39 | 1.0 | 1.202522522234243 |
| GSC64 | 1.0 | 0.4682317721465853 |
| GSC84 | 1.0 | 0.8179659067563618 |
| GSC102 | 1.0 | 0.8759901336410467 |
| GSC143 | 1.0 | 0.38164727986122593 |DNMT3A
### Chart
| Category | | |
|---|---|---|
| GSC6 | 1.0 | 0.4697754805934601 |
| GSC10 | 1.0 | 0.829284683997862 |
| GSC12 | 1.0 | 0.8054178475164577 |
| GSC14 | 1.0 | 0.8069708648315153 |
| GSC39 | 1.0 | 0.29443696442407985 |
| GSC64 | 1.0 | 0.42126774026354025 |
| GSC84 | 1.0 | 0.7474927910515857 |
| GSC102 | 1.0 | 0.8926137172313904 |
| GSC143 | 1.0 | 0.6886063387498101 |
DNMT3B
### Chart
| Category | | |
|---|---|---|
| GSC6 | 1.0 | 1.249302122301334 |
| GSC10 | 1.0 | 0.8797058201140471 |
| GSC12 | 1.0 | 1.267038603425846 |
| GSC14 | 1.0 | 0.7596459553157406 |
| GSC39 | 1.0 | 1.0754694599666923 |
| GSC64 | 1.0 | 1.2693908486564114 |
| GSC84 | 1.0 | 5.8406240639278835 |
| GSC102 | 1.0 | 0.8998805568618766 |
| GSC143 | 1.0 | 1.5543720203865392 |DNMT3L
mRNA expression (Relative to DYNLL)
*
*
*
*
*
*
*
*
*
*
*
*
diff+FBS
Undiff-GSC
F.
TET1
TET2
TET3
NSC30
GSC84
200um
200um
200um
200um
200um
200um
Fig S7

## Slide 16
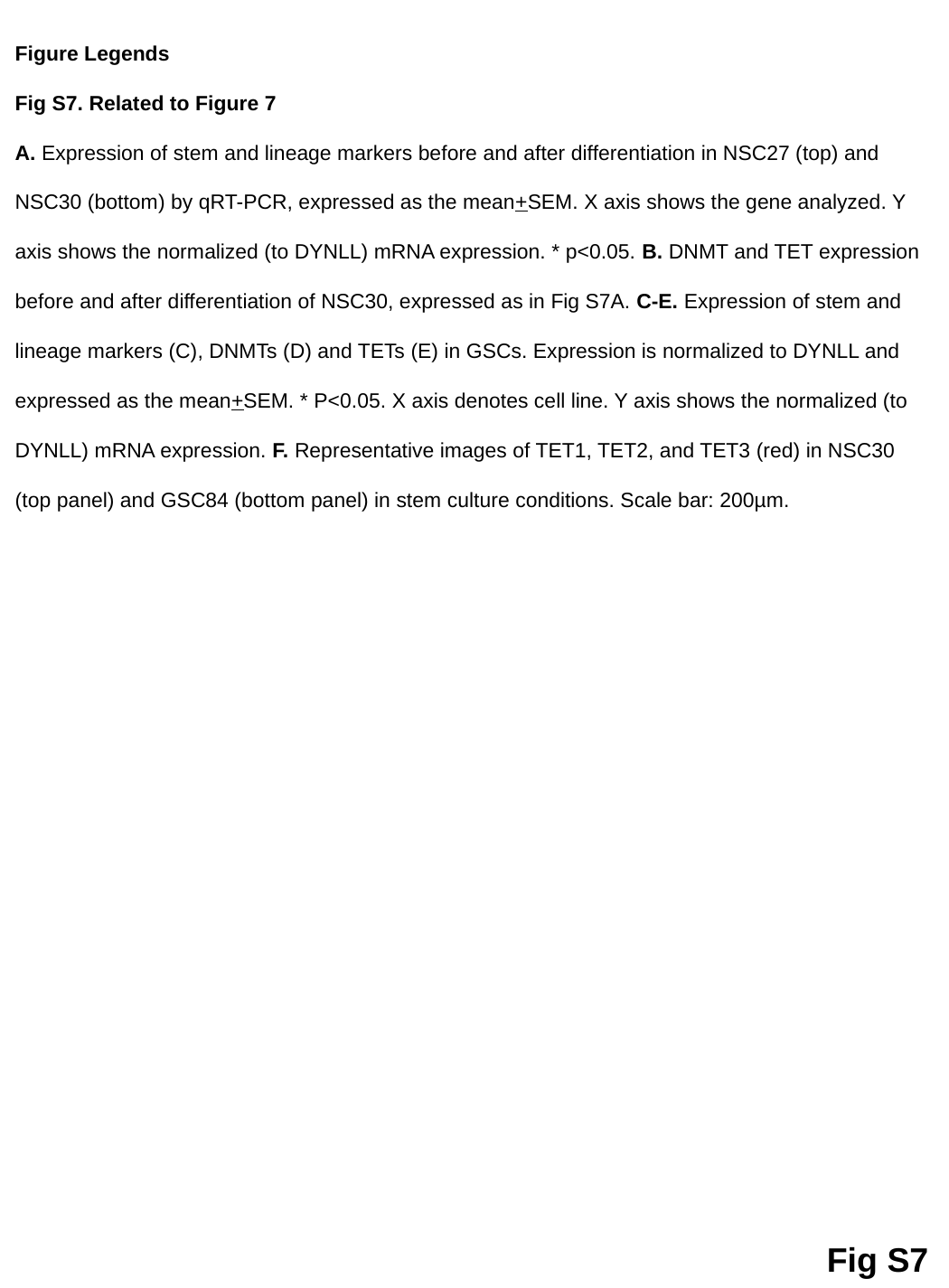

Figure Legends
Fig S7. Related to Figure 7
A. Expression of stem and lineage markers before and after differentiation in NSC27 (top) and NSC30 (bottom) by qRT-PCR, expressed as the mean+SEM. X axis shows the gene analyzed. Y axis shows the normalized (to DYNLL) mRNA expression. * p<0.05. B. DNMT and TET expression before and after differentiation of NSC30, expressed as in Fig S7A. C-E. Expression of stem and lineage markers (C), DNMTs (D) and TETs (E) in GSCs. Expression is normalized to DYNLL and expressed as the mean+SEM. * P<0.05. X axis denotes cell line. Y axis shows the normalized (to DYNLL) mRNA expression. F. Representative images of TET1, TET2, and TET3 (red) in NSC30 (top panel) and GSC84 (bottom panel) in stem culture conditions. Scale bar: 200µm.
Fig S7

## Slide 17
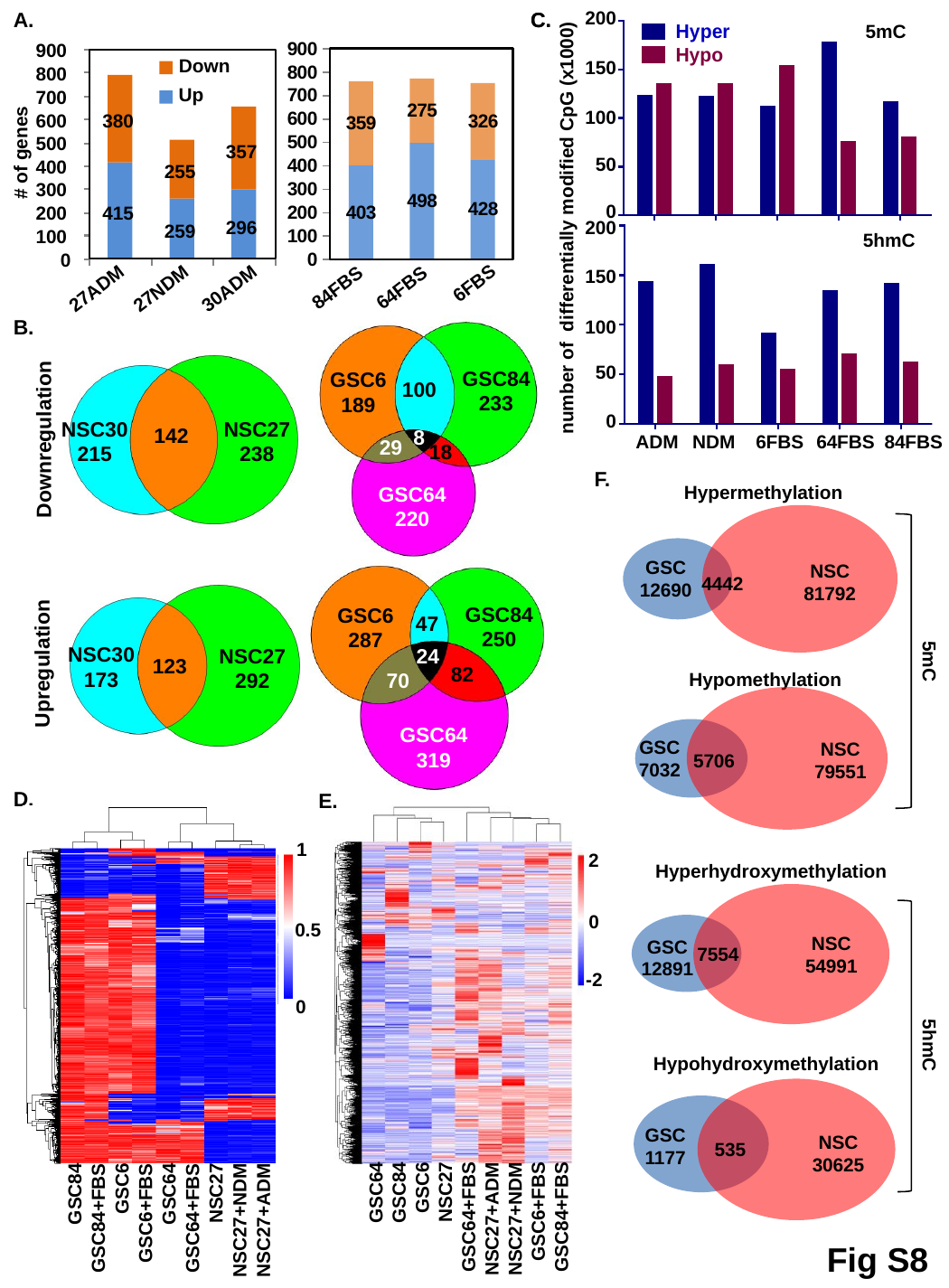

A.
C.
C.
200
5mC
### Chart
| Category | Up | Dn |
|---|---|---|
| 84FBS | 403.0 | 359.0 |
| 64FBS | 498.0 | 275.0 |
| 6FBS | 428.0 | 326.0 |
900
800
700
600
380
500
357
400
255
300
415
200
296
259
100
0
150
Down
Up
100
# of genes
50
0
200
number of differentially modified CpG (x1000)
5hmC
150
6FBS
84FBS
64FBS
27ADM
27NDM
30ADM
B.
100
GSC84
233
GSC6
189
100
8
29
18
GSC64
220
NSC30
215
NSC27
238
142
Downregulation
NSC30
173
NSC27
292
Upregulation
123
50
0
ADM
NDM
6FBS
64FBS
84FBS
F.
Hypermethylation
GSC
12690
NSC
81792
4442
5mC
Hypomethylation
GSC
7032
NSC
79551
5706
Hyperhydroxymethylation
NSC
54991
GSC
12891
7554
5hmC
Hypohydroxymethylation
GSC
1177
NSC
30625
535
GSC84
250
GSC6287
47
24
82
70
GSC64
319
D.
E.
GSC64
GSC84
GSC6
NSC27
GSC64+FBS
GSC6+FBS
GSC84+FBS
NSC27+ADM
NSC27+NDM
2
0
-2
GSC84
GSC6
GSC64
NSC27
GSC84+FBS
GSC6+FBS
GSC64+FBS
NSC27+NDM
NSC27+ADM
1
0.5
0
Fig S8

## Slide 18
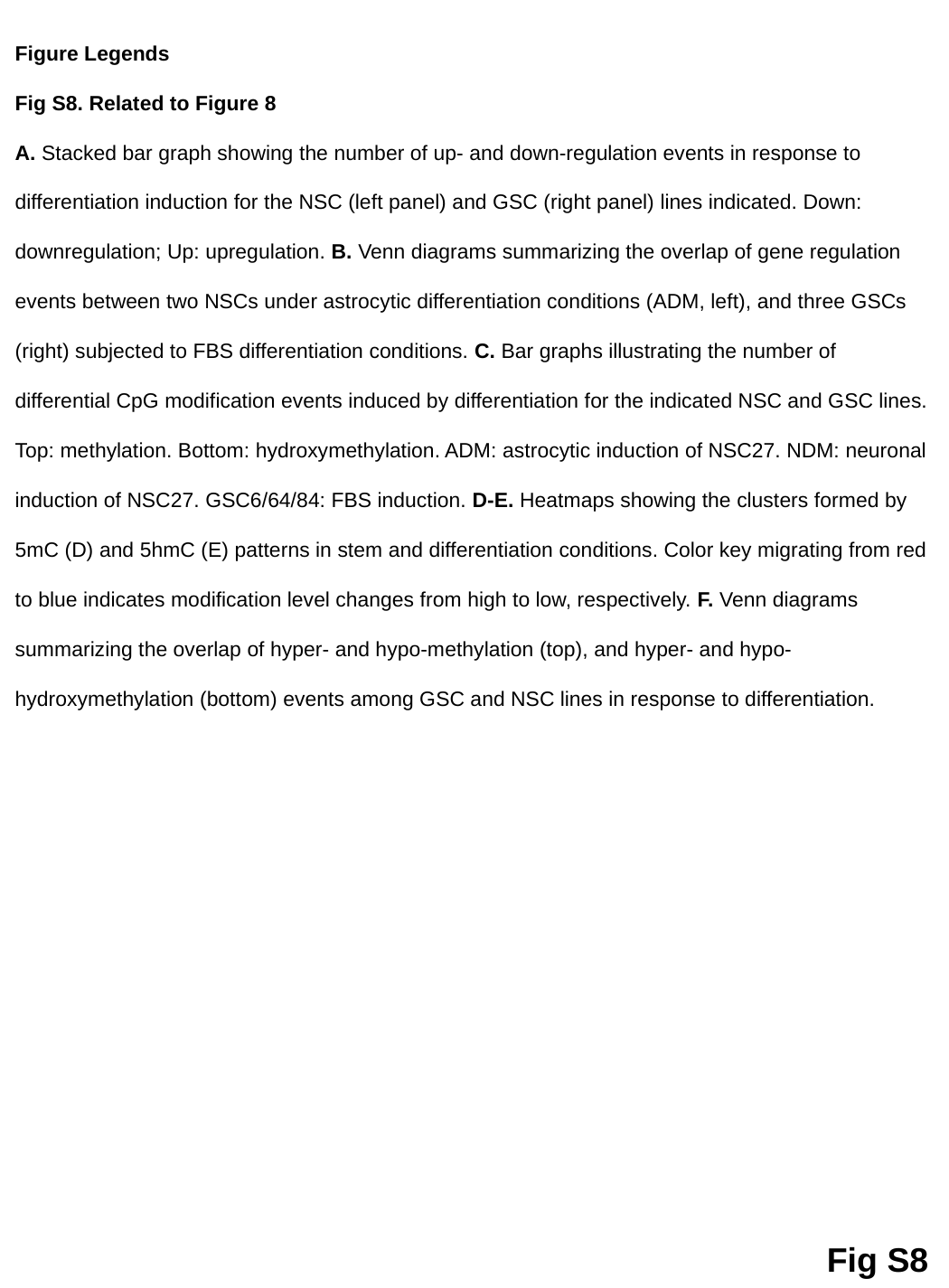

Figure Legends
Fig S8. Related to Figure 8
A. Stacked bar graph showing the number of up- and down-regulation events in response to differentiation induction for the NSC (left panel) and GSC (right panel) lines indicated. Down: downregulation; Up: upregulation. B. Venn diagrams summarizing the overlap of gene regulation events between two NSCs under astrocytic differentiation conditions (ADM, left), and three GSCs (right) subjected to FBS differentiation conditions. C. Bar graphs illustrating the number of differential CpG modification events induced by differentiation for the indicated NSC and GSC lines. Top: methylation. Bottom: hydroxymethylation. ADM: astrocytic induction of NSC27. NDM: neuronal induction of NSC27. GSC6/64/84: FBS induction. D-E. Heatmaps showing the clusters formed by 5mC (D) and 5hmC (E) patterns in stem and differentiation conditions. Color key migrating from red to blue indicates modification level changes from high to low, respectively. F. Venn diagrams summarizing the overlap of hyper- and hypo-methylation (top), and hyper- and hypo-hydroxymethylation (bottom) events among GSC and NSC lines in response to differentiation.
Fig S8

## Slide 19
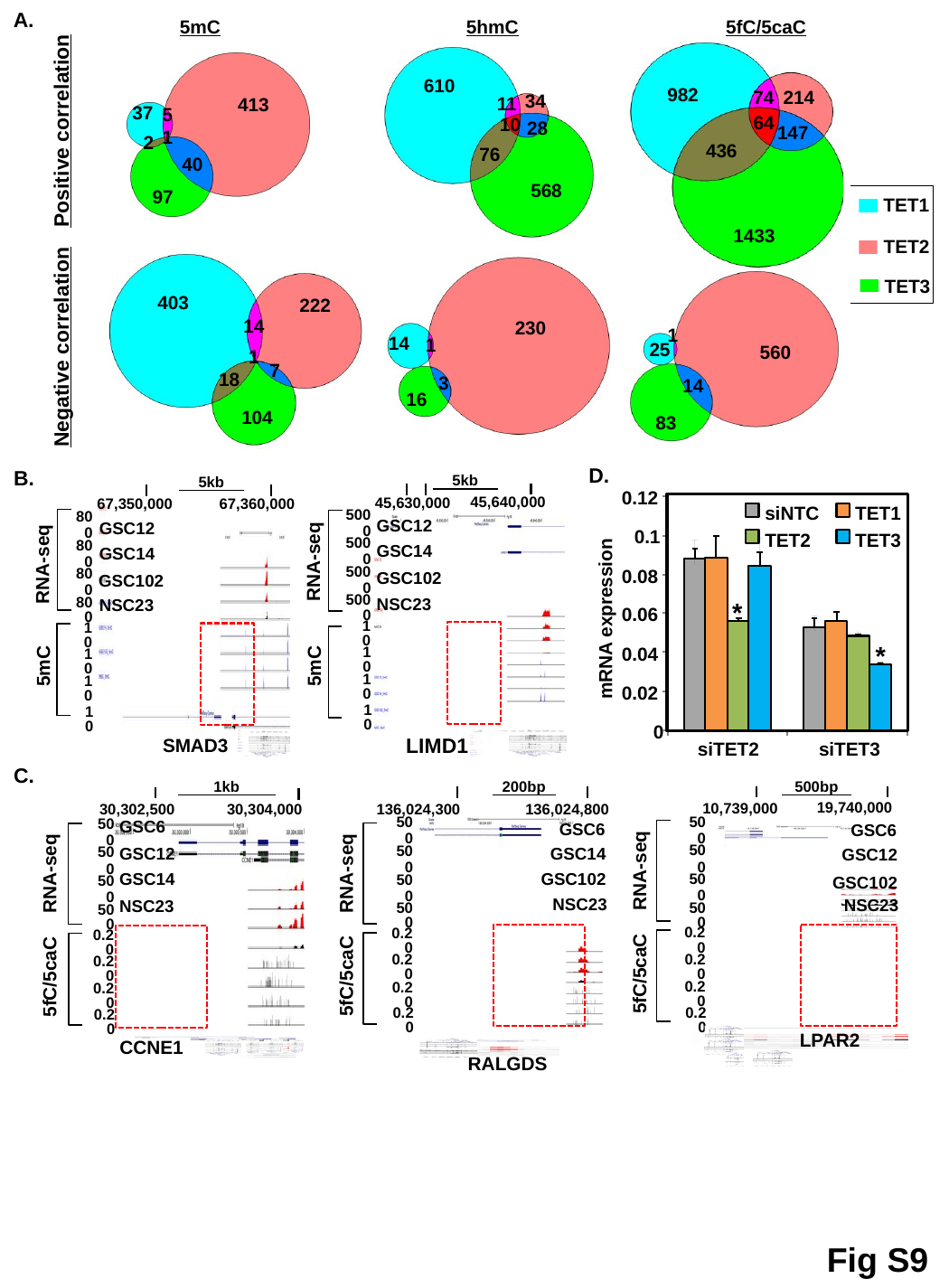

A.
5mC
5hmC
5fC/5caC
982
214
74
64
147
436
1433
610
34
11
10
28
76
568
413
37
5
1
2
40
97
Positive correlation
TET1
TET2
TET3
403
222
14
1
7
18
104
230
14
1
3
16
1
25
560
14
83
Negative correlation
D.
B.
5kb
5kb
0.12
0.1
0.08
*
0.06
*
0.04
0.02
0
siTET2
siTET3
mRNA expression
siNTC
TET1
TET2
TET3
45,640,000
45,630,000
67,360,000
67,350,000
500
0
500
0
500
0
500
0
1
0
1
0
1
0
1
0
80
0
GSC12
GSC14
GSC102
NSC23
LIMD1
GSC12
80
0
GSC14
RNA-seq
RNA-seq
80
0
GSC102
80
0
NSC23
1
0
1
0
5mC
5mC
1
0
1
0
SMAD3
C.
1kb
200bp
136,024,800
136,024,300
50
0
GSC6
50
0
GSC14
RNA-seq
GSC102
50
0
NSC23
50
0
0.2
0
0.2
0
5fC/5caC
0.2
0
0.2
0
RALGDS
500bp
19,740,000
10,739,000
50
0
GSC6
50
0
GSC12
50
0
GSC102
NSC23
50
0
0.2
0
0.2
0
0.2
0
0.2
0
LPAR2
RNA-seq
5fC/5caC
30,302,500
30,304,000
50
0
GSC6
50
0
GSC12
RNA-seq
GSC14
50
0
NSC23
50
0
0.2
0
0.2
0
5fC/5caC
0.2
0
0.2
0
CCNE1
Fig S9

## Slide 20
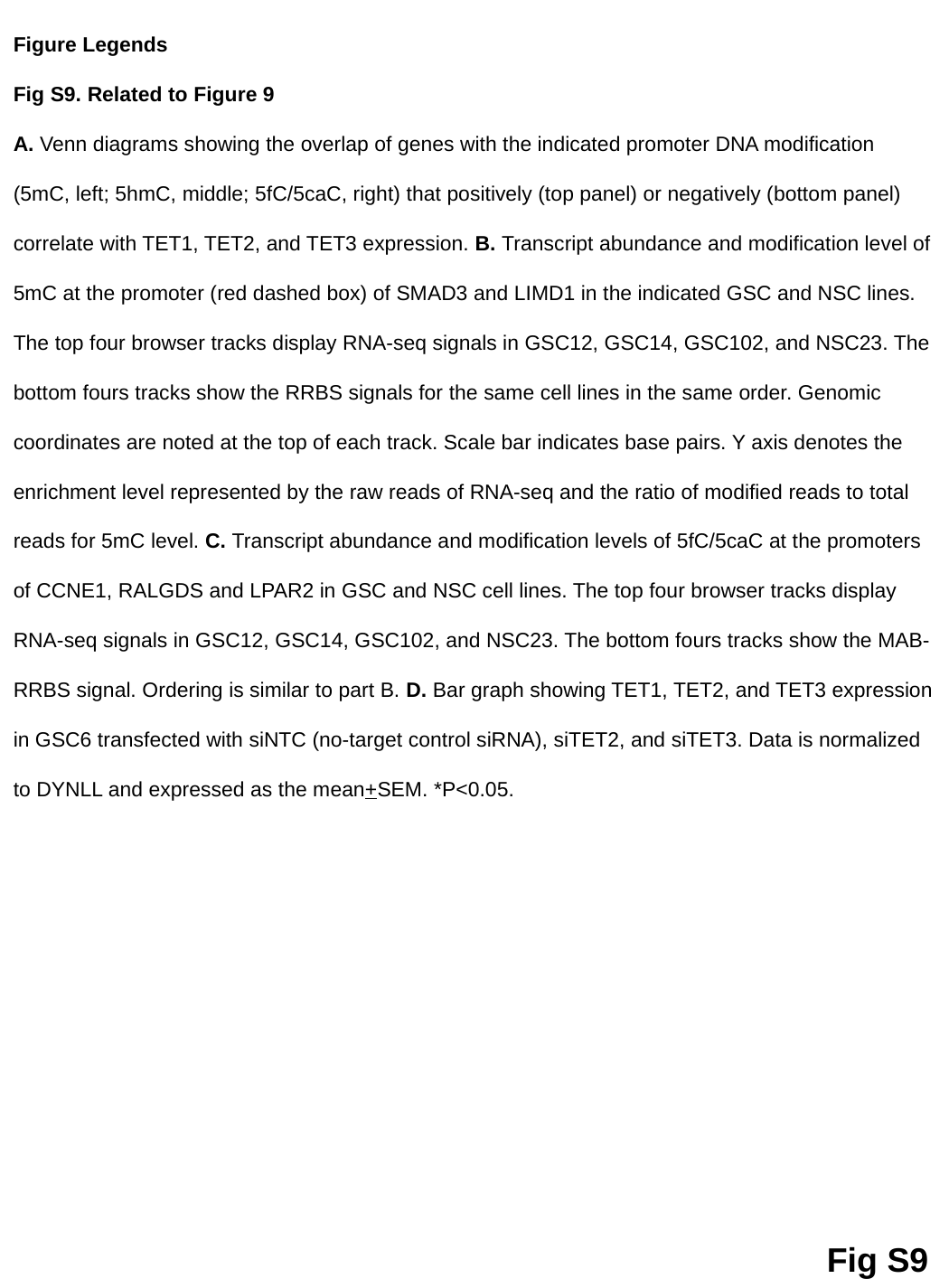

Figure Legends
Fig S9. Related to Figure 9
A. Venn diagrams showing the overlap of genes with the indicated promoter DNA modification (5mC, left; 5hmC, middle; 5fC/5caC, right) that positively (top panel) or negatively (bottom panel) correlate with TET1, TET2, and TET3 expression. B. Transcript abundance and modification level of 5mC at the promoter (red dashed box) of SMAD3 and LIMD1 in the indicated GSC and NSC lines. The top four browser tracks display RNA-seq signals in GSC12, GSC14, GSC102, and NSC23. The bottom fours tracks show the RRBS signals for the same cell lines in the same order. Genomic coordinates are noted at the top of each track. Scale bar indicates base pairs. Y axis denotes the enrichment level represented by the raw reads of RNA-seq and the ratio of modified reads to total reads for 5mC level. C. Transcript abundance and modification levels of 5fC/5caC at the promoters of CCNE1, RALGDS and LPAR2 in GSC and NSC cell lines. The top four browser tracks display RNA-seq signals in GSC12, GSC14, GSC102, and NSC23. The bottom fours tracks show the MAB-RRBS signal. Ordering is similar to part B. D. Bar graph showing TET1, TET2, and TET3 expression in GSC6 transfected with siNTC (no-target control siRNA), siTET2, and siTET3. Data is normalized to DYNLL and expressed as the mean+SEM. *P<0.05.
Fig S9

## Slide 21
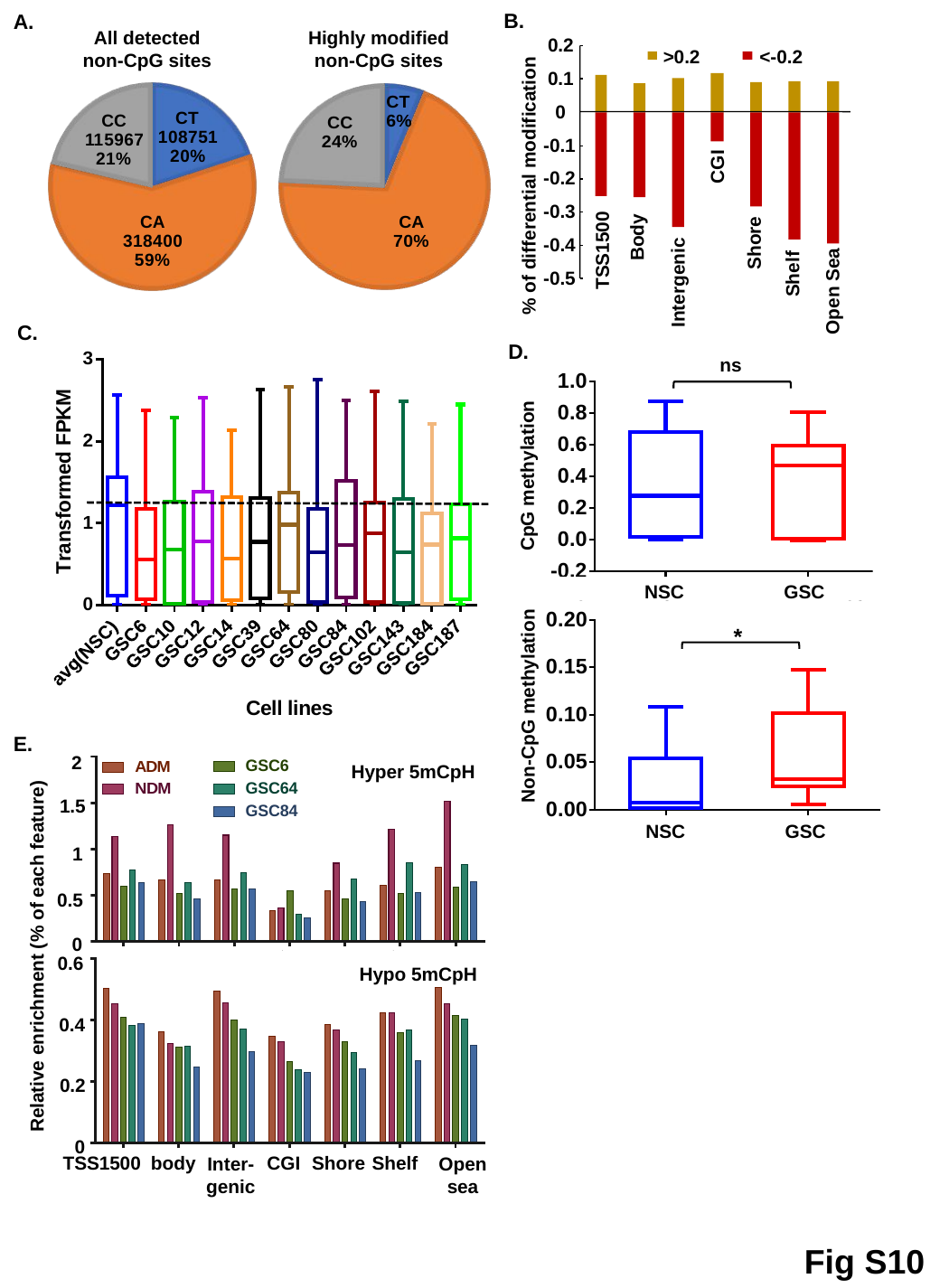

B.
A.
All detected non-CpG sites
Highly modified non-CpG sites
0.2
<-0.2
>0.2
0.1
0
-0.1
-0.2
% of differential modification
CGI
-0.3
-0.4
TSS1500
Body
Shore
-0.5
Shelf
Intergenic
Open Sea
C.
D.
ns
CpG methylation
*
Non-CpG methylation
NSC
GSC
NSC
GSC
E.
2
Hyper 5mCpH
1.5
1
0.5
0
Relative enrichment (% of each feature)
0.6
Hypo 5mCpH
0.4
0.2
0
TSS1500
body
CGI
Shore
Shelf
Inter-
genic
Open sea
Fig S10

## Slide 22
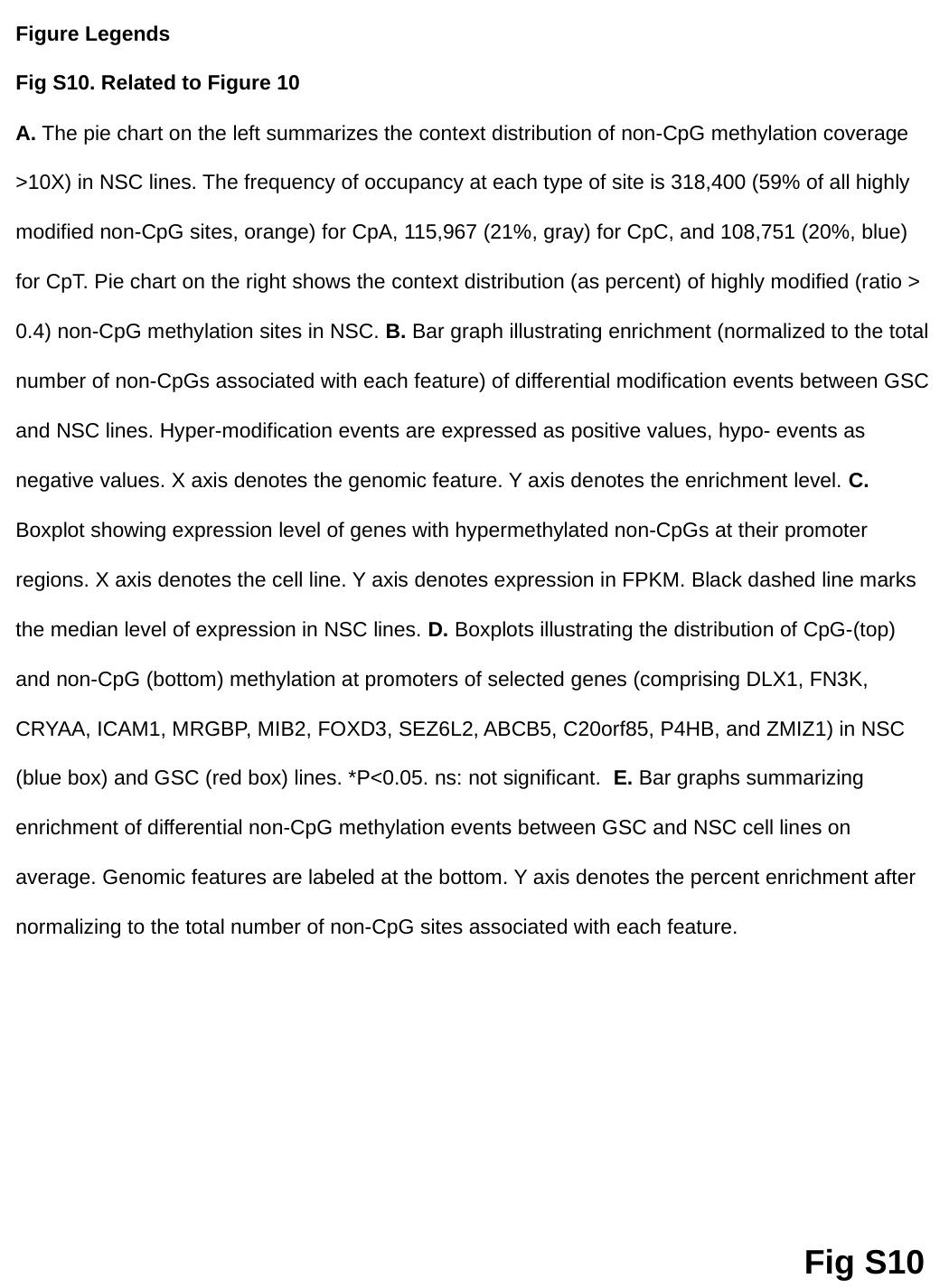

Figure Legends
Fig S10. Related to Figure 10
A. The pie chart on the left summarizes the context distribution of non-CpG methylation coverage >10X) in NSC lines. The frequency of occupancy at each type of site is 318,400 (59% of all highly modified non-CpG sites, orange) for CpA, 115,967 (21%, gray) for CpC, and 108,751 (20%, blue) for CpT. Pie chart on the right shows the context distribution (as percent) of highly modified (ratio > 0.4) non-CpG methylation sites in NSC. B. Bar graph illustrating enrichment (normalized to the total number of non-CpGs associated with each feature) of differential modification events between GSC and NSC lines. Hyper-modification events are expressed as positive values, hypo- events as negative values. X axis denotes the genomic feature. Y axis denotes the enrichment level. C. Boxplot showing expression level of genes with hypermethylated non-CpGs at their promoter regions. X axis denotes the cell line. Y axis denotes expression in FPKM. Black dashed line marks the median level of expression in NSC lines. D. Boxplots illustrating the distribution of CpG-(top) and non-CpG (bottom) methylation at promoters of selected genes (comprising DLX1, FN3K, CRYAA, ICAM1, MRGBP, MIB2, FOXD3, SEZ6L2, ABCB5, C20orf85, P4HB, and ZMIZ1) in NSC (blue box) and GSC (red box) lines. *P<0.05. ns: not significant. E. Bar graphs summarizing enrichment of differential non-CpG methylation events between GSC and NSC cell lines on average. Genomic features are labeled at the bottom. Y axis denotes the percent enrichment after normalizing to the total number of non-CpG sites associated with each feature.
Fig S10
